# Robust Photocleavable Linkers for DNA Synthesis: Enabling Visible Light-Triggered Antisense Oligonucleotide Release in 3D DNA Nanocages

**DOI:** 10.1021/acs.biomac.5c00162

**Published:** 2025-04-24

**Authors:** Hoi Man Leung, Hau Yi Chan, Maxime Klimezak, Ling Sum Liu, Pierre Karam, Alexandre Specht, Frédéric Bolze, Pik Kwan Lo

**Affiliations:** † Department of Chemistry and State Key Laboratory of Marine Pollution, City University of Hong Kong, Tat Chee Avenue, Kowloon Tong, Hong Kong, China; ‡ Laboratoire de Chémo-Biologie Synthétique et Thérapeutique (CBST), Équipe Nanoparticules Intelligentes, CNRS, CBST UMR 7199, Université de Strasbourg, Illkirch, Cedex F-67401, France; § Department of Chemistry, American University of Beirut, Beirut 1107 2020, Lebanon; ∥ Key Laboratory of Biochip Technology, Biotech and Health Care, Shenzhen Research Institute of City University of Hong Kong, Shenzhen 518057, China

## Abstract

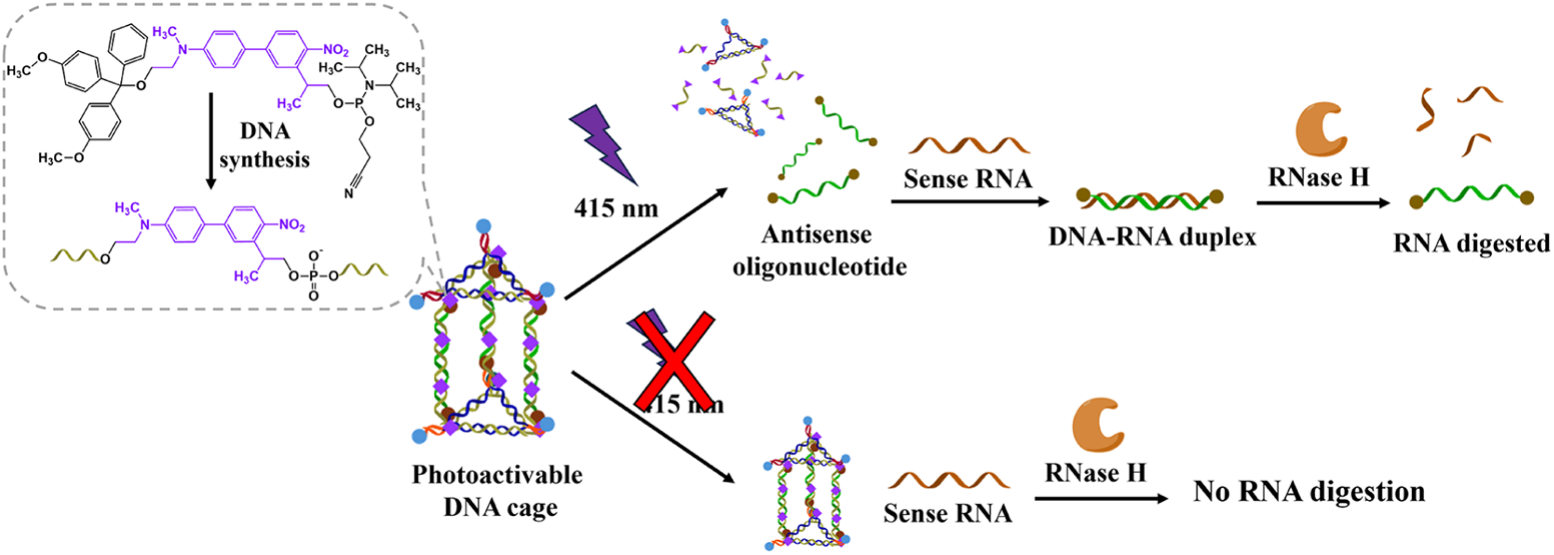

We synthesized new *para*-dialkylaminonitrobiphenyl (ANBP) derivatives, s-ANBP and t-ANBP, functionalized with dimethyltrityl and phosphoramidite groups for incorporation into DNA backbones as photocleavable linkers via solid-phase synthesis. Both derivatives exhibited excellent chemical stability under diverse conditions, including acidic, alkaline, and high-salt environments and elevated temperatures. Their incorporation into DNA influenced duplex stability and antisense oligonucleotide (ASO) dissociation efficiency, depending on the number of ANBP units and adjacent nucleotide deletions. The s-/t-ANBP-conjugated DNA showed efficient one-photon photolysis at 415 nm and enhanced two-photon absorption for extended π-system in *t*-ANBP, with δ_u_Φ_u_ values of 1.6 GM (740 nm) and 2.7 GM (800 nm). ANBP-conjugated DNA was used to construct a 3D DNA nanocage capable of light-triggered ASO 4625 release, validated by an *in vitro* RNA digestion assay, confirming antisense functionality. This platform demonstrates precise, light-mediated therapeutic delivery and offers potential for broader applications in drug delivery and clinical use.

## Introduction

Light-responsive nucleic acids are invaluable tools in scientific research, offering precise, noninvasive, and remote control over biological processes.^1^ Their unique capabilities enable researchers to explore gene regulation, protein function, molecular assembly, and therapeutic interventions with exceptional spatial and temporal resolution.^2^ Since nucleic acids are naturally unresponsive to light, various chemical strategies have been developed to incorporate photoresponsive molecules into their structure. These molecules include photoisomerization compounds, photocages, photocleavage linkers, and photo-cross-linking groups, which can be integrated at specific positions within the nucleic acid sequence to enable diverse light-activated functionalities. A pioneering study by Ordoukhanian and Taylor introduced *o*-nitrobenzyl (*o*NB) esters into duplex DNA to facilitate phototriggered strand breaks.^3^ Since then, *o*NB and its derivatives have been extensively applied in the development of light-responsive nucleic acid tools. These tools have been employed in applications such as biosensing,^4,5^ gene editing,^6^ protein function regulation,^7^*in vivo* gene control,^8,9^ and immunotherapy,^10^ with activation triggered by UV or near-UV light.

Besides the photochemical regulation of duplex oligonucleotides, a high-order photoresponsive self-assembled DNA-based system has recently been explored. Photocleavable *o*NB-functionalized DNAs have been widely used for the development of photoresponsive DNA nanomaterials for structural switching and property tuning.^11^ It is important to note that the position at which these photocleavable molecules are introduced can significantly influence their effects on the nucleic acid’s structure and function. For example, *o*NB-inserted nucleic acids have been used as building blocks to assemble 3D DNA nanostructures in the form of nanotubes,^12^ spheres,^13^ and bipyramids.^14^ These nanostructures were locked together on one side by *o*NB-inserted DNA strands and permanently linked by regular DNA on the opposite side. UV-induced bond cleavage of the photocleavable groups caused an irreversible structural transition, from the closed to open state, leading to the potential release of the internal cargo. Furthermore, the use of 3D photoresponsive DNA ring^15^ and icosahedral DNA nanocapsule^16^ as controllable nanocarriers to deliver large proteins and neurosteroids, respectively. In Endo’s study, photocleavable strands that bridge between the anchoring strands and the single-guide RNA (sgRNA) on the Cas9 protein were employed to entrap the protein inside the nanocavity of the DNA ring.^15^ Irradiating the ring structure at 350 nm under 300 W for 5 min cleaved the bridging strand, leading to the release of Cas9 for site-specific sgRNA-guided cleavage of the target duplex substrate. Furthermore, *o*NB-inserted nucleic acids were also applied to engineer DNA microcapsules and DNA micelles. Willner’s group reported the fabrication of DNA microcapsule by sequential deposition of photocleavable DNA layers onto a prefabricated calcium carbonate microparticle containing the desired loads, such as fluorophores and anticancer drugs.^17^ Irradiation with UV light induces the breakdown of the overall microcapsule shells, resulting in the release of trapped cargoes. Tan’s group also designed DNA micelles with adjustable stability using photocontrollable dissociation of an intermolecular G-quadruplex.^18^ An *o*NB molecule was inserted into the DNA hairpin loop as part of the nucleic acid backbone. Once exposed to UV light, the *o*NB molecule broke, opening the hairpin loop and subsequently releasing the C-rich sequence, which blocked the formation of the G-quadruplex by strand hybridization. This photocleavage causes the DNA micelles to lose stability in the event of cellular uptake and serum albumin interference.

Alternatively, Stephanopoulos’s group reported the use of a nitropiperonyloxymethyl (NOPM) moiety as a photoremovable protecting group (PPG) to protect a poly T strand, which was initially incorporated into a DNA nanotweezer in a closed state. Upon 365 nm UV light irradiation under 18.2 W for 15 s, the NOPM moieties were cleaved, unmasking the hydrogen bonding sites associated with the thymidine residues for hybridization with the corresponding hairpin loop to generate the open state of the tweezer.^19^ The Heckel group also protected the deoxyguanosine and deoxycytidine with 2-(2-nitrophenyl)-propyl group and 1-(2-nitrophenyl)-ethyl group, respectively, of a stem in an individual DNA minicircle. Upon irradiation with UV light, the cage compounds were cleaved off, and the stem strands could hybridize to another deprotected DNA minicircle to form a dimer via Watson–Crick base pairing or G-quadruplex formation in the presence of Na^+^ ions.^20^ Furthermore, the idea of end-capping photoresponsive molecules in self-assembled nucleic acid nanostructures offers another strategy for drug delivery and motion. Specifically, phototriggered release of anticancer drugs,^21^ proteins^22^ and Immunoglobulin G (IgG) molecules^23^ was achieved by breaking the chemical bonding between the cargo and the photocleavable *o*NB group, leading to the dissociation of the drugs from the DNA nanocarrier.

Although significant progress has been made in the photochemical manipulation of self-assembled DNA nanostructures, research on photoresponsive nucleic acid nanostructures predominantly concentrates on the transportation of small molecules, with limited discourse on gene delivery designs. Furthermore, the majority of these methodologies is constrained to UV light activation. Notably, the liberation rates of *o*NB and its analogs are relatively sluggish, and postillumination, the formation of byproducts like nitrosoaldehyde can undergo adverse reactions with amines. This interaction may prove detrimental to adjacent proteins, potentially instigating cytotoxic repercussions.^24^

To address these challenges, we were motivated to synthesize new stable, visible-light-photocleavable phosphoramidites for DNA conjugation in a cost-efficient manner. While (2,7-bis-{4-nitro-8-[3-(2-propyl)styryl]}-9,9-bis-[1-(3,6-dioxaheptyl)]-fluorene (BNSF) and (4,4’-bis-{8-[4-nitro-3-(2-propyl)-styryl]}-3,3′-dimethoxybiphenyl (BNSMB) derivatives have been reported as efficient photoreleasing tools for glutamate and polymers, they also show a promising two-photon uncaging cross-section at a wavelength of 800 nm, which is beneficial for efficient photolysis reactions.^25^ However, the chemical syntheses reported by Nicoud and coworkers cannot be easily adapted for the preparation of phosphoramidites suitable for oligonucleotide conjugation.^26^ In response to this limitation, our group recently synthesized two phosphoramidites based on the BNSF and BNSMB structures, designed as visible-light-cleavable linkers for the development of a series of photon-activated DNA logic gates. Unfortunately, we found that incorporating BNSF and BNSMB derivatives into the DNA backbone significantly destabilized the formation of higher-order self-assembled DNA nanostructures due to their symmetrical and bulky features.

In this study, we developed a *p*-dialkylaminonitrobiphenyl (ANBP) derivative-functionalized DNA nanocage as a novel platform for the phototriggered release of antisense oligonucleotide (ASO), with potential applications in gene therapy. This system represents a significant advancement in the development of light-responsive systems for the precise and controllable release of therapeutic drugs. The ANBP molecule, a derivative of the *o*-nitrophenylpropyl family, features a donor–acceptor biphenyl core and a dimethylamino substituent at the para position,^27,28^ exhibiting remarkable photophysical and photochemical properties under both single- and two-photon excitation.^29,30^ This enables the efficient release of carboxylate,^25,26,31^ phenol,^32^ and phosphate groups,^33^ showcasing its versatility. Despite the promising features of ANBP derivatives, their chemical functionality has posed challenges when it comes to backbone insertion along DNA strands. Presently, these derivatives are monofunctional, primarily substituting for guanine, which limits their applicability due to experimental and synthetic complexities. To address this limitation, we designed and synthesized new ANBP derivatives (s-ANBP) equipped with dimethyltrityl and phosphoramidite groups to enable their integration into DNA backbones. These modifications allow for solid-phase synthesis and extend the versatility of ANBP molecules. To enhance the two-photon absorption property by extending the length of the conjugated system,^34^ a triple-bond version of the ANBP derivative was further developed (t-ANBP). Our study involved investigating the one-photon and two-photon uncaging properties of single-stranded ANBP-conjugated nucleic acids. The ANBP derivatives were integrated into DNA duplexes and DNA nanocages comprising antisense oligonucleotide 4625 (ASO 4625). The photorelease of ASO 4625 upon exposure to 415 nm light was studied, and an *in vitro* RNA digestion experiment was performed to demonstrate the light-responsive gene regulatory potential of our engineered ANBP-conjugated DNA nanocage.

## Experimental Section

### Materials

All chemicals and reagents were reagent grade and were utilized as received from Acros Organics, Alfa Aesar, Fluorochem, Sigma-Aldrich, Toronto Research Chemicals, or Tokyo Chemical Industry. Anhydrous solvents, including acetonitrile (MeCN), *N*,*N*-dimethylformamide (DMF), pyridine, tetrahydrofuran (THF), toluene (PhMe), and triethylamine (TEA), were used as received from Acros Organics, Alfa Aesar, Sigma-Aldrich, or Tokyo Chemical Industry. Deuterated solvents were used as received from Sigma-Aldrich. All other solvents, including dichloromethane (DCM), ethanol (EtOH), ethyl acetate (EtOAc), *n*-hexane, and petroleum ether 40–60 (PE 40–60), were technical grade. Flash column chromatography was performed on silica gel (60 Å, 40–63 μm) as the stationary phase. The progress of flash column chromatography was monitored by TLC silica gel 60 F254 plates, and the elutes were visualized under a 254 nm UV lamp. Acetic acid, acrylamide, ammonium citrate dibasic, ammonium hydroxide, bis-acrylamide, 2’, 6’-dihydroxyacetophenone, ethylenediaminetetraacetic acid (EDTA) disodium salt dihydrate, hydrochloric acid, and *N*, *N*’ methylenebis(acrylamide) were obtained from Sigma-Aldrich. Boric acid, magnesium chloride hexahydrate, and tris(hydroxymethyl)aminomethane (Tris base) were purchased from J&K Scientific. Nucleoside-derivatized LCAA-CPG solid support (1000 Å) with loading densities of 25–40 μmol/g and chemicals used for automated DNA synthesis were purchased from BioAutomation. Black hole quencher-1 (BHQ) CPG/phosphoramidite, Cy3 CPG, 6-carboxyfluorescein (FAM) CPG, and Spacer Phosphoramidite 9 were purchased from Glen Research. 40% acrylamide/bis-acrylamide solution in a ratio of 19:1 was purchased from Bio-Rad. Sephadex G-25 (super fine DNA grade) was used as purchased from Amersham Biosciences. RNase H was purchased from Beyotime Biotechnology. 1× TBE buffer was composed of 90 mM Tris base and boric acid with 1.1 mM EDTA, the pH was adjusted to 8.0 ± 0.1 using hydrochloric acid. The 1× TAMg buffer was composed of 40 mM Tris base, 12.5 mM magnesium chloride hexahydrate, and 20 mM acetic acid, with the pH adjusted to 8.0 ± 0.1.

### Instrumentation

Nuclear magnetic resonance (NMR) spectroscopy was analyzed on a Bruker AVANCE III (BBO probe) 400 MHz NMR spectrometer or a Bruker AVANCE III HD (BBO probe) 300 MHz NMR spectrometer. ^1^H and ^13^C NMR chemical shifts were reported in δ units, parts per million (ppm), relative to the chemical shift of residual solvents. ^11^B NMR chemical shifts were reported in δ units, ppm, relative to the boron trifluoride diethyl etherate (BF_3_–O(C_2_H_5_)_2_) as an external standard. ^31^P NMR chemical shifts were reported in δ units, ppm, relative to the phosphoric acid (H_3_PO_4_) as an external standard. ^13^C and ^31^P NMR measurements were executed and acquired with ^1^H-decoupling methodologies. Electrospray ionization mass spectrometry (ESI-MS) was analyzed on a PE-SCIEX API 3200 triple quadrupole mass spectrometer, monitored, and recorded in positive ion mode. Automated oligonucleotide solid-phase synthesis was performed on a BioAutomation MerMade MM6 DNA synthesizer. Matrix-assisted laser desorption/ionization-time-of-flight mass spectrometry (MALDI-TOF-MS) was analyzed on either an ABI 4800 Plus MALDI-TOF/TOF mass spectrometer or a Bruker autoflex maX MALDI-TOF/TOF mass spectrometer. Ammonium citrate dibasic and 2’,6’-dihydroxyacetophenone saturated in methanol (70%) were used as the matrix. UV–vis spectrometry was performed on a Thermo Scientific NanoDrop One or Agilent Cary 300 UV–vis spectrometer. Fluorescence emission measurements were performed on a Horiba FluoroMax 4 spectrofluorometer. Gel electrophoresis experiments were carried out on an acrylamide 20 cm × 20 cm Maxi Vertical electrophoresis apparatus (MV-20DSYS). Gel scanning was performed on a Fujifilm FLA-9000 scanner. Photolysis at 415 nm was performed using a 200 W LED lamp (UV TaoYuan LED source from Shenzhen Taoyuan Optoelectronics Co., Ltd.). High-performance liquid chromatography (HPLC) was performed on a Waters HPLC system, assembled with a Waters 1525 pump, an XBridge BEH C18 (5 mm, 4.6 × 150 mm) analytical column, and a Waters 2998 photodiode array detector. Analysis was conducted using gradient elution, starting from 100% 25 mM of triethylammonium acetate (TEAA) buffer and reaching a 100% mixture of MeCN/H_2_O (v/v = 1:1) in 30 min.

### Synthesis of Photocleavable DNA Oligonucleotides and Reference DNA Oligonucleotides

Oligonucleotide synthesis was performed on a 200 nmol scale using an automated oligonucleotide synthesizer and standard cyanoethylphosphoramidite chemistry, starting from the 1000 Å universal CPG solid support. Commercially available DNA nucleoside phosphoramidites and s-/t-ANBP phosphoramidites were site-specifically coupled onto the growing oligonucleotide chain as artificial bases with prolonged detritylation and coupling times. The coupling efficiency was monitored by measuring the trityl concentration level. The DNA oligonucleotides were deprotected in 30% ammonium hydroxide at 55 °C for 16 h.

### Purification of Photocleavable DNA Oligonucleotides and Reference DNA Oligonucleotides

DNA oligonucleotides were purified on 15% polyacrylamide/8 M urea polyacrylamide gels at a constant current of 30 mA for 3 h (30 min at 250 V followed by 2.5 h at 500 V), using 1x TBE buffer. For DNA containing fluorophores, target bands were cut and collected in a 15 mL centrifuge tube. For DNA without fluorophores, the plates were wrapped in plastic after electrophoresis and placed on a fluorescent TLC plate, and then illuminated with a UV lamp at 254 nm. The bands were excited quickly. The selected gel pieces were crushed and incubated in 12 mL of sterile water at 55 °C for 16 h. The samples were concentrated to about 1 mL using a speed vacuum concentrator and desalted using Sephadex G-25 column chromatography. Quantification was carried out by UV–vis analysis.

### HPLC Analysis of One-Photon Photolysis of Photocleavable DNA Oligonucleotides

Cy3-d15-s-/t-ANBP-d19-FAM, after irradiation with an LED, was subjected to a reverse-phase HPLC system with a Waters 2998 photodiode array detector. Analysis was done by using a gradient elution starting from 100% 25 mM triethylammonium acetate (TEAA) buffer to 100% acetonitrile/water (v/v = 1:1) in 30 min. The signal of FAM was monitored at 495 nm, and the signal of Cy3 was monitored at 550 nm. Subsequently, the quantification of photolytic products was calculated from the peak area correlated to the calibration curve of d19-FAM.

### Photolysis Quantum Yield

The quantum yield for the photoconversion was determined in MeOH/H_2_O 9:1 in volume ratio at 25 °C in a 1 mL quartz cuvette by comparison with the DEACAS-*p*-methoxybenzoic (Φ = 0.2)^35^ at the same concentration (20 μM) as the reference. For the light source, LUMOS 43 LED equipment (from Atlas Photonics Inc.) was used in the 405 nm irradiation mode (typical optical output: 200 mW/cm^2^). The reaction was monitored by UV spectroscopy on 2 mL 20 μM solutions. To determine the extent of the photolytic conversions, difference spectra (tirr-t0) were used. The absorbance evolution at 450 and 420 nm for DEACAS-*p*-methoxybenzoic and s-ANBP, t-ANBP , respectively, was plotted to follow the uncaging kinetics (*k*_sample_ for s-ANBP and t-ANBP, and *k*_ref_ for DEACAS-*p*-methoxybenzoic reference molecule), and the values were normalized. According to the following equation, the ratio of the rate constants was directly proportional to the one-photon uncaging sensitivities of the new compound (ε_sample at 405 nm_ × Φ_sample_) and the reference compound (ε_ref at 405 nm_ × Φ_ref_):
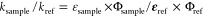
1

### Two-Photon Uncaging Action Cross-Section Determination

Two-photon uncaging action cross-section experiments were performed on our homemade setup at the plateforme d’imagerie Quatitative (PIQ-QuESt) at the Faculty of Pharmacy (University of Strasbourg), as described recently.^36^ The excitation source was a femtosecond laser (Insight Spectra-Physics, 680–1300 nm) with a pulse width of <120 fs and a repetition rate of 80 MHz. The reference and the sample were dissolved in MeOH/H_2_O 9:1 in a volume ratio, with the absorbance of both solutions close to 0.4 (at λ_max_). The kinetics of the two-photon process of the compound of interest, compared to the reference, was studied using 75 μL of solutions (both reference and sample alternately) irradiated and analyzed by UV–vis spectroscopy after different times of irradiation, without modifying the laser excitation settings, with uncaging percentages lower than 15% to avoid interferences due to photolysis byproducts.

### Assembly of the ANBP Cage

Equimolar amounts of FAM-labeled T1a, T1b, and T1c strands (0.07 nmol) were mixed in 1X TAMg buffer to generate a discrete FAM-labeled T1 product. FAM-labeled T2 was formed using the same strategy by adding equimolar amounts of FAM-labeled T2a, T2b, and T2c strands in 1X TAMg buffer. Three times the equivalent amount of LS-ANBP (3x) was mixed with T2 to yield an intermediate of LS-bound T2, followed by the addition of T1. Finally, three times the equivalent amount of 4625-BHQ was added to produce a fully hybridized ANBP-Cage. The mixture was incubated at 4 °C for 15 min after the addition at each step.

### Native Polyacrylamide Gel Electrophoresis

20 μL portion of ANBP-conjugated DNA at a concentration of 3.5 μM was mixed with 6 μL of 70% glycerol in water. The samples were loaded into a native PAGE gel in a running buffer of 1X TAMg. The fluorescence of FAM was detected upon excitation at 488 nm, and the gel was stained with Stains-All solution for DNA band visualization.

### Transmission Electron Microscopy

10 μL of ANBP-Cage at a concentration of 3.5 μM was deposited on a carbon-coated copper grid for 30 min. Excess liquid was removed with a piece of filter paper after the sample incubation. The grid was stained with NanoVan for 1 min and washed with deionized water for 1 min. It was roughly dried with a piece of filter paper and dried in a desiccator overnight. TEM imaging was conducted by a transmission electron microscope (Philips Tecnai 12).

## Results and Discussion

### Synthesis of ANBP Phosphoramidites

**Scheme 1:**
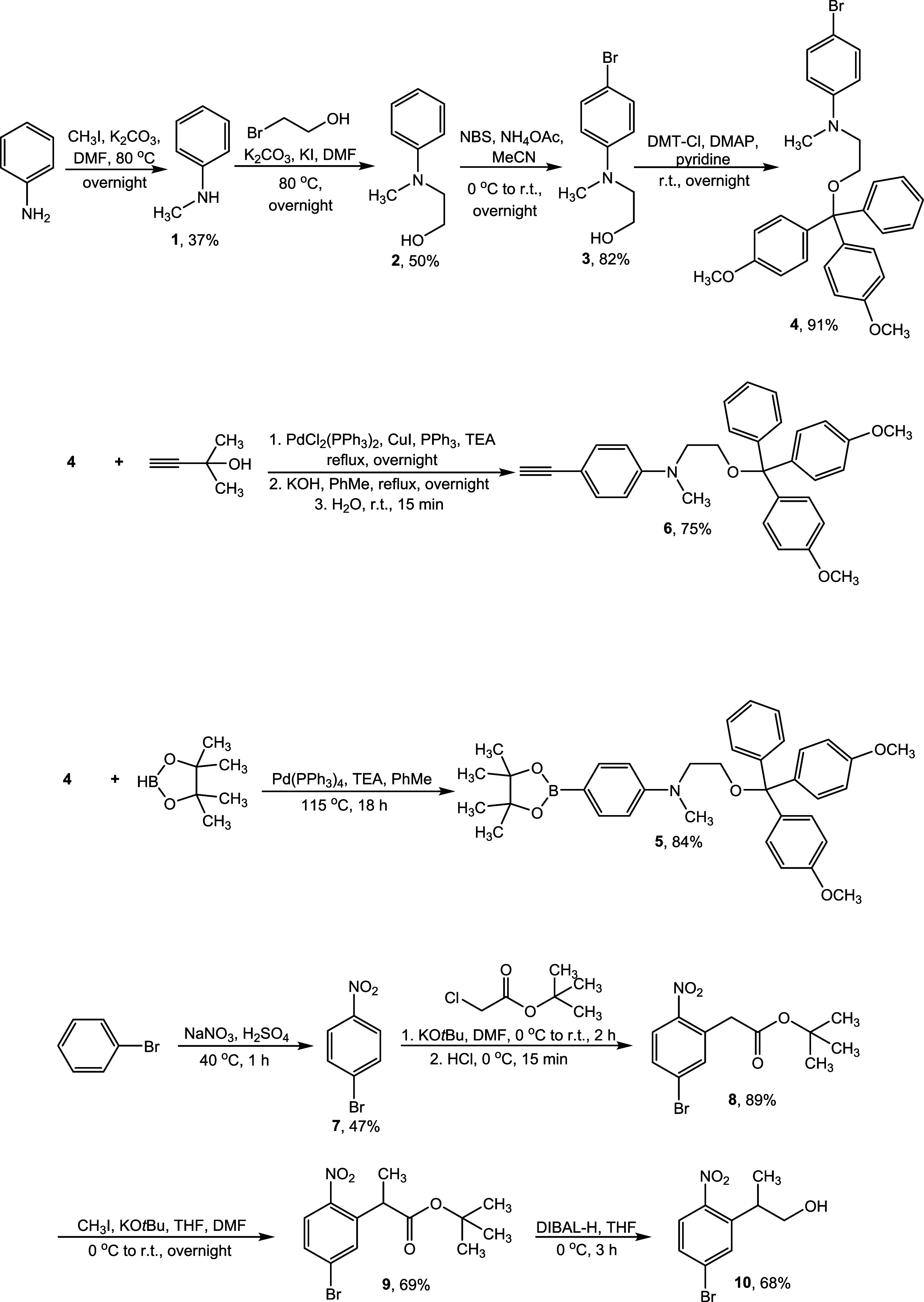
Synthesis of Electron Donating Motifs **4**, **5**, and **6**, and Electron Accepting *Ortho*-Nitrobenzyl Motif **10**

Synthesis began with the construction of electron-donating motifs **4**, **5**, and **6**, as shown in Scheme 1. Starting from aniline, sequential methylation and alkylation of the amino group gave 2-(methylphenylamino)ethanol **2**. Bromination at the *para*-position of the aromatic ring using *N*-bromosuccinimide yielded aryl bromide **3**. In the next step, the aliphatic alcohol group in compound **3** was protected with 4,4’-dimethoxytrityl chloride (DMT-Cl), affording the tritylated product **4**. Palladium (Pd)-mediated borylation of aryl bromide **4** with pinacolborane produced boronate ester **5**. Alternatively, a Sonogashira reaction of aryl bromide **4** with dimethyl ethynyl carbinol, in the presence of Pd and copper (Cu) catalysts, introduced an alkyne motif to the substrate. Subsequently, removal of the dimethyl carbinol group using hydroxide ions, followed by protonation in water, afforded terminal alkyne **6**. To generate the electron-accepting *ortho*-nitrobenzyl motif, aromatic nitration at the *para*-position was performed by treating bromobenzene with sodium nitrate in an acidic medium, yielding 1-bromo-4-nitrobenzene **7**. Vicarious nucleophilic substitution at the *ortho*-position relative to the nitro group, using an ester derivative and potassium *tert*-butoxide as a base, furnished compound **8**. Methylation of the benzylic carbon in compound **8**, followed by carbonyl reduction with diisobutylaluminum hydride (DIBAL-H) resulted in the *o*-nitrobenzyl intermediate **10**.

**Scheme 2:**
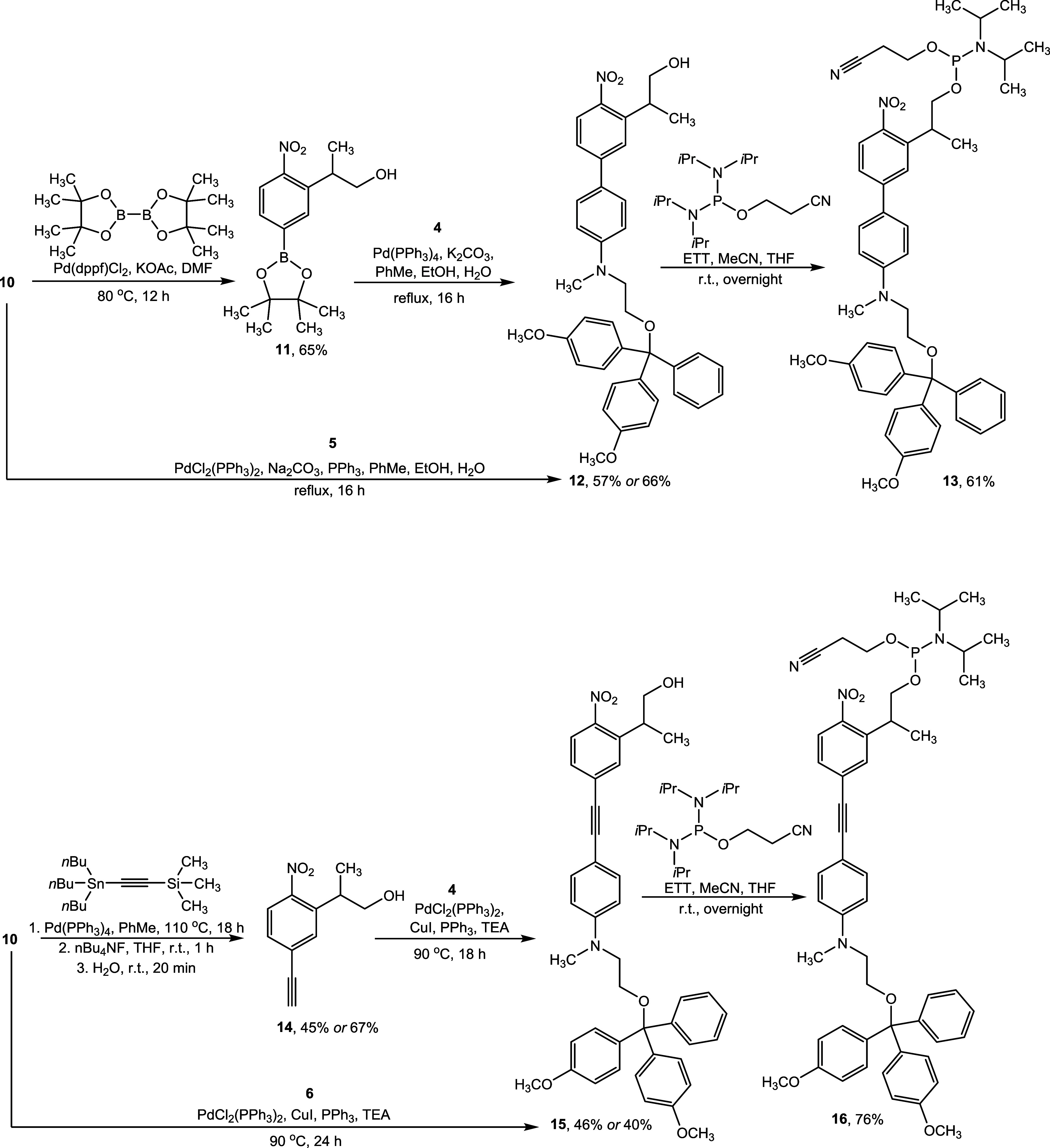
Synthesis of s-ANBP-Phosphoramidite **13** and t-ANBP-Phosphoramidite **16**

To construct the π-conjugated cores, two synthetic approaches were employed, both involving various cross-coupling reactions to link the corresponding electron-accepting and electron-donating motifs, as shown in Scheme 2. Miyaura borylation of *o*-nitrobenzyl bromide derivative **10** with bis(pinacolato)diboron afforded the corresponding boronate ester **11**. The biphenyl core **12** was constructed by performing a Suzuki reaction between aryl bromide **4** and boronate ester **11** in the presence of Pd catalysts and alkali metal carbonates. Alternatively, biphenyl core **12** could also be synthesized by reacting compound **10** with boronate ester **5** under similar coupling conditions. Finally, compound **12** was phosphorylated with 2-cyanoethyl *N,N,N’,N’*-tetraisopropylphosphorodiamidite, using 5-(ethylthio)-1*H*-tetrazole (ETT) as the activator, to yield s-ANBP-phosphoramidite **13**.

To synthesize the triple bond-functionalized ANBP phosphoramidite, a Stille reaction was performed on intermediate **10** by treating it with tributyl(trimethylsilylethynyl)tin and a Pd catalyst. Subsequent removal of the trimethylsilyl group using a fluoride ion, followed by protonation of the resulting anionic acetylide in water, afforded terminal alkyne **14**. The internal alkyne **15** was then constructed via a Sonogashira reaction between aryl bromide **4** and terminal alkyne **14** in the presence of Pd and Cu catalysts. Alternatively, compound **15** could also be synthesized by reacting compound **10** with terminal alkyne **6** under similar Sonogashira coupling conditions. Finally, compound **15** was phosphorylated to afford t-ANBP-phosphoramidite **16**. All intermediates and final compounds were characterized by ^1^H, ^13^C, ^11^B, and/or ^31^P nuclear magnetic resonance (NMR) spectroscopies, as well as electrospray ionization mass spectrometry (ESI-MS) (Figures S1–S34). The data obtained were in good agreement with the proposed structures.

### Synthesis and Photocleavage of s-/t-ANBP-Conjugated Single-Strand DNA

**Scheme 3:**
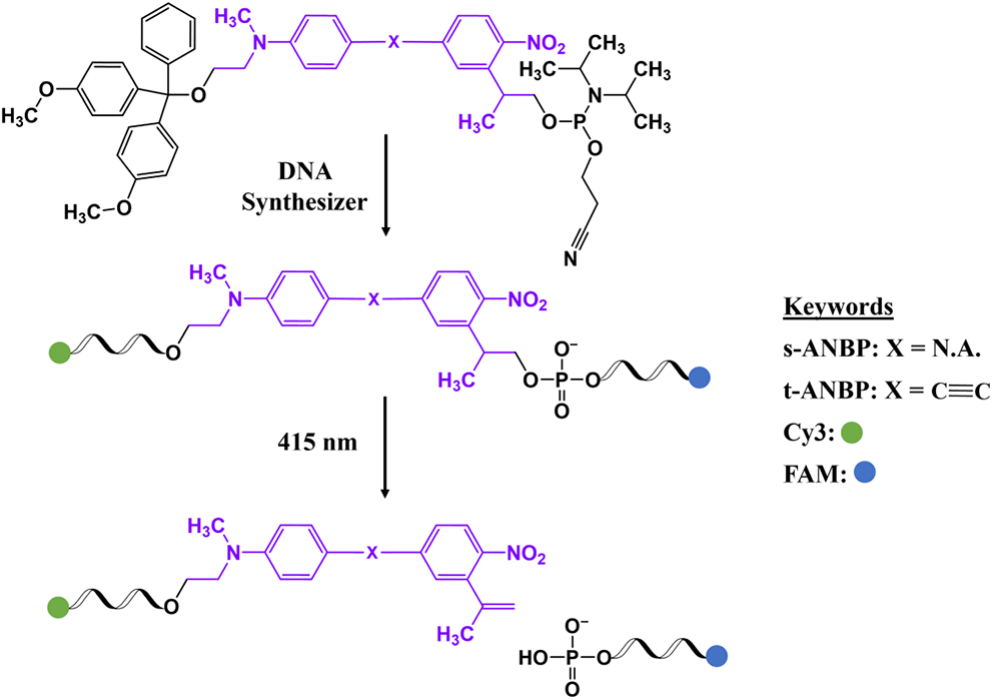
Conjugation of Photocleavable s-/t-ANBP to DNA via Solid-Phase Synthesis and their Photolysis by 415 nm Illumination

Once the pure s-ANBP and t-ANBP derivatives functionalized with DMT and phosphoramidite groups were ready, they were separately conjugated to DNA strands via solid-phase synthesis (Scheme 3). Based on our previous study,^37^ an ANBP molecule was inserted between two different lengths of DNA oligonucleotides, d15 (sequence: 5′-CTGAGACTTTAATAA-3′) and d19 (sequence: 5′-TTGAAATTCACCTGGTAGC-3′), to facilitate the characterization of fragments after photolysis (Table S1). The commercially available Cyanine 3 (Cy3) and 6-carboxyfluorescein (FAM) non-nucleosidic phosphoramidites were designed to be conjugated to the 5′ and 3′ ends, respectively, while these fluorophores were utilized for the visualization of DNA fragments and to increase the polarity difference between d15 and d19. The synthetic protocols for the photocleavable DNA oligonucleotides, including Cy3-d15-s-ANBP-d19-FAM and Cy3-d15-t-ANBP-d19-FAM, as well as the reference DNA oligonucleotides (e.g., Cy3-d15 and d19-FAM), were based on our previous study.^37^ The successful synthesis of the modified DNA oligonucleotides was confirmed by matrix-assisted laser desorption/ionization time-of-flight (MALDI-TOF) mass spectrometry (Figures S35–S38). The stability of the modified DNA oligonucleotides containing s-ANBP and t-ANBP linkers was also tested in pure water, 50 mM sodium acetate buffer at pH 4, 50 mM sodium carbonate-sodium bicarbonate buffer at pH 10, 500 mM potassium chloride solution, and at 37 °C. No degradation of the DNA oligonucleotides was observed in any of these treatments, as shown by denaturing polyacrylamide gel electrophoresis (PAGE) analysis (Figure S39). This strongly confirms that the ANBP-based photocleavable linker is stable under acidic, alkaline, and high salt content conditions, as well as at high temperatures.

**Figure 1:**
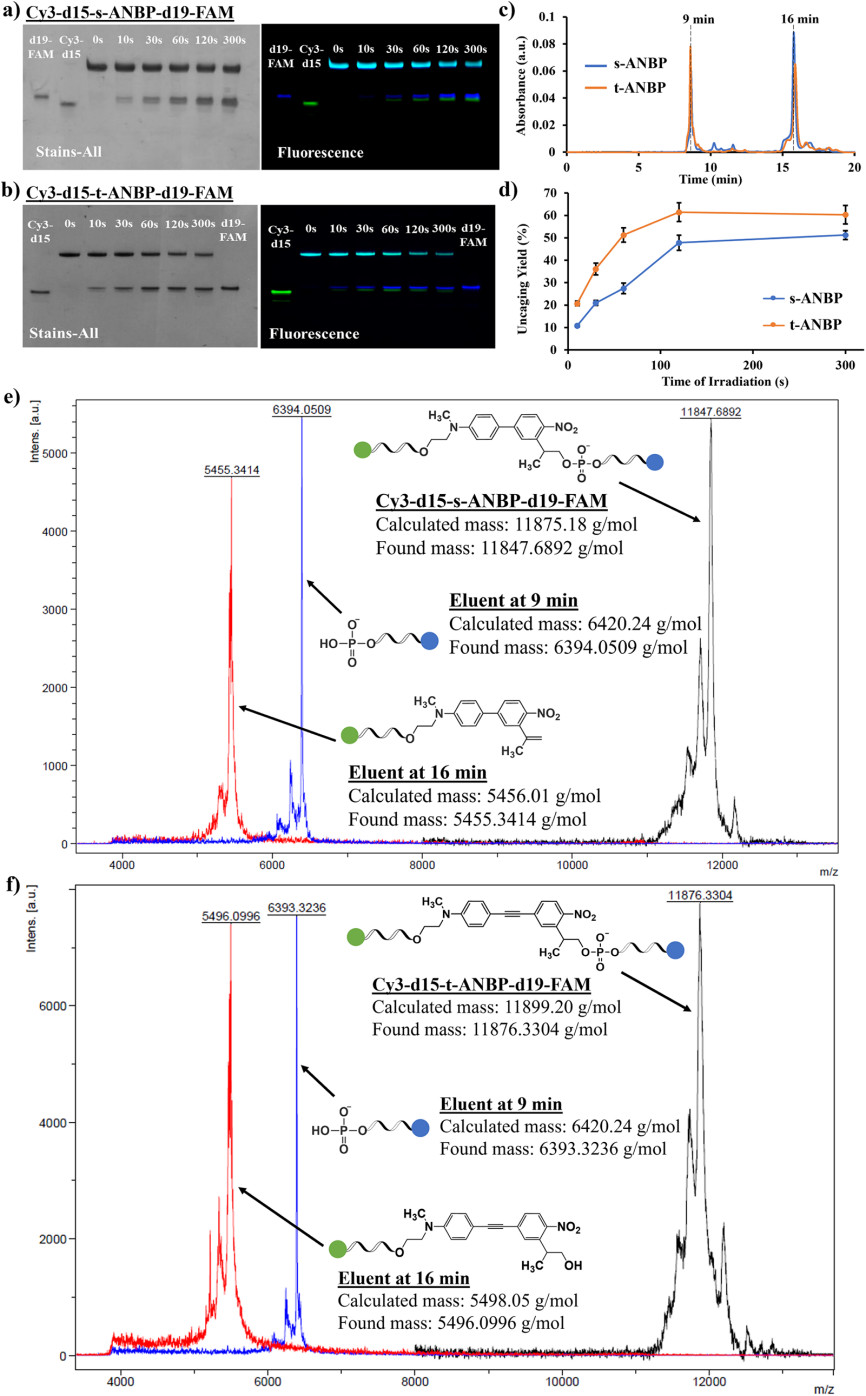
15% denaturing PAGE gel analysis of (a) Cy3-d15-s-ANBP-d19-FAM and (b) Cy3-d15-t-ANBP-d19-FAM upon 415 nm irradiation for 0, 10, 30, 60, 120, and 300 s. (c) HPLC chromatograms of s-ANBP and t-ANBP conjugated DNA oligonucleotides after 415 nm irradiation. (d) HPLC quantification of s-ANBP- and t-ANBP-conjugated DNA oligonucleotides at different time points of 415 nm irradiation. MALDI-TOF spectra of eluents collected from irradiated (e) s-ANBP and (f) t-ANBP after HPLC quantification.

Although the one-photon uncaging performance of ANBP in polyurethanes^29^ and γ-aminobutyric acid^31^ was previously reported, the photolytic efficiency of modified ANBP in DNA oligonucleotides has never been studied. To investigate this, Cy3-d15-s-ANBP-d19-FAM and Cy3-d15-t-ANBP-d19-FAM were subjected to 1X PBS at a concentration of 100 μM and irradiated with a 415 nm LED at different time points. The one-photon photolytic efficiency of Cy3-d15-s-ANBP-d19-FAM and Cy3-d15-t-ANBP-d19-FAM was examined through PAGE analysis. As shown in Figure 1a,b, the Cy3 and FAM fluorescence signals from the DNA bands corresponding to the photocleaved d15 and d19 fragments increased with increasing irradiation time for both s-/t-ANBP-conjugated oligonucleotides, while the fluorescence intensities of their parent bands decreased over time. The quantification of the photolytic efficiency of Cy3-d15-s-ANBP-d19-FAM and Cy3-d15-t-ANBP-d19-FAM was performed by using reverse-phase high-performance liquid chromatography (HPLC) analysis. The difference in polarity between Cy3 and FAM allows the d15 and d19 fragments to have different retention times on the reversed-phase HPLC column. From the HPLC chromatograms of photocleaved Cy3-d15-s-ANBP-d19-FAM and Cy3-d15-t-ANBP-d19-FAM, it was found that photocleaved d19-FAM eluted at 8 min, while Cy3-d15 eluted at 15 min (Figure 1c). The quantification of the photoreleased fragments was calculated from the peak area, which was correlated to the calibration curve of d19-FAM (Figure S40). The maximum cleavage yields of Cy3-d15-s-ANBP-d19-FAM and Cy3-d15-t-ANBP-d19-FAM were determined to be 51.2 ± 2.0% and 61.5 ± 4.2% after irradiating for 120 s, respectively (Figure 1d). The molecular masses of the two eluted fragments were confirmed by MALDI-TOF mass spectrometry (Figure 1e,f).

### Quantitative One- and Two-Photon Uncaging

One-photon uncaging quantum yields (Φ_u_) have been determined using s-ANBP- and t-ANBP-conjugated DNA oligonucleotides and DEACAS as a reference at 405 nm (Table 1 and Figures S41–S43 for photolysis curves). The two systems show low uncaging quantum yields, 0.9 and 1.1% for s-ANBP- and t-ANBP-conjugated DNA oligonucleotides, respectively. This low uncaging quantum yield is not surprising, as it is reported that the cleavage of nucleic acids is less efficient than that of other small molecules, such as neurotransmitters. In a similar way, two-photon uncaging action cross-sections (δ_u_Φ_u_) have been determined using DECAS as reference^35^ at 740 and 800 nm using a fs pulsed laser (Table 1 and Figure S44 for photolysis kinetics). The smaller conjugated system compound, s-ANBP, based on a biphenyl system, shows low two-photon-induced photolysis efficiencies, with values of δ_u_Φ_u_ under 0.5 GM at both 740 and 800 nm (0.40 and 0.39 GM, respectively). These values are 1 order of magnitude lower than those reported for neurotransmitter uncaging, mainly because of the diminution of the uncaging quantum yield of nucleic acid uncaging. However, the extended π-system molecule t-ANBP, with an ethynyl bridge between the two phenyl rings, exhibits higher δ_u_Φ_u_ values of 1.6 GM at 740 nm and 2.7 GM at 800 nm. Our results align with a previous study on 2-(*o*-nitro-phenyl)propyl caging group with a π-extended diphenylacetylene core structure.^38^

**Table 1:** One-Photon Uncaging Quantum Yield and Two-Photon Uncaging Action Cross-Section of the s-ANBP-Conjugated DNA Oligonucleotide and t-ANBP-Conjugated DNA Oligonucleotide

Compound	Φ_u_	δ_u_Φ_u_/740 nm (GM)	δ_u_Φ_u_/800 nm (GM)
s-ANBP-conjugated DNA	0.009	0.40	0.39
t-ANBP-conjugated DNA	0.011	1.6	2.7

### Design of ANBP-Conjugated DNA Duplexes and Their Photocleavage Properties

**Figure 2:**
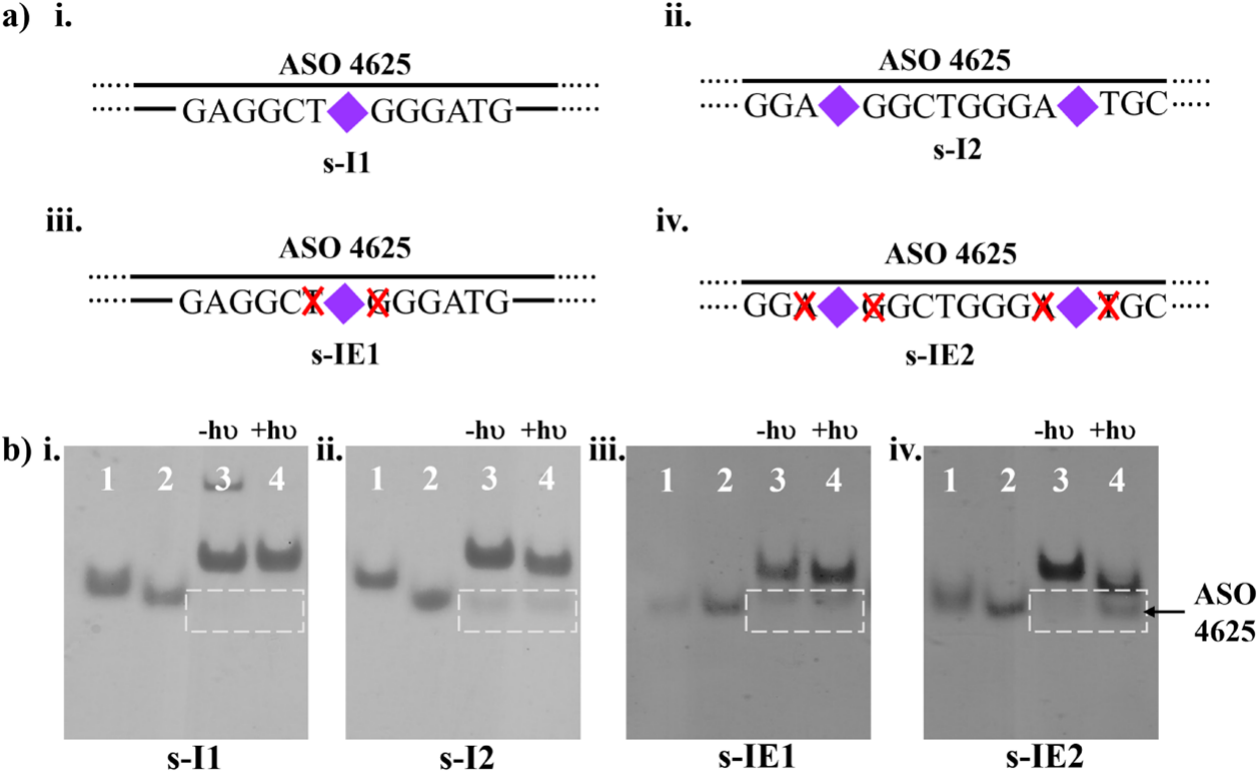
(a) Graphical illustration of ASO 4625 hybridized with complementary sense 4625 with different numbers of s-ANBP molecule insertion and base elimination. (b) 8% native PAGE analysis of duplexes shown in (a). Lane 1: modified sense 4625; lane 2: ASO 4625; lane 3: nonirradiated duplex of modified sense 4625 with ASO 4625; lane 4: duplex of modified sense 4625 with ASO 4625 irradiated with 415 nm for 5 min.

The design of photocleavable oligonucleotides, especially those conjugated with ANBP derivatives, offers a pathway to develop programmable DNA nanostructures that can be disassembled or reconfigured using light. The key challenge lies in balancing the stability of the DNA duplex with its ability to undergo efficient dissociation upon photoactivation. In this study, we initially focused on the design and evaluation of such duplexes, with an antisense oligonucleotide (ASO 4625), a 20-mer sequence, chosen as the target to be released after photocleavage. To protect ASO 4625 prior to its functional release, we designed a complementary photocleavable DNA strand (sense 4625) to form a stable duplex with ASO 4625. Two critical factors that influence the stability and dissociation efficiency of the duplex are the number of ANBP molecules inserted and the deletion of nucleotides adjacent to the ANBP. To investigate these factors, we designed and synthesized two sets of photocleavable sense 4625 strands. They were fully characterized by MALDI-TOF mass spectrometry studies (Figure S45–S46). In one set, s-I1, a single s-ANBP molecule was inserted at position 11, while in the second set, s-I2, two s-ANBP molecules were inserted at positions 7 and 16 (Figure 2a and Table S2). Both strands were hybridized with ASO 4625 and subjected to LED irradiation. Native polyacrylamide gel electrophoresis (PAGE) analysis was employed to assess the duplex stability and dissociation following light irradiation. Compared to the parent sense 4625 strand, s-I1 formed a highly stable duplex with ASO 4625 (Figure 2bi, lane 3), on the other hand, s-I2 showed a slight destabilization in duplex formation, as evidenced by the presence of unhybridized ASO 4625 (Figure 2bii, framed in lane 3). However, after 415 nm irradiation, neither duplex formed by s-I1 nor s-I2 successfully released ASO 4625, as observed in Figure 2bi,ii, lane 4. To further optimize photocleavage efficiency, we designed sense strands, such as *s*-IE1 and s-IE2, in which two nucleotides adjacent to the s-ANBP insertion sites were deleted (Figure 2aiii,iv and Figure S47–S48). Like s-I2, s-IE1 exhibited slight destabilization of the DNA duplex with ASO 4625 and poor dissociation after light irradiation at 415 nm (framed region in Figure 2biii). However, s-IE2 demonstrated improved duplex stability and, more importantly, a significant enhancement in the release of ASO 4625 after irradiation (Figure 2biv, framed in lane 4). Our results strongly indicate that merely inserting photocleavable molecules along the DNA strand is insufficient to trigger the release of ASO 4625, emphasizing that structural modifications, such as nucleotide deletions, are necessary to create mechanical strain or weaken the duplex stability in a controlled manner. The superior performance of s-IE2, with both ANBP insertions and nucleotide deletions, suggests that modifying the local DNA environment around the photocleavable sites can enhance the cleavage efficiency. The design of s-IE2 was selected for further optimization and adopted in subsequent experiments involving t-ANBP, a variant of the ANBP photocleavage molecule.

**Figure 3:**
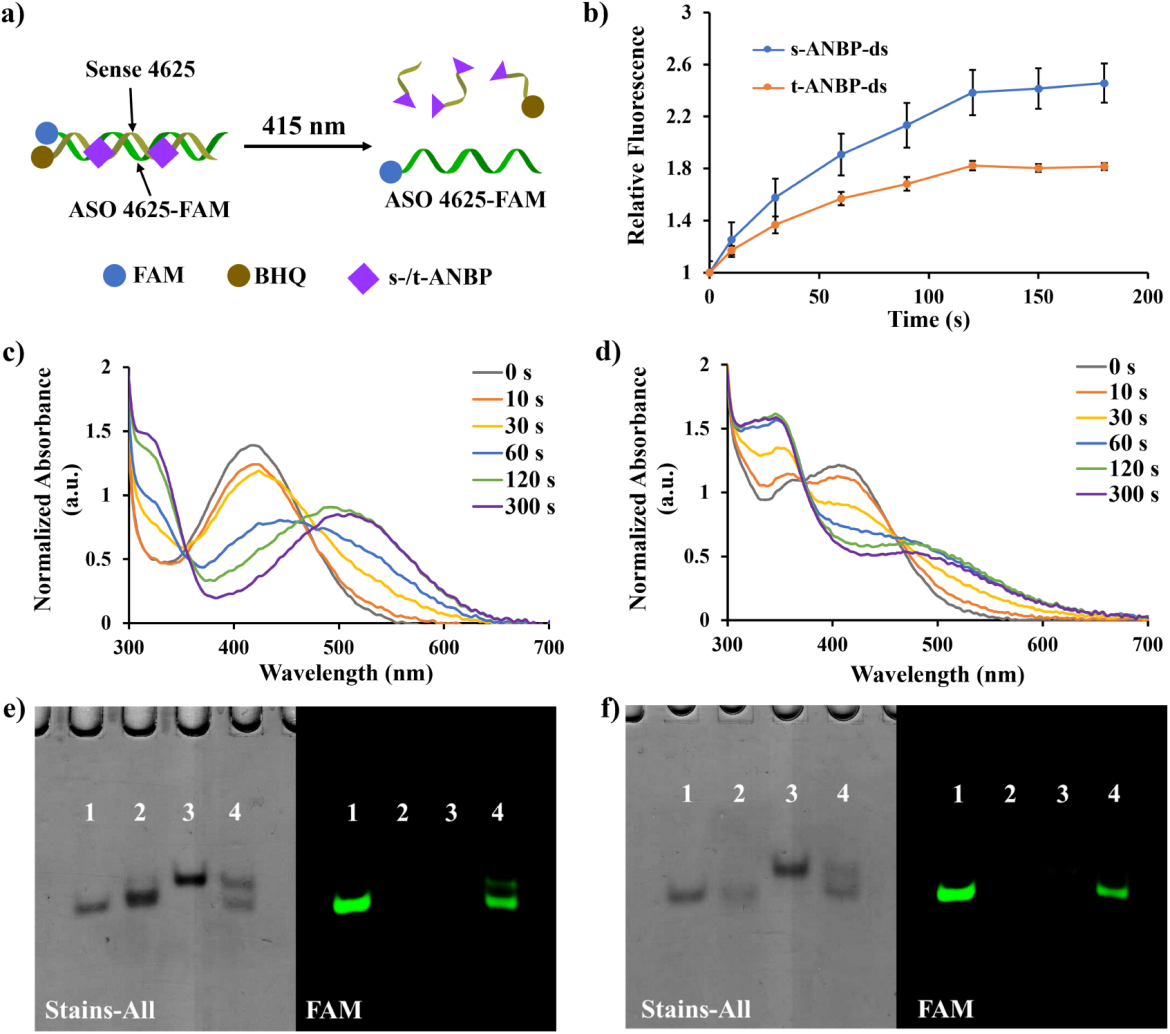
Characterization of photolysis of s-ANBP-ds and t-ANBP-ds. (a) Schematic diagram of photocleavage of s-/t-ANBP-ds labeled with FAM and BHQ. (b) Relative fluorescence measurement of s-/t-ANBP-ds labeled with FAM and BHQ over different time points of 415 nm irradiation. The fluorescence intensity of nonirradiated ANBP-ds was treated as 1. UV–vis absorption spectra of (c) s-ANBP-ds and (d) t-ANBP-ds after irradiating with 415 nm for 0, 10, 30, 60, 120, and 300 s. 8% native PAGE analysis of (e) s-ANBP-ds and (f) t-ANBP-ds assembly and photolysis. Lane 1: FAM-labeled ASO 4625; lane 2: s-IE2-BHQ/t-IE2-BHQ; lane 3: nonirradiated ANBP-ds; and lane 4: ANBP-ds were irradiated with 415 nm for 2 min. The excitation wavelength applied in the fluorescence channel was 488 nm.

To investigate and compare the photolytic efficiency of DNA duplexes conjugated with either s-ANBP or t-ANBP, we synthesized and characterized FAM-labeled ASO 4625 (ASO 4625-FAM) (Figure S49) and the corresponding BHQ-labeled IE2 strands (s-IE2-BHQ or t-IE2-BHQ) (Figure S50–S51). These were then mixed to form the respective duplexes, s-ANBP-ds and t-ANBP-ds (Figure 3a). The photolytic behavior of these duplexes was monitored using fluorescence spectroscopy, UV–vis analysis, and native PAGE. In the fluorescence study of s-ANBP-ds, a significant fluorescence enhancement was rapidly observed after irradiating for 10 s, with the fluorescence intensity continuing to increase over the irradiation period. The maximum fluorescence intensity reached a 2.4-fold increase after 2 min of 415 nm irradiation (Figure 3b). Similarly, t-ANBP-ds dissociated under 415 nm irradiation, with fluorescence enhancement reaching a plateau after 2 min. However, the fluorescence increase was only 1.8-fold, indicating a slightly lower dissociation efficiency of ASO 4625-FAM from t-ANBP-ds compared to s-ANBP-ds. This suggests that the s-ANBP duplex system may be more efficient in releasing ASO 4625 upon irradiation. UV–vis spectroscopy further supported the photocleavage of ASO 4625 from both the s-ANBP-ds and t-ANBP-ds systems. As shown in Figure 3c, the nonirradiated s-ANBP-ds (without FAM labeling) exhibited a characteristic absorption at 420 nm. Upon irradiation, this absorption decreased as a function of time, while the absorbance at 320 and 500 nm increased concurrently. Additionally, an isosbestic point was observed at 354 nm between 0 and 120 s of irradiation, suggesting a clean and defined transition between the intact duplex and the cleaved products, and further supporting the homogeneity of the photocleavage process. Our findings are consistent with previous studies, which demonstrated that the ANBP molecule undergoes a photolytic reaction following a β-elimination pathway, leading to the formation of biphenyl and 2-(*o*-nitrophenyl)propene derivatives.^31^ Similar UV absorption changes were observed in t-ANBP-ds as a function of irradiation time (Figure 3d). The phototriggered dissociation of ASO 4625 from s-ANBP-ds and t-ANBP-ds was further confirmed using native PAGE. As shown in Figure 3e,f, the successful formation of both s-ANBP-ds and t-ANBP-ds duplexes (lanes 3) was observed after mixing ASO 4625-FAM with the corresponding s-IE2-BHQ or t-IE2-BHQ. The quenching of the fluorescence indicated that the duplexes were intact, with BHQ effectively quenching the fluorescence of ASO 4625-FAM. After 2 min of 415 nm irradiation, new DNA bands were observed in both systems (Figure 3e,f, lane 4). These bands exhibited mobility comparable to that of ASO 4625-FAM, and the fluorescence intensity was strongly restored, indicating that ASO 4625-FAM had been released from the duplexes. This effect can be attributed to the photocleavage of the IE2 strand, which destabilized the duplex structures and led to the release of ASO 4625-FAM. The combined results from fluorescence restoration, UV absorption changes, and altered DNA mobility confirm the phototriggered release of protected ASO 4625 from the ANBP-modified DNA duplex structures. Notably, the s-ANBP duplex system showed slightly better dissociation efficiency compared to that of the t-ANBP system, although both systems exhibited efficient photocleavage under 415 nm irradiation. These findings provide valuable insights into the design of responsive DNA nanostructures for controlled-release applications.

### Construction and Characterization of a Photoactivatable DNA Cage Using s-ANBP for Controlled Oligonucleotide Release

**Scheme 4:**
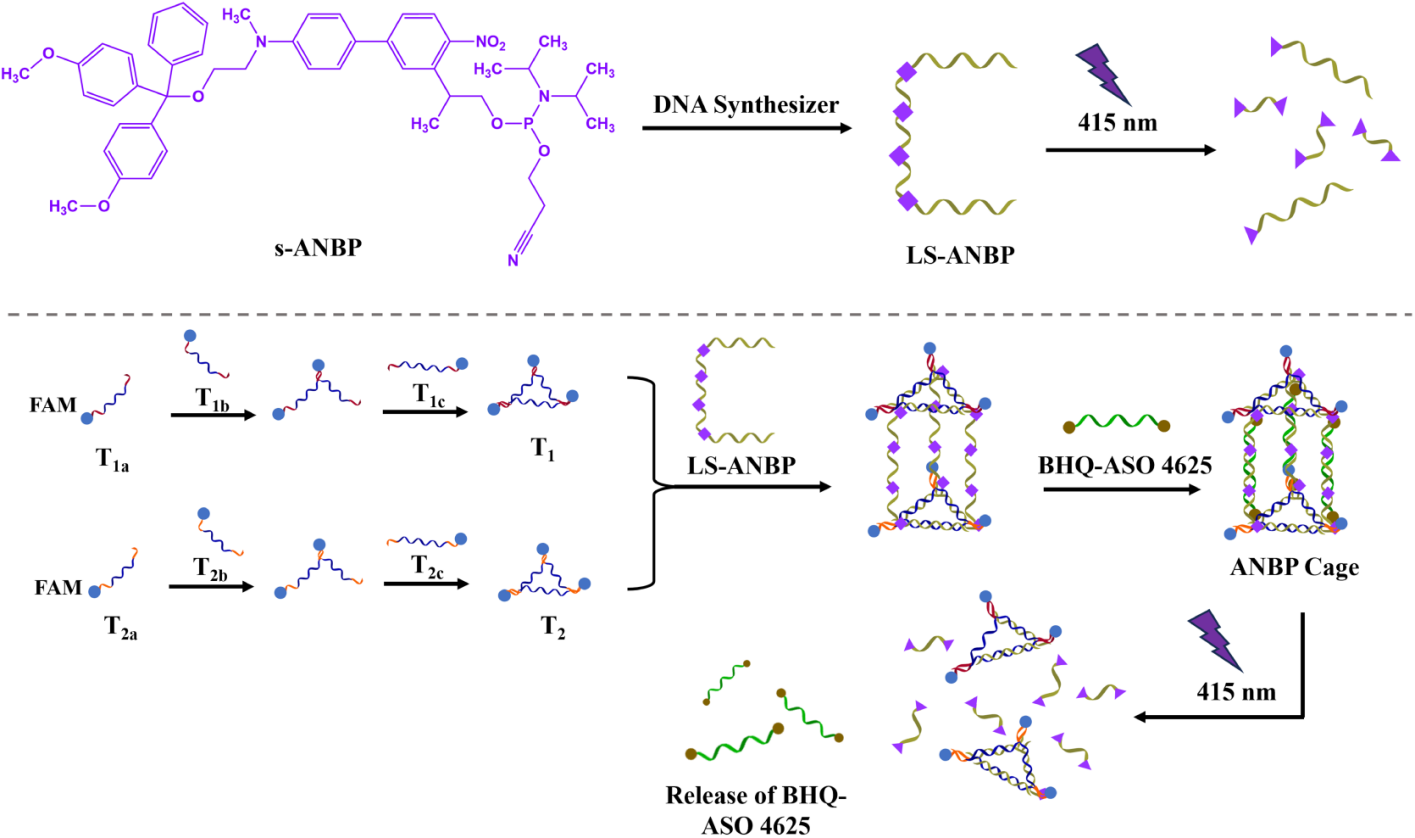
Schematic Illustration of ANBP-Cage and its Photolysis Under 415 nm Irradiation

**Figure 4:**
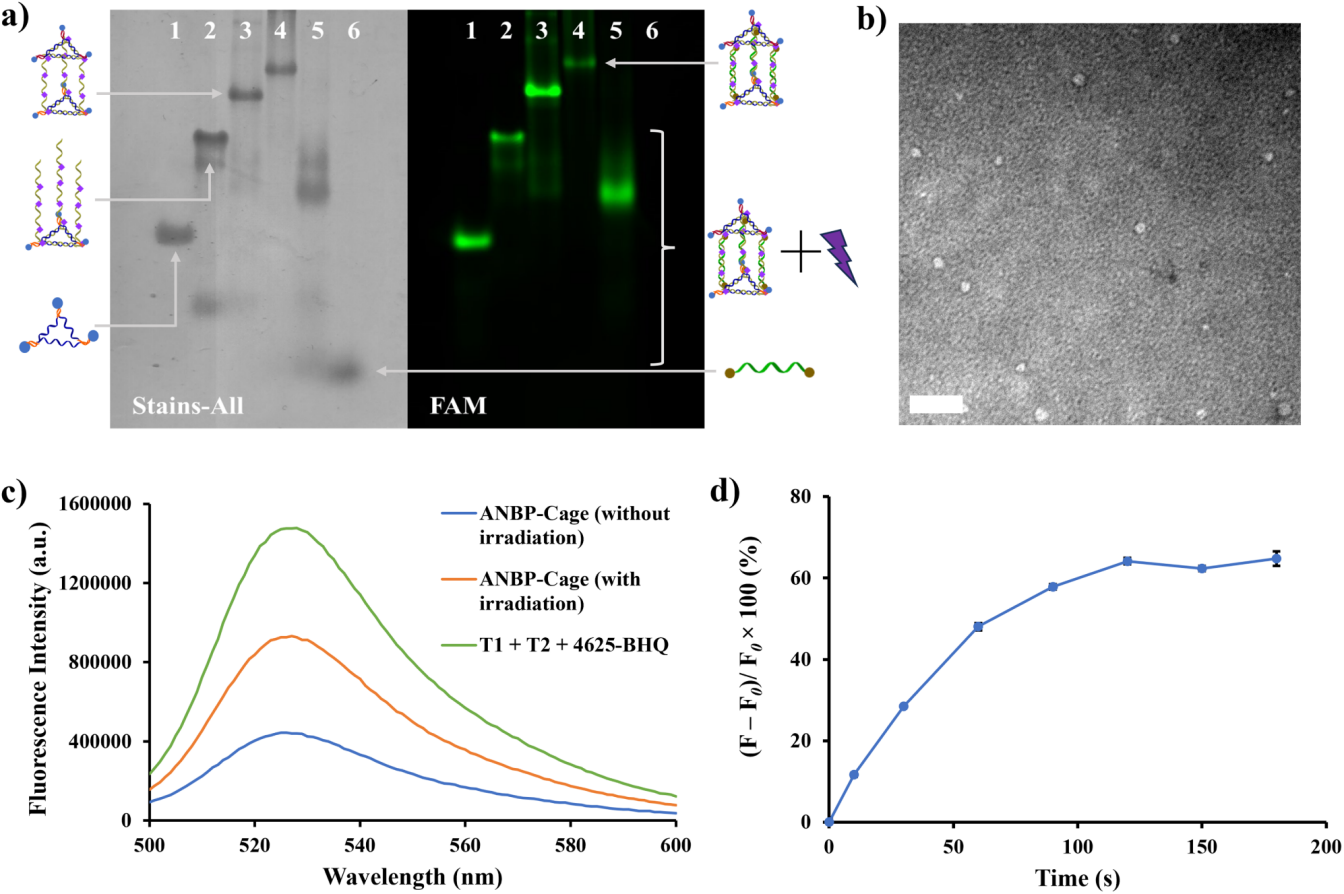
Characterization of assembly and photolysis of the ANBP-Cage. (a) 6.5% native PAGE analysis of ANBP-Cage assembly. Lane 1: FAM-labeled T2; lane 2: T2 + LS-ANBP; lane 3: T1 + T2 + LS-ANBP; lane 4: ANBP-Cage; lane 5: ANBP-Cage irradiated with 415 nm; and lane 6: ASO 4625-BHQ. The excitation wavelength applied in fluorescence channel was 488 nm. (b) TEM imaging of ANBP-Cage with negative staining. Scale bar is 50 nm. (c) Fluorescence spectra of ANBP-Cage with and without irradiated with 415 nm, ANBP-Cage without adding LS-ANBP acted as a control sample. The excitation wavelength applied was 488 nm and the emission was collected from 500 to 600 nm. (d) Percentage of fluorescence increase of ANBP-Cage over different times of 415 nm irradiation. Data are presented in mean ± standard deviation (*n* = 3).

Since the duplex system formed by the s-ANBP molecule demonstrated superior dissociation efficiency compared to that formed by the t-ANBP molecule, we selected s-ANBP as the photocleavable motif for constructing a photoactivatable DNA nanostructure. This structure would allow light-controllable release of a target oligonucleotide. For our design, we chose a DNA nanocage in the form of a triangular prism as the model system. Previous studies have shown that such DNA cages exhibit excellent properties, including efficient cellular uptake, high drug-loading capacity, and the capability to penetrate the blood-brain barrier.^39,40^ In this study, we used a self-assembly strategy similar to that of previously reported methods to construct the DNA nanocages. The assembly involved mixing the two preformed triangular-shaped DNAs (T_1_ and T_2_), three linking strands (LS-ANBP) functionalized with four ANBP molecules, and three rigidified strands (ASO 4625) as shown in Scheme 4, Table S3, and Figures S52–S59. Specifically, the rigidified strands were designed to have the same sequences as the antisense oligonucleotide 4625 and were labeled with BHQ at both terminals. The middle part of the linking strands was designed to be complementary to the sequence of ASO 4625, facilitating the formation of a stable DNA nanocage structure. To monitor the photocleavage of the ANBP-Cage, all the DNA scaffolds used to assemble T1 and T2 were labeled with FAM fluorophores at their 3′-ends. This design allowed us to track changes in fluorescence during and after irradiation. The assembly of FAM-labeled T1 and T2 was confirmed via stepwise native PAGE analysis (Figure S60). We then proceeded with the stepwise assembly of ANBP-functionalized DNA cages (ANBP-Cage) at room temperature, achieving product formation within 20 min (Figure 4a, lane 4). Upon cage formation, the fluorescence signal of the DNA band associated with ANBP-Cage was significantly diminished. This reduction in fluorescence can be attributed to the close proximity of the FAM fluorophores and the BHQ quencher, which resulted in efficient quenching of the FAM signal. After the ANBP-Cage was irradiated with 415 nm, the original DNA band of ANBP-Cage disappeared, and a new fluorescent DNA band with higher mobility was observed (Figure 4a, lane 5). Additionally, a faint band appeared near the bottom of the gel, with a mobility comparable to that of ASO 4625 (Figure 4a, lane 6). These results confirmed the phototriggered release of the target oligonucleotide, ASO 4625, from the ANBP-Cage. Size characterization of the ANBP-Cage was determined to be ∼12.2 nm by TEM imaging (Figure 4b). This observation was similar to our previous work.^39^ The fluorescence changes observed upon light irradiation of the ANBP-Cage in solution were consistent with the PAGE analysis. Initially, the FAM fluorescence emission at 520 nm from T1 and T2 was recorded before cage formation (Figure 4c, green curve). After mixing the LS-ANBP and ASO 4625 strands to assemble the DNA cages, the FAM fluorescence intensity dramatically dropped (Figure 4c, blue curve), further supporting the formation of the ANBP-Cage and the quenching of FAM by BHQ due to their close proximity. Upon irradiation at 415 nm for 2 min in TAMg buffer solution, the FAM fluorescence signal was significantly restored (Figure 4c, orange curve), indicating that the cage structure was disrupted and the ASO 4625 strand was released into the solution. However, the fluorescence signal did not fully return to its original state, suggesting that while the cage was partially or fully destroyed, some structural elements or residual interactions may still have been present, preventing complete fluorescence recovery. The fluorescence intensity increased gradually with longer irradiation times, eventually reaching a maximum increase of approximately 65% (Figure 4d). This increase in fluorescence was indicative of the progressive dissociation of the DNA nanocage and the release of the target oligonucleotide. The kinetics of the fluorescence recovery for the ANBP-Cage closely matched those observed in the s-ANBP-ds system, showing a similar magnitude of fluorescence increase and a similar time to reach a plateau.

We then investigated the stability of ANBP-Cage in biological environments to evaluate its potential for therapeutic studies. The ANBP-Cage was subjected to DMEM supplemented with 10% FBS and incubated at 37 °C for different time points. Although it was observed that there was a slight degradation of ANBP-Cage, the majority of the product remained intact after 6 h of incubation (Figure S61). This proved our ANBP-Cage exhibited a certain degree of enzymatic stability. The cellular uptake of Cy3-labeled ANBP-Cage was tested in triple-negative breast cancer MDA-MB-231 cells. MDA-MB-231 cells were incubated with 68.7 nM of Cy3-labeled ANBP-Cage for different time points, and the cells were harvested for flow cytometric analysis. The fluorescence intensity increased with the duration of incubation, as shown by the results of flow cytometry, indicating substantial cellular uptake of Cy3-labeled ANBP-Cage within 6 h of incubation (Figure S62). Confocal imaging results were consistent with those of flow cytometry, showing a clear Cy3 fluorescence signal in MDA-MB-231 cells incubated with Cy3-labeled ANBP-Cage (Figure S63). With organelle tracker staining, it was observed that the ANBP-Cage did not localize in endosomes or mitochondria but was predominantly localized in lysosomes. Trapping of ANBP-Cage in lysosomes could hinder the antisense oligonucleotides from reaching their target sites, which is unfavorable for drug delivery applications.

### Evaluation of the Antisense Function of Photoreleased ASO 4625 Using RNase H-Mediated RNA Digestion

**Figure 5:**
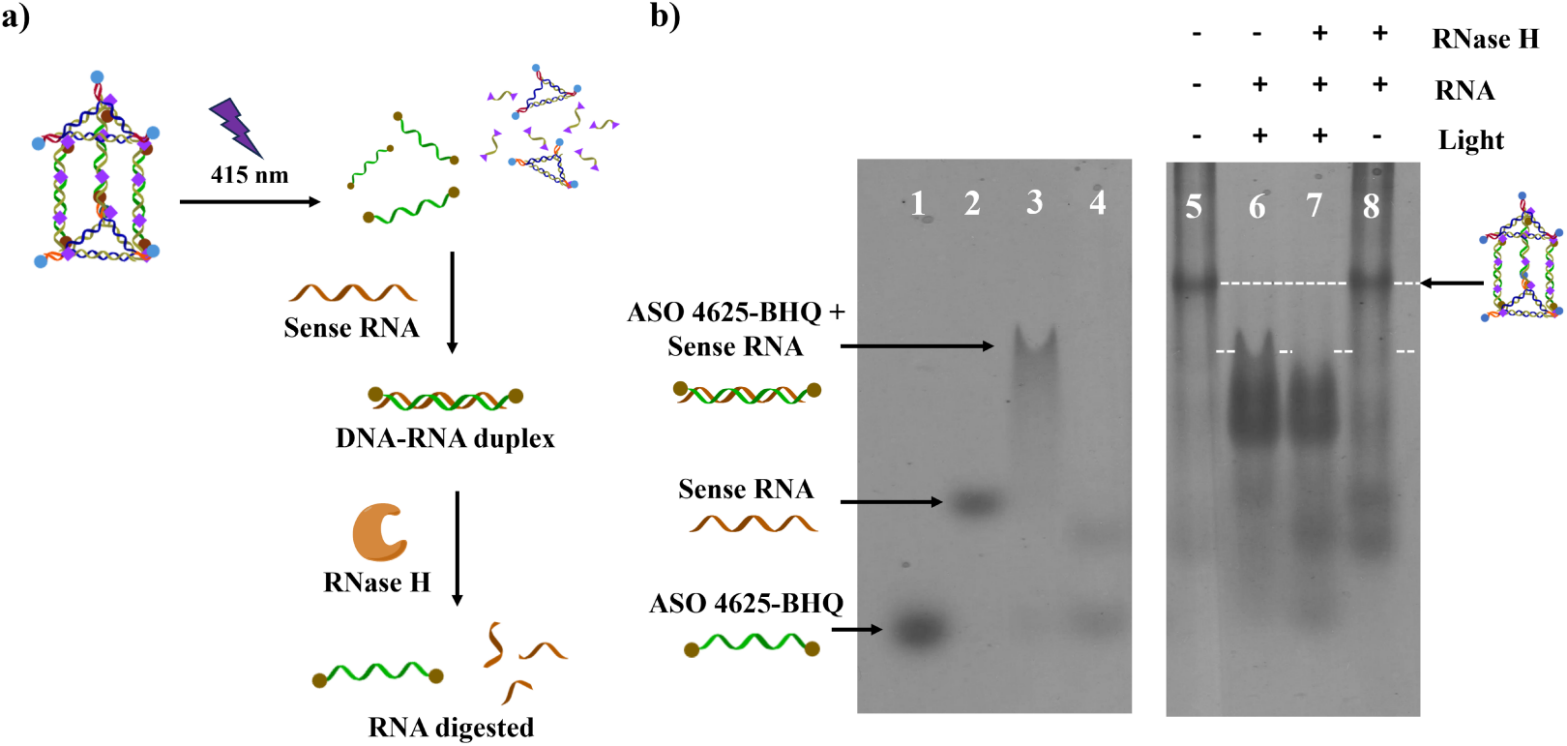
(a) Schematic diagram of *in vitro* RNA digestion test of ANBP-Cages. (b) 6.5% native PAGE analysis of *in vitro* RNA digestion by ANBP- Cage. Lane 1: ASO 4625-BHQ; lane 2: sense RNA; lane 3: DNA:RNA duplex formed by ASO 4625-BHQ and sense RNA; lane 4: DNA:RNA duplex mixed with RNase H; lane 5: nonirradiated ANBP-Cage; lane 6: 415 nm-irradiated ANBP-Cage; lane 7: 415 nm-irradiated ANBP-Cage mixed with RNase H; and lane 8: nonirradiated ANBP-Cage mixed with RNase H.

To evaluate the antisense function of the photoreleased ASO 4625, we conducted an *in vitro* RNA digestion assay using RNase H, an enzyme that specifically cleaves the RNA strand in RNA–DNA hybrids (Figure 5a). The target RNA used in this assay was a 30-mer RNA sequence complementary to the sequence of ASO 4625. RNase H cleaves the RNA strand when it forms a duplex with a complementary DNA strand, as shown in Figure 5b, lane 4. The experiment was designed to assess whether ASO 4625, once released from the ANBP-Cage, could hybridize with its complementary RNA and restore its antisense function, leading to RNA cleavage. In this experiment, ANBP-Cage was mixed with the sense RNA, followed by 415 nm irradiation. After forming the DNA–RNA hybrid, RNase H was added to incubate for 1 h, and the mixture was analyzed using 6.5% native PAGE to assess the formation of DNA–RNA hybrids and the subsequent RNA cleavage. This analysis allowed us to confirm the release of ASO 4625-BHQ from the ANBP-Cage upon irradiation and to determine whether it could function as an antisense molecule by hybridizing with the target RNA. As shown in Figure 5b, light-irradiated ANBP-Cage successfully released ASO 4625-BHQ, which then hybridized with the sense RNA to form a DNA–RNA duplex. This was evidenced by the disappearance of the sense RNA band (lane 6), indicating the formation of the hybrid. Upon the addition of RNase H, the hybridized target RNA was digested, resulting in fragmentation of the RNA strand and the release of single-stranded ASO 4625-BHQ (lane 7). This confirmed that the photoreleased ASO 4625-BHQ retained its antisense function, as it was able to hybridize with the complementary RNA and trigger RNA cleavage via RNase H. In contrast, when RNase H was added to the nonirradiated ANBP-Cage (lane 8), the band corresponding to the target RNA remained intact, indicating that ASO 4625-BHQ remained sequestered within the cage and was unable to hybridize with the target RNA. The results of the RNA digestion test confirm the successful phototriggered release of ASO 4625-BHQ from the ANBP-Cage system. Upon release, ASO 4625-BHQ hybridized with its complementary RNA sequence and restored its antisense function, leading to RNase H-mediated RNA cleavage. In contrast, without light irradiation, ASO 4625-BHQ remained trapped within the cage, and no RNA cleavage occurred, as demonstrated by the unchanged RNA bands in the nonirradiated samples. These findings suggest that the designed ANBP-Cage can act as a versatile tool for the controlled release of therapeutic agents, such as antisense oligonucleotides, in response to specific external stimuli (e.g., light). This system holds potential for drug delivery applications, where spatial and temporal control over the release of therapeutic agents is crucial for targeting specific tissues or disease sites, thereby minimizing off-target effects and enhancing therapeutic efficacy.

## Conclusion

We have synthesized novel ANBP derivatives, s-ANBP and t-ANBP, functionalized with dimethyltrityl and phosphoramidite groups to enable seamless integration into DNA backbones via solid-phase synthesis. These derivatives exhibit excellent chemical stability under acidic, alkaline, high-salt, and high-temperature conditions, making them robust for various applications. Single-stranded s-/t-ANBP-conjugated DNA oligonucleotides demonstrated reasonable one-photon photolytic efficiency under 415 nm LED irradiation, while t-ANBP, with an ethynyl-bridged extended π-system, showed enhanced two-photon absorption properties with δ_u_Φ_u_ values of 1.6 GM at 740 nm and 2.7 GM at 800 nm. In duplex forms, the stability and dissociation of complementary antisense oligonucleotides (ASOs) depended on the number of ANBP molecules incorporated and adjacent nucleotide deletions, highlighting the tunable hybridization behavior conferred by ANBP modifications. Leveraging these properties, we constructed a 3D DNA nanocage incorporating ANBP-conjugated DNA for light-triggered ASO 4625 release, which was validated by an *in vitro* RNA digestion assay using RNase H. This system offers precise spatial and temporal control over therapeutic agent release, with significant potential for photoactivated drug release and gene expression in retinal therapy.^41,42^ The two-photon uncaging property of ANBP derivatives at 740–800 nm, which falls into the biological-tissue transparency window, facilitates clinical applications. Future work on modifying the DNA nanocage with functional moieties, e.g., peptide-based polymer^43^ or cationic nucleus localization signaling (NLS) peptide,^44^ to escape from lysosomes could expand its utility for delivering other therapeutic molecules and optimizing its clinical performance.

## Supporting Information Available

Synthetic procedures of phosphoramidites of s-ANBP and t-ANBP, ^1^H, ^13^C, ^11^B, and ^31^P NMR spectra, one-photon uncaging kinetics, two-photon measurements, and mass spectra of all synthetic DNA oligonucleotides and gel electrophoretic analysis are included in the Supporting Information (PDF)

## Abbreviations

ANBP: *p*-dialkylaminonitrobiphenyl
s-ANBP: single-bond ANBP
t-ANBP: triple-bond ANBP
ASO: antisense oligonucleotide
Cy3: cyanine 3
FAM: 6-carboxyfluorescein
DNA: deoxyribonucleic acid
RNA: ribonucleic acid
HPLC: high-performance liquid chromatography
LS: linking strand
MALDI-TOF: matrix-assisted laser desorption/ionization-time-of-flight
*o*-NB: *o*-nitrobenzyl;
PAGE: polyacrylamide gel electrophoresis
TAMg: tris-acetate magnesium
UV: ultraviolet
