# Supplementary material for: Robust Photocleavable Linkers for DNA Synthesis: Enabling Visible Light-Triggered Antisense Oligonucleotide Release in 3D DNA Nanocages
Source: Biomacromolecules. 2025 Apr 24;26(5):3113–27. doi: 10.1021/acs.biomac.5c00162 (PMC12076501; doi:10.1021/acs.biomac.5c00162)
Supplement: Supplementary file 1 — bm5c00162_si_001.pdf [file bm5c00162_si_001.pdf]

Supporting Information

Robust Photocleavable Linkers for DNA Synthesis: Enabling Visible Light-Triggered Antisense Oligonucleotide Release in 3D DNA Nanocages

Hoi Man Leung,[‡a] Hau Yi Chan,[‡a] Maxime Klimezak,[b] Ling Sum Liu,[a] Pierre Karam,[c] Alexandre Specht,[b] Frédéric Bolzeb[*] and Pik Kwan Lo[a,d*]*

[a] Department of Chemistry and State Key Laboratory of Marine Pollution, City University of Hong Kong, Tat Chee Avenue, Kowloon Tong, Hong Kong, China, [b] Laboratoire de Chémo-Biologie Synthétique et Thérapeutique (CBST), Équipe Nanoparticules Intelligentes, Université de Strasbourg, CNRS, CBST UMR 7199, F-67401, Illkirch Cedex, France, [c] Department of Chemistry, AmericanUniversity of Beirut, 1107 2020 Beirut, Lebanon, [d] Key Laboratory of Biochip Technology, Biotech and Health Care, Shenzhen Research Institute of City University of Hong Kong, Shenzhen 518057, China.

KEYWORDS

DNA nanocage, ANBP, visible light-triggered, photocleavable linker, antisense oligonucleotide, control release.

EXPERIMENTAL SECTION

***N*-Methylaniline (1)**

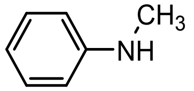

Potassium carbonate (5 g, 36.1768 mmol, 110 mol%), aniline (3 mL, 32.9217 mmol, 100 mol%) and iodomethane (2.4 mL, 38.5515 mmol, 117 mol%) were suspended in anhydrous DMF (25 mL), and stirred overnight at 80 °C. After cooling to r.t., crude mixture was diluted with EtOAc (150 mL) and washed with brine (30 mL x 5). Organic phase was collected and evaporated *in vacuo*. Residue was purified on silica gel column, isocratic eluting with DCM. Solvent was removed *in vacuo* and product was obtained as pale-yellow liquid **1** (1.3 g, 12.1318 mmol, 37%). ^{1}H NMR (400 MHz, DMSO) δ 7.10 – 7.05 (m, 2H), 6.57 – 6.46 (m, 3H), 5.58 (s, 1H), 2.65 (s, 3H); ^{13}C NMR (101 MHz, DMSO) δ 149.93, 128.86, 115.45, 111.63, 29.72; ESI-MS: m/z [M + H]$^+$ calculated for $C_7H_{10}N$: 108.1, found: 108.1.

2-(Methylphenylamino)ethanol (2)

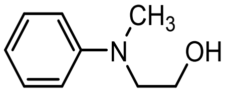

Potassium carbonate (3.9 g, 28.2179 mmol, 151 mol%), potassium iodide (1.2 g, 7.2289 mmol, 39 mol%), *N*-methylaniline **1** (2 g, 18.6644 mmol, 100 mol%) and 2-bromoethanol (2 mL, 28.2170 mmol, 151 mol%) were suspended in anhydrous DMF (20 mL), and stirred overnight at 80 °C. After cooling to r.t., crude mixture was diluted with EtOAc (120 mL) and washed with brine (30 mL x 5). Organic phase was collected and evaporated *in vacuo*. Residue was purified on silica gel column, gradient eluting with 10-30% EtOAc in DCM. Solvent was removed *in vacuo* and product was obtained as pale-yellow liquid **2** (1.4 g, 9.2587 mmol, 50%). ^{1}H NMR (400 MHz, CD_3CN) δ 7.18 (dd, *J* = 8.9, 7.2 Hz, 2H), 6.74 (dd, *J* = 8.8, 0.8 Hz, 2H), 6.63 (t, *J* = 7.2 Hz, 1H), 3.65 (dd, *J* = 10.8, 5.7 Hz, 2H), 3.42 (t, *J* = 6.1 Hz, 2H), 2.94 (s, 3H), 2.81 (t, *J* = 4.8 Hz, 1H); ^{13}C NMR (101 MHz, CD_3CN) δ 150.74, 129.98, 116.80, 113.06, 59.93, 55.47, 39.15; ESI-MS: m/z [M + H]$^+$ calculated for $C_9H_{14}NO$: 152.1, found: 152.1; m/z [M + Na]$^+$ calculated for $C_9H_{13}NNaO$: 174.1, found: 174.1.

2-((4-Bromophenyl)(methyl)amino)ethan-1-ol (3)

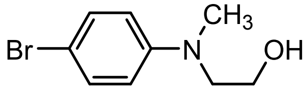

N-Bromosuccinimide (NBS) (4.71 g, 26.4636 mmol, 100 mol%) was dissolved in anhydrous MeCN (60 mL). In separate reaction flask equipped with addition funnel, 2-(methylphenylamino)ethanol **2** (4 g, 26.4535 mmol, 100 mol%) and ammonium acetate (210 mg, 2.7244 mmol, 10 mol%) were dissolved in anhydrous MeCN (20 mL). After cooling both solutions in water/ice bath, the NBS solution was transferred to addition funnel and added dropwise. Reaction mixture was warmed to r.t. and stirred overnight. Volatile materials were removed *in vacuo*. Residue was purified on silica gel column, isocratic eluting with DCM. Solvent was removed *in vacuo* and product was obtained as white solid **3** (5.01 g, 21.7727 mmol, 82%). ^{1}H NMR (400 MHz, CD_3CN) δ 7.26 (d, J = 9.2 Hz, 2H), 6.65 (d, J = 9.2 Hz, 2H), 3.63 (q, J = 5.7 Hz, 2H), 3.39 (t, J = 6.0 Hz, 2H), 2.92 (s, 3H), 2.79 (t, J = 5.4 Hz, 1H); ^{13}C NMR (101 MHz, CD_3CN) δ 149.86, 132.46, 114.81, 107.83, 59.83, 55.37, 39.20; ESI-MS: m/z $[M + H]^+$ calculated for $C_9H_{13}BrNO$: 230.0, 232.0, found: 229.8, 231.8; m/z $[M + Na]^+$ calculated for $C_9H_{12}BrNNaO$: 252.0, 254.0, found: 251.8, 254.0.

_N_-(2-(Bis(4-methoxyphenyl)(phenyl)methoxy)ethyl)-4-bromo-_N_-methylaniline (4)

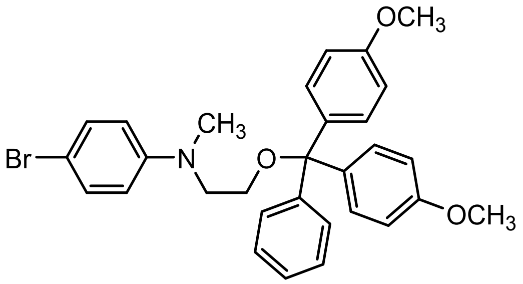

Compound **3** (1 g, 4.3458 mmol, 100 mol%), 4,4'-dimethoxytrityl chloride (2.22 g, 6.5520 mmol, 151 mol%) and 4-dimethylaminopyridine (77 mg, 0.6303 mmol, 15 mol%) were dissolved in anhydrous pyridine (20 mL), and stirred overnight at r.t.. Volatile materials were removed *in vacuo*. Residue was purified on silica gel column, isocratic eluting with 30% n-hexane in DCM containing 1% TEA. Solvent was removed *in vacuo* and product was obtained as colorless syrup **4** (2.11 g, 3.9626 mmol, 91%). ^{1}H NMR (400 MHz, DMSO) δ 7.30 – 7.18 (m, 7H), 7.15 (d, J = 8.8 Hz, 4H), 6.81 (d, J = 8.8 Hz, 4H), 6.65 (d, J = 9.0 Hz, 2H), 3.71 (s, 6H), 3.53 (t, J = 5.1 Hz, 2H), 3.10 (t, J = 5.1 Hz, 2H), 2.94 (s, 3H); ^{13}C NMR (101 MHz, DMSO) δ 157.96, 148.17, 144.86, 135.63, 131.31, 129.58, 127.71, 127.61, 126.56, 113.92, 113.05, 106.49, 85.67, 60.50, 54.98, 51.52, 38.79; ESI-MS: m/z $[M + H]^+$ calculated for $C_{30}H_{31}BrNO_3$: 532.1, 534.1, found: 532.1, 534.1; m/z $[M + Na]^+$ calculated for $C_{30}H_{30}BrNNaO_3$: 554.1, 556.1, found: 554.2, 556.1.

_N_-(2-(Bis(4-methoxyphenyl)(phenyl)methoxy)ethyl)-_N_-methyl-4-(4,4,5,5-tetramethyl-1,3,2-dioxaborolan-2-yl)aniline (5)

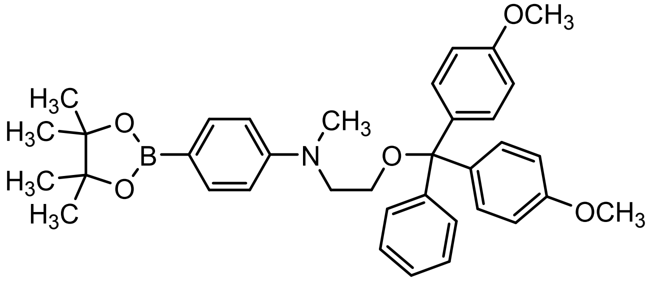

Aryl bromide **4** (3.02 g, 5.6716 mmol, 100 mol%), tetrakis(triphenylphosphine)palladium(0) (1 g, 0.8654 mmol, 15 mol%) and pinacolborane (5 mL, 34.4585 mmol, 608 mol%) were placed in round bottom pressure vessel. Anhydrous PhMe (30 mL) and anhydrous TEA (6 mL) were added to suspend the reaction mixture. The pressure vessel was sealed, and reaction mixture was heated to 115 °C for 18 h. After cooling to r.t., volatile materials were removed *in vacuo*. Residue was purified on silica gel column, gradient eluting with 30-60% DCM in PE 40-60. Solvent was removed *in vacuo* and product was obtained as yellow solid **5** (2.77 g, 4.7796 mmol, 84%). ^{1}H NMR (400 MHz, CD_2Cl_2) δ 7.58 (d, J = 8.7 Hz, 2H), 7.39 (d, J = 7.3 Hz, 2H), 7.29 – 7.19 (m, 7H), 6.78 (d, J = 8.9 Hz, 4H), 6.65 (d, J = 8.7 Hz, 2H), 3.76 (s, 6H), 3.57 (t, J = 5.7 Hz, 2H), 3.25 (t, J = 5.7 Hz, 2H), 3.05 (s, 3H), 1.31 (s, 12H); ^{11}B NMR (128 MHz, CD_2Cl_2) δ 32.16; ^{13}C NMR (101 MHz, CD_2Cl_2) δ 158.91, 151.76, 145.61, 136.55, 136.40, 130.33, 128.42, 128.12, 127.01, 113.35, 111.27, 86.64, 83.46, 61.44, 55.56, 52.57, 39.32, 25.06.

***N*-(2-(Bis(4-methoxyphenyl)(phenyl)methoxy)ethyl)-4-ethynyl-*N*-methylaniline (6)**

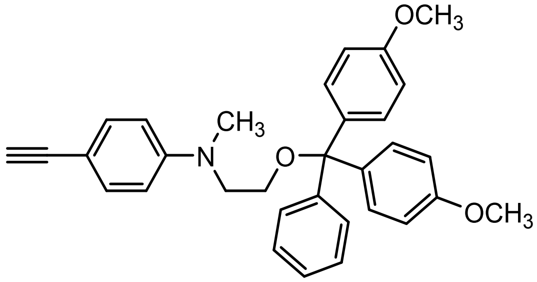

Aryl bromide **4** (1 g, 1.8780 mmol, 100 mol%), triphenylphosphine (100 mg, 0.3813 mmol, 20 mol%), copper(I) iodide (140 mg, 0.7351 mmol, 39 mol%) and bis(triphenylphosphine)palladium(II) dichloride (250 mg, 0.3562 mmol, 19 mol%) were placed in reaction flask equipped with condenser. Anhydrous TEA (30 mL) was degassed by purging nitrogen gas for 30 min, and then transferred to suspend the reaction mixture. Reaction mixture was stirred at 50 °C for 15 min, and then dimethyl ethynyl carbinol (1 mL, 10.3186 mmol, 549 mol%) was slowly added with vigorous stirring. Following the addition, reaction mixture was heated at reflux overnight. After cooling to r.t., volatile materials were removed *in vacuo*. Residue was directly loaded on silica gel column, gradient eluting with 10-30% EtOAc in n-hexane. Solvent was removed *in vacuo* and alkynyl alcohol intermediate was obtained as brown solid. The alkynyl alcohol intermediate and potassium hydroxide (370 mg, 6.5942 mmol, 351 mol%) were placed in reaction flask equipped with condenser. Anhydrous PhMe (30 mL) was added to suspend

the reaction mixture, and then heated at reflux overnight. After cooling to r.t., water (30 mL) was added and stirred for 15 min. Organic phase was collected, and aqueous phase was extracted with EtOAc (50 mL x 2). Organic phases were collected, combined, and evaporated *in vacuo*. Residue was purified on silica gel column, isocratic eluting with 25% EtOAc in PE 40-60. Solvent was removed *in vacuo* and product was obtained as brown solid **6** (670 mg, 1.4028 mmol, yield = 75%). ^{1}H NMR (400 MHz, CD_3CN) δ 7.35 (dd, *J* = 8.3, 1.3 Hz, 2H), 7.29 (d, *J* = 9.0 Hz, 2H), 7.24 – 7.19 (m, 7H), 6.79 (d, *J* = 8.9 Hz, 4H), 6.66 (d, *J* = 9.0 Hz, 2H), 3.74 (s, 6H), 3.56 (t, *J* = 5.4 Hz, 2H), 3.20 (s, 1H), 3.18 (t, *J* = 5.4 Hz, 2H), 3.00 (s, 3H); ^{13}C NMR (101 MHz, CD_3CN) δ 159.53, 150.52, 146.21, 137.07, 133.91, 130.89, 128.93, 128.71, 127.69, 113.90, 112.66, 108.91, 87.08, 85.57, 76.21, 61.95, 55.84, 52.67, 39.46.

1-Bromo-4-nitrobenzene (7)

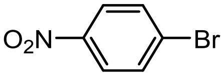

Sodium nitrate (1.7 g, 20.0024 mmol, 105 mol%) was dissolved in conc. sulfuric acid (95%, 30 mL) and cooled in water/ice bath. Bromobenzene (2 mL, 18.9924 mmol, 100 mol%) was added dropwise, and then heated at 40 °C for 1 h. After cooling in water/ice bath, water (200 mL) was slowly added with vigorous stirring. Precipitate was collected by filtration and washed with water. Crude product was purified on silica gel column, isocratic eluting with 40% DCM in n-hexane. Solvent was removed *in vacuo* and product was obtained as yellow solid **7** (1.8 g, 8.9104 mmol, yield = 47%). ^{1}H NMR (400 MHz, DMSO) δ 8.15 (d, *J* = 9.0 Hz, 2H), 7.88 (d, *J* = 9.0 Hz, 2H); ^{13}C NMR (101 MHz, DMSO) δ 146.81, 132.83, 129.34, 125.33.

***tert*-Butyl 2-(5-bromo-2-nitrophenyl)acetate (8)**

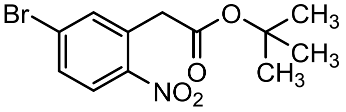

Potassium *tert*-butoxide (KO*t*Bu) (3.5 g, 31.1915 mmol, 630 mol%) was suspended in anhydrous DMF (15 mL) and cooled in water/ice bath. In separate reaction flask, 1-bromo-4-nitrobenzene **7** (1 g, 4.9502 mmol, 100 mol%) and *tert*-butyl chloroacetate (1.2 mL, 8.3904 mmol, 169 mol%) were dissolved in anhydrous DMF (10 mL), and then transferred to the KO*t*Bu solution *via* cannula. Reaction mixture was warmed to r.t. and stirred for 2 h. After cooling in water/ice bath, aqueous hydrochloric acid (5%, 15 mL) was added and stirred for 15 min. DCM (150 mL) was added and washed with brine (50 mL x 4). Organic phase was collected and evaporated *in vacuo*. Residue was purified on silica gel column, isocratic eluting with 80% DCM in n-hexane. Solvent was removed *in vacuo* and product was obtained as yellow solid **8** (1.39 g, 4.3966 mmol, yield = 89%). ^{1}H NMR (400 MHz, $CDCl_3$) δ 7.98 (d, *J* = 8.7 Hz, 1H), 7.59 (dd, *J* = 8.7, 2.0 Hz, 1H), 7.50 (d, *J* = 1.9 Hz, 1H), 3.91 (s, 2H), 1.44 (s, 9H); ^{13}C NMR (101 MHz, $CDCl_3$) δ 168.63, 147.81, 136.29, 132.49, 131.65, 128.34, 126.80, 82.35, 40.94, 28.07; ESI-MS: m/z $[M + Na]^+$

calculated for $C_{12}H_{14}BrNNaO_4$: 338.0, 340.0, found: 337.9, 339.9; m/z $[M + K]^+$ calculated for $C_{12}H_{14}BrKNO_4$: 354.0, 356.0, found: 353.9, 355.9.

tert-Butyl 2-(5-bromo-2-nitrophenyl)propanoate (9)

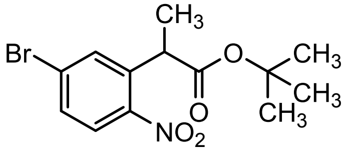

Compound **8** (2.54 g, 8.0341 mmol, 100 mol%) was dissolved in anhydrous DMF (15 mL) and cooled in water/ice bath. Potassium *tert*-butoxide solution (1 M in THF, 8.4 mL, 8.4 mmol, 105 mol%) and iodomethane (0.5 mL, 8.0316 mmol, 100 mol%) were added to reaction mixture. After warming to r.t., reaction mixture was stirred overnight. Water (20 mL) was added to the reaction mixture. Crude mixture was extracted with EtOAc (100 mL x 3). Organic phases were collected, combined, washed with brine (50 mL x 4), and then evaporated *in vacuo*. Residue was purified on silica gel column, isocratic eluting with 50% DCM in n-hexane. Solvent was removed *in vacuo* and product was obtained as yellow solid **9** (1.83 g, 5.5425 mmol, yield = 69%). ^{1}H NMR (300 MHz, $CDCl_3$) δ 7.82 (d, J = 8.7 Hz, 1H), 7.62 (d, J = 2.1 Hz, 1H), 7.54 (dd, J = 8.7, 2.1 Hz, 1H), 4.21 (q, J = 7.2 Hz, 1H), 1.57 (d, J = 7.2 Hz, 3H), 1.40 (s, 9H); ^{13}C NMR (75 MHz, $CDCl_3$) δ 171.83, 147.99, 137.79, 132.95, 131.12, 128.14, 126.46, 81.99, 42.38, 27.95, 17.49; ESI-MS: m/z $[M + Na]^+$ calculated for $C_{13}H_{16}BrNNaO_4$: 352.0, 354.0, found: 352.0, 354.0; m/z $[M + K]^+$ calculated for $C_{13}H_{16}BrKNO_4$: 368.0, 370.0, found: 367.9, 370.0.

2-(5-Bromo-2-nitrophenyl)propan-1-ol (10)

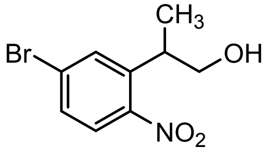

Ester **9** (1.5 g, 4.5430 mmol, 100 mol%) was dissolved in anhydrous THF (15 mL) and cooled in water/ice bath. Di*iso*butylaluminum hydride (1 M in THF, 14 mL, 14 mmol, 308 mol%) was slowly added, and then stirred in water/ice bath for 3 h. Reaction mixture was diluted with THF (50 mL), and aqueous hydrochloric acid (5%, 100 mL) was slowly added. Crude mixture was extracted with EtOAc (150 mL x 3). Organic phases were collected, combined, washed with water (50 x 2 mL), and then evaporated *in vacuo*. Residue was purified on silica gel column, isocratic eluting with 5% EtOAc in DCM. Solvent was removed *in vacuo* and product was obtained as red liquid **10** (808 mg, 3.1067 mmol, yield = 68%). ^{1}H NMR (400 MHz, $CDCl_3$) δ 7.65 (d, J = 8.6 Hz, 1H), 7.63 (d, J = 2.0 Hz, 1H), 7.49 (dd, J = 8.6, 2.0 Hz, 1H), 3.81 (dd, J = 10.8, 5.8 Hz, 1H), 3.74 (dd, J = 10.8, 7.2 Hz, 1H), 3.61 – 3.50 (m, 1H), 1.32 (d, J = 6.9 Hz, 3H); ^{13}C NMR (101 MHz, $CDCl_3$) δ 149.51, 140.67, 131.73, 130.55, 127.64, 125.86, 67.66, 36.45, 17.50.; ESI-MS: m/z $[M + Na]^+$ calculated for $C_9H_{10}BrNNaO_3$: 282.0, found: 282.0.

2-(2-Nitro-5-(4,4,5,5-tetramethyl-1,3,2-dioxaborolan-2-yl)phenyl)propan-1-ol (11)

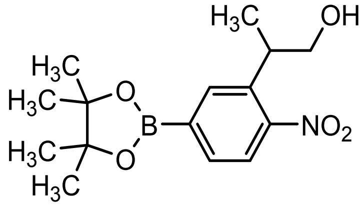

Aryl bromide **10** (1.7 g, 6.5363 mmol, 100 mol%), bis(pinacolato)diboron (2.5 g, 9.8448 mmol, 151 mol%), potassium acetate (3.9 g, 39.7391 mmol, 608 mol%) and [1,1'-bis(diphenylphosphino)ferrocene]dichloropalladium(II) (720 mg, 0.9840 mmol, 15 mol%) were suspended in anhydrous DMF (20 mL), and stirred at 80 °C for 12 h. After cooling to r.t., crude mixture was diluted with EtOAc (120 mL). Insoluble materials were removed by filtration, and filtrate was washed with brine (30 mL x 4). Organic phase was collected and evaporated *in vacuo*. Residue was purified on silica gel column, isocratic eluting with 20% EtOAc in n-hexane. Solvent was removed *in vacuo* and product was obtained as brown liquid **11** (1.3 g, 4.2324 mmol, yield = 65%). ^{1}H NMR (400 MHz, $CDCl_3$) δ 7.86 (s, 1H), 7.76 (dd, *J* = 8.0, 1.1 Hz, 1H), 7.67 (d, *J* = 8.0 Hz, 1H), 3.80 (d, *J* = 6.9 Hz, 2H), 3.44 (dd, *J* = 13.8, 6.9 Hz, 1H), 1.35 (s, 12H), 1.22 (s, 3H); ^{11}B NMR (128 MHz, $CDCl_3$) δ 30.05; ^{13}C NMR (101 MHz, $CDCl_3$) δ 152.75, 136.84, 134.51, 133.66, 123.06, 84.65, 67.97, 36.58, 24.99, 17.75; ESI-MS: m/z $[M + NH_4]^+$ calculated for $C_{15}H_{26}BN_2O_5$: 325.2, found: 325.4.

2-(4'-((2-(Bis(4-methoxyphenyl)(phenyl)methoxy)ethyl)(methyl)amino)-4-nitro-[1,1'-biphenyl]-3-yl)propan-1-ol (12)

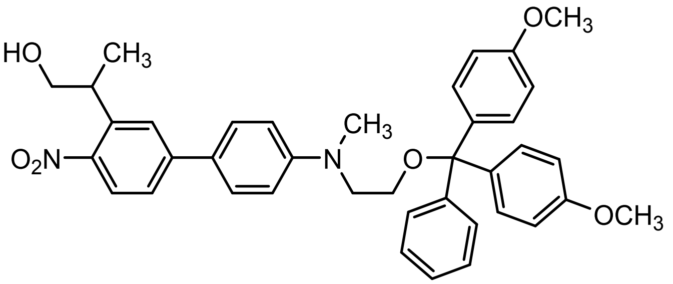

Option 1:

Aryl bromide **4** (1.865 g, 3.5025 mmol, 105 mol%), boronate ester **11** (1.024 g, 3.3338 mmol, 100 mol%), tetrakis(triphenylphosphine)palladium(0) (1.2 g, 1.0385 mmol, 31 mol%) and potassium carbonate (2.6 g, 18.8120 mmol, 564 mol%) were placed in reaction flask equipped with condenser. An immiscible solvent mixture, composed by PhMe/EtOH/H_2O (50/10/10 mL), was degassed by purging nitrogen gas for 30 min, and then transferred to reaction flask *via* cannula. Reaction mixture was heated at reflux for 16 h. After cooling to r.t., organic phase was collected,

and aqueous phase was extracted with EtOAc (100 mL x 2). Organic phases were collected, combined, and evaporated *in vacuo*. Residue was purified on silica gel column, gradient eluting with 5-10% EtOAc in DCM. Solvent was removed *in vacuo* and product was obtained as red solid **12** (1.2 g, 1.8965 mmol, yield = 57%). ^{1}H NMR (400 MHz, CD_2Cl_2) δ 7.84 (d, *J* = 8.5 Hz, 1H), 7.65 (dd, *J* = 5.0, 1.7 Hz, 1H), 7.53 (dd, *J* = 7.9, 2.6 Hz, 3H), 7.42 – 7.38 (m, 2H), 7.29 – 7.17 (m, 7H), 6.81 – 6.76 (m, 6H), 3.81 (d, *J* = 6.1 Hz, 2H), 3.74 (s, 6H), 3.68 – 3.64 (m, 1H), 3.60 (t, *J* = 5.7 Hz, 2H), 3.29 (t, *J* = 5.6 Hz, 2H), 3.08 (s, 3H), 1.37 (d, *J* = 6.9 Hz, 3H); ^{13}C NMR (101 MHz, CD_2Cl_2) δ 158.89, 150.12, 148.42, 146.18, 145.53, 139.64, 136.48, 130.33, 129.41, 128.98, 128.40, 128.34, 128.11, 127.72, 127.31, 127.03, 126.08, 125.96, 125.48, 125.15, 124.42, 113.33, 112.58, 86.68, 68.18, 61.40, 55.53, 52.74, 39.40, 36.88, 17.78.

Option 2:

Aryl bromide **10** (1.36 g, 5.2290 mmol, 126 mol%), boronate ester **5** (2.41 g, 4.1584 mmol, 100 mol%), bis(triphenylphosphine)palladium(II) dichloride (500 mg, 0.7124 mmol, 17 mol%), triphenylphosphine (350 mg, 1.3344 mmol, 32 mol%) and sodium carbonate (4.8 g, 45.2873 mmol, 1089 mol%) were placed in reaction flask equipped with condenser. An immiscible solvent mixture, composed by PhMe/EtOH/H_2O (50/10/10 mL), was degassed by purging nitrogen gas for 30 min, and then transferred to reaction flask *via* cannula. Reaction mixture was heated at reflux for 16 h. After cooling to r.t., organic phase was collected, and aqueous phase was extracted with EtOAc (100 mL x 2). Organic phases were collected, combined, and evaporated *in vacuo*. Residue was purified on silica gel column, gradient eluting with 5-10% EtOAc in DCM. Solvent was removed *in vacuo* and product was obtained as red solid **12** (1.744 g, 2.7562 mmol, yield = 66%). ^{1}H NMR (400 MHz, CD_3CN) δ 7.80 (d, *J* = 8.5 Hz, 1H), 7.70 (d, *J* = 2.0 Hz, 1H), 7.61 – 7.56 (m, 3H), 7.38 – 7.34 (m, 2H), 7.26 – 7.21 (m, 6H), 7.18 (t, *J* = 7.2 Hz, 1H), 6.83 (d, *J* = 9.0 Hz, 2H), 6.79 (d, *J* = 8.9 Hz, 4H), 3.74 – 3.65 (m, 8H), 3.62 (t, *J* = 5.4 Hz, 2H), 3.54 – 3.44 (m, 1H), 3.21 (t, *J* = 5.4 Hz, 2H), 3.05 (s, 3H), 2.85 (t, *J* = 5.8 Hz, 1H), 1.33 (d, *J* = 6.9 Hz, 3H); ^{13}C NMR (101 MHz, CD_3CN) δ 159.54, 150.86, 149.34, 146.27, 146.25, 140.55, 137.09, 130.92, 128.96, 128.91, 128.73, 127.69, 126.31, 125.99, 125.77, 124.77, 113.92, 113.35, 87.11, 67.62, 62.03, 55.83, 52.78, 39.51, 37.54, 17.95.

2-(4'-((2-(Bis(4-methoxyphenyl)(phenyl)methoxy)ethyl)(methyl)amino)-4-nitro-[1,1'-biphenyl]-3-yl)propyl (2-cyanoethyl) di*iso*propylphosphoramidite (13)

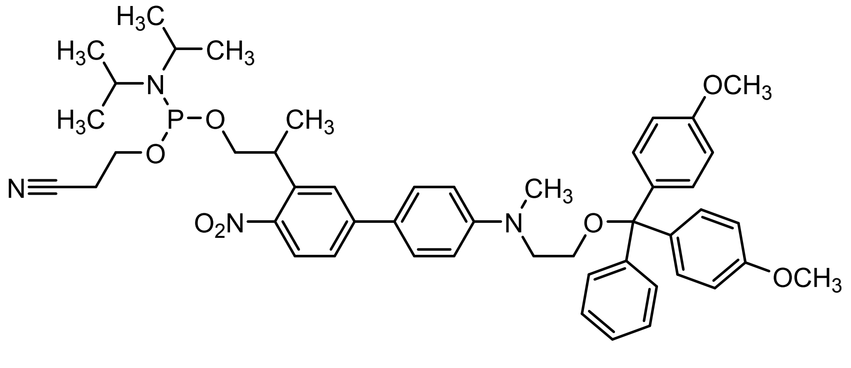

Compound **12** (525 mg, 0.8297 mmol, 100 mol%) and 5-ethylthio-1*H*-tetrazole (180 mg, 1.3828 mmol, 167 mol%) were dissolved in a solvent mixture, composed by anhydrous MeCN (6 mL) and anhydrous THF (2 mL). 2-Cyanoethyl *N,N,N',N'*-tetra*iso*propylphosphorodiamidite (0.6 mL, 1.8891 mmol, 228 mol%) was added to reaction mixture and stirred at r.t. overnight. After concentrating *in vacuo*, residue was purified on silica gel column and gradient eluting with 3-10% EtOAc in DCM containing 1% TEA. Solvent was removed *in vacuo* and product was obtained as orange solid **13** (420 mg, 0.5042 mmol, yield = 61%). ^{1}H NMR (400 MHz, CD_2Cl_2) δ 7.83 (dd, *J* = 8.5, 2.6 Hz, 1H), 7.66 (dd, *J* = 4.7, 1.9 Hz, 1H), 7.52 (dd, *J* = 9.5, 3.0 Hz, 2H), 7.38 (d, *J* = 7.4 Hz, 2H), 7.29 – 7.17 (m, 8H), 6.80 – 6.75 (m, 6H), 3.85 – 3.79 (m, 2H), 3.77 – 3.69 (m, 9H), 3.60 (t, *J* = 5.7 Hz, 2H), 3.53 – 3.45 (m, 2H), 3.28 (t, *J* = 5.6 Hz, 2H), 3.07 (s, 3H), 2.53 – 2.47 (m, 2H), 1.40 (d, *J* = 6.7 Hz, 3H), 1.14 – 1.03 (m, 12H); ^{31}P NMR (162 MHz, CD_2Cl_2) δ 147.86.

2-(5-Ethynyl-2-nitrophenyl)propan-1-ol (14)

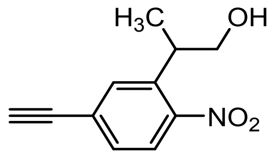

Aryl bromide **10** (1.856 g, 7.1361 mmol, 100 mol%) and tetrakis(triphenylphosphine)palladium(0) (1.9 g, 1.6442 mmol, 23 mol%) were placed in round bottom pressure vessel. Anhydrous PhMe (30 mL) was added to dissolve the reaction mixture. After stirring 15 min at r.t., tributyl(trimethylsilylethynyl)tin (5 g, 12.9109 mmol, 181 mol%) was slowly added. The pressure vessel was sealed, and reaction mixture was stirred at 110 °C for 18 h. After cooling to r.t., volatile materials were removed *in vacuo*. Residue was purified on silica gel column, isocratic eluting with 5% EtOAc in DCM. Solvent was removed *in vacuo* and silylated intermediate was obtained as orange solid. The silylated intermediate was dissolved in anhydrous THF (15 mL). After cooling in water/ice bath, tetra-*n*-butylammonium fluoride (1 M in THF, 18 mL, 18 mmol, 252 mol%) was slowly added. Reaction mixture was warmed to r.t. and stirred for 1 h. Water (30 mL) was added to the reaction mixture and stirred for 20 min. Crude mixture was extracted with EtOAc (100 mL x 3). Organic phases were collected, combined, and evaporated *in vacuo*. Residue was purified on silica gel column, gradient eluting with 5-10% EtOAc in DCM. Solvent was removed *in vacuo* and product was obtained as red liquid **14** (980 mg, 4.7756 mmol, yield = 67%). ^{1}H NMR (300 MHz, CD_2Cl_2) δ 7.69 (d, *J* = 8.4 Hz, 1H), 7.59 (d, *J* = 1.7 Hz, 1H), 7.43 (dd, *J* = 8.4, 1.7 Hz, 1H), 3.70 – 3.64 (m, 2H), 3.43 (dd, *J* = 13.5, 6.8 Hz, 1H), 3.37 (s, 1H), 1.98 (s, 1H), 1.27 (d, *J* = 6.9 Hz, 3H); ^{13}C NMR (75 MHz, CD_2Cl_2) δ 150.51, 139.17, 132.56, 130.92, 127.03, 124.53, 82.10, 81.17, 67.46, 36.59, 17.54; ESI-MS: m/z $[M + H]^+$ calculated for $C_{11}H_{12}NO_3$: 206.1, found: 206.3; m/z $[M + Na]^+$ calculated for $C_{11}H_{11}NNaO_3$: 228.1, found: 228.0.

2-(5-((4-((2-(Bis(4-methoxyphenyl)(phenyl)methoxy)ethyl)(methyl)amino)phenyl)ethynyl)-2-nitrophenyl)propan-1-ol (15)

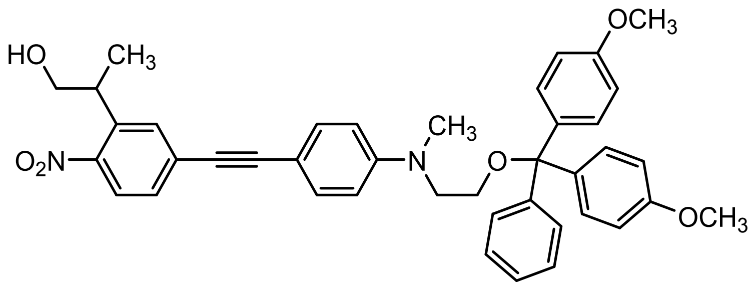

Option 1:

Aryl bromide **4** (1.26 g, 2.3663 mmol, 100 mol%), triphenylphosphine (190 mg, 0.7244 mmol, 31 mol%), copper(I) iodide (230 mg, 1.2077 mmol, 51 mol%), bis(triphenylphosphine)palladium(II) dichloride (530 mg, 0.7551 mmol, 32 mol%) and terminal alkyne **14** (570 mg, 2.7776 mmol, 117 mol%) were placed in round bottom pressure vessel. Anhydrous TEA (30 mL) was added to suspend the reaction mixture. The pressure vessel was sealed, and reaction mixture was heated to 90 °C for 18 h. After cooling to r.t., volatile materials were removed *in vacuo*. Residue was purified on silica gel column, gradient eluting with 5-10% EtOAc in DCM. Solvent was removed *in vacuo* and product was obtained as red solid **15** (715 mg, 1.0886 mmol, yield = 46%). ^{1}H NMR (400 MHz, CD_2Cl_2) δ 7.76 (d, *J* = 8.4 Hz, 1H), 7.61 (d, *J* = 1.7 Hz, 1H), 7.46 – 7.39 (m, 5H), 7.30 – 7.20 (m, 7H), 6.80 (d, *J* = 9.0 Hz, 4H), 6.68 (d, *J* = 9.0 Hz, 2H), 3.81 – 3.75 (m, 8H), 3.62 – 3.52 (m, 3H), 3.29 (t, *J* = 5.6 Hz, 2H), 3.08 (s, 3H), 1.83 (s, 1H), 1.34 (d, *J* = 6.9 Hz, 3H); ^{13}C NMR (101 MHz, CD_2Cl_2) δ 158.90, 150.05, 149.17, 145.46, 139.27, 136.43, 133.42, 131.13, 130.31, 129.68, 128.39, 128.12, 127.05, 124.88, 113.34, 111.99, 108.46, 95.69, 86.70, 86.51, 67.90, 61.34, 55.55, 52.64, 39.43, 36.68, 17.62.

Option 2:

Aryl bromide **10** (900 mg, 3.4604 mmol, 100 mol%), triphenylphosphine (270 mg, 1.0294 mmol, 30 mol%), copper(I) iodide (330 mg, 1.7327 mmol, 50 mol%), bis(triphenylphosphine)palladium(II) dichloride (920 mg, 1.3107 mmol, 38 mol%) and terminal alkyne **6** (2.48 g, 5.1926 mmol, 150 mol%) were placed in round bottom pressure vessel. Anhydrous TEA (40 mL) was added to suspend the reaction mixture. The pressure vessel was sealed, and reaction mixture was heated to 90 °C for 24 h. After cooling to r.t., volatile materials were removed *in vacuo*. Residue was purified on silica gel column, gradient eluting with 5-10% EtOAc in DCM. Solvent was removed *in vacuo* and product was obtained as red solid **15** (910 mg, 1.3855 mmol, yield = 40%). ^{1}H NMR (400 MHz, Acetone) δ 7.78 (d, *J* = 8.4 Hz, 1H), 7.70 (d, *J* = 1.6 Hz, 1H), 7.48 (dd, *J* = 8.4, 1.7 Hz, 1H), 7.44 – 7.38 (m, 4H), 7.31 – 7.18 (m, 7H), 6.82 (d, *J* = 8.9 Hz, 4H), 6.77 (d, *J* = 8.9 Hz, 2H), 3.75 (s, 8H), 3.64 (t, *J* = 5.4 Hz, 2H), 3.46 (dd, *J* = 13.4, 6.8 Hz, 1H), 3.29 (t, *J* = 5.4 Hz, 2H), 3.09 (s, 3H), 2.93 (s, 1H), 1.34 (d, *J* = 6.9 Hz, 3H); ^{13}C NMR (101 MHz, Acetone) δ 159.45, 150.66, 150.13, 146.13, 140.16, 136.90, 133.80, 131.67, 130.81, 130.01, 129.60, 128.87, 128.49, 127.42, 125.04, 113.76, 112.62, 108.95, 95.70, 87.08, 86.91, 67.29, 61.83, 55.43, 52.67, 39.44, 37.34, 17.86.

2-(5-((4-((2-(Bis(4-methoxyphenyl)(phenyl)methoxy)ethyl)(methyl)amino)phenyl)ethynyl)-

2-nitrophenyl)propyl (2-cyanoethyl) di*iso*propylphosphoramidite (16)

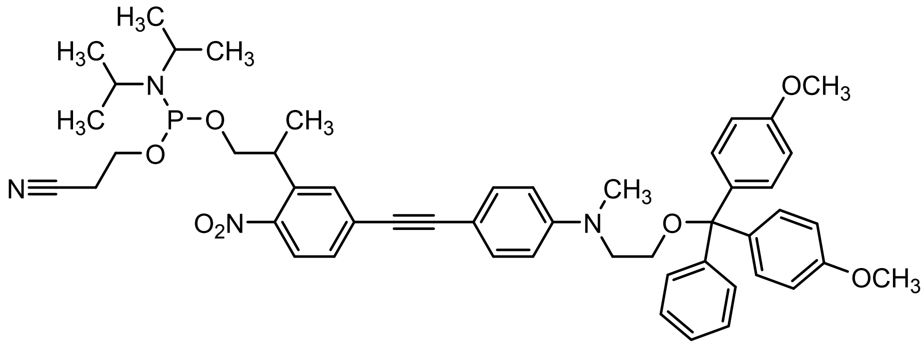

Compound **15** (700 mg, 1.0658 mmol, 100 mol%) and 5-ethylthio-1*H*-tetrazole (230 mg, 1.7669 mmol, 166 mol%) were dissolved in a solvent mixture, composed by anhydrous MeCN (7 mL) and anhydrous THF (3 mL). 2-Cyanoethyl *N,N,N',N'*-tetra*iso*propylphosphorodiamidite (0.7 mL, 2.2040 mmol, 207 mol%) was added to reaction mixture and stirred at r.t. overnight. After concentrating *in vacuo*, residue was purified on silica gel column and gradient eluting with 3-10% EtOAc in DCM containing 1% TEA. Solvent was removed *in vacuo* and product was obtained as orange solid **16** (691 mg, 0.8063 mmol, yield = 76%). ^{1}H NMR (400 MHz, CD_2Cl_2) δ 7.74 (dd, *J* = 6.3, 2.1 Hz, 1H), 7.68 – 7.64 (m, 2H), 7.54 – 7.47 (m, 2H), 7.33 – 7.26 (m, 9H), 6.84 – 6.80 (m, 6H), 3.80 – 3.76 (m, 11H), 3.61 – 3.56 (m, 4H), 3.31 (t, *J* = 5.6 Hz, 2H), 3.09 (s, 3H), 2.61 (dd, *J* = 6.3, 1.9 Hz, 2H), 1.34 – 1.29 (m, 3H), 1.16 – 1.10 (m, 12H); ^{31}P NMR (162 MHz, CD_2Cl_2) δ 147.66.

ADDITIONAL DATA

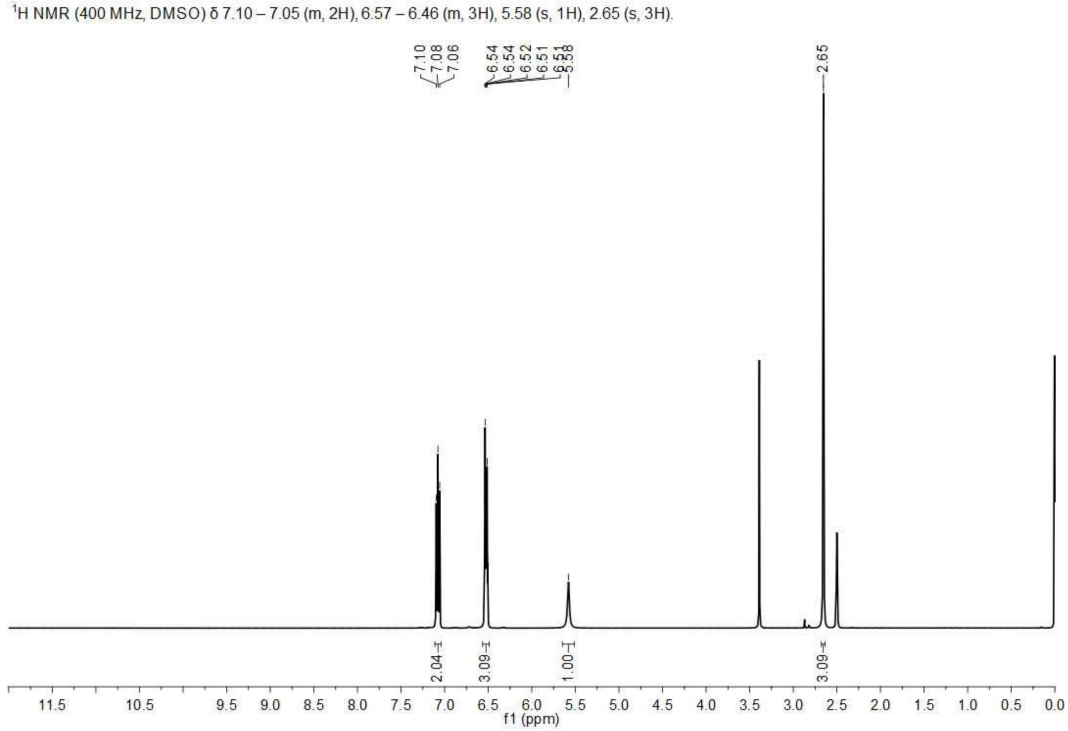

Figure S1. ^{1}H NMR spectrum of compound **1**.

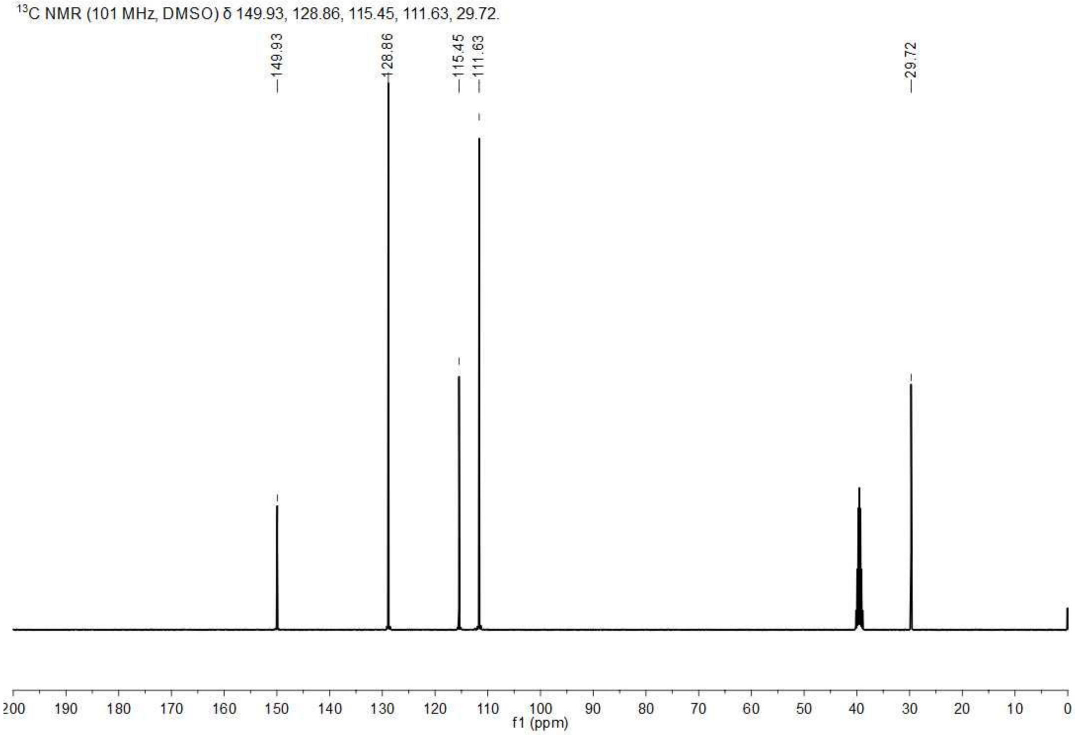

Figure S2. ^{13}C NMR spectrum of compound **1**.

^{1}H NMR (400 MHz, CD_3CN) δ 7.18 (dd, J = 8.9, 7.2 Hz, 2H), 6.74 (dd, J = 8.8, 0.8 Hz, 2H), 6.63 (t, J = 7.2 Hz, 1H), 3.65 (dd, J = 10.8, 5.7 Hz, 2H), 3.42 (t, J = 6.1 Hz, 2H), 2.94 (s, 3H), 2.81 (t, J = 4.8 Hz, 1H).

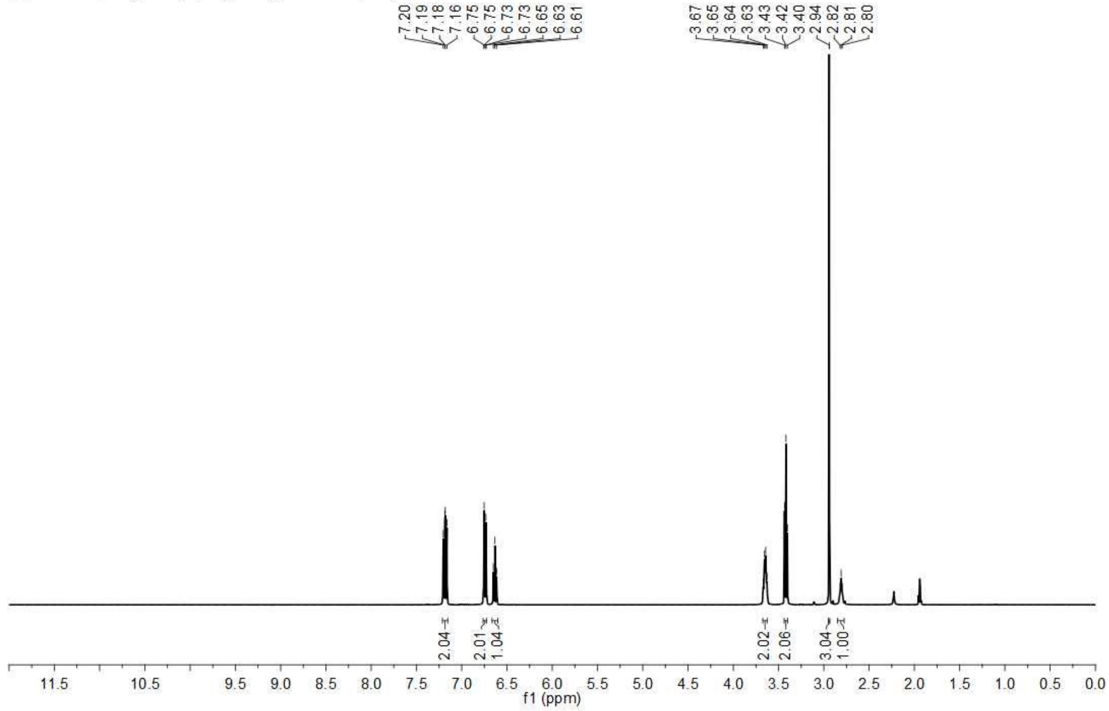

Figure S3. ^{1}H NMR spectrum of compound **2**.

^{13}C NMR (101 MHz, CD_3CN) δ 150.74, 129.98, 116.80, 113.06, 59.93, 55.47, 39.15.

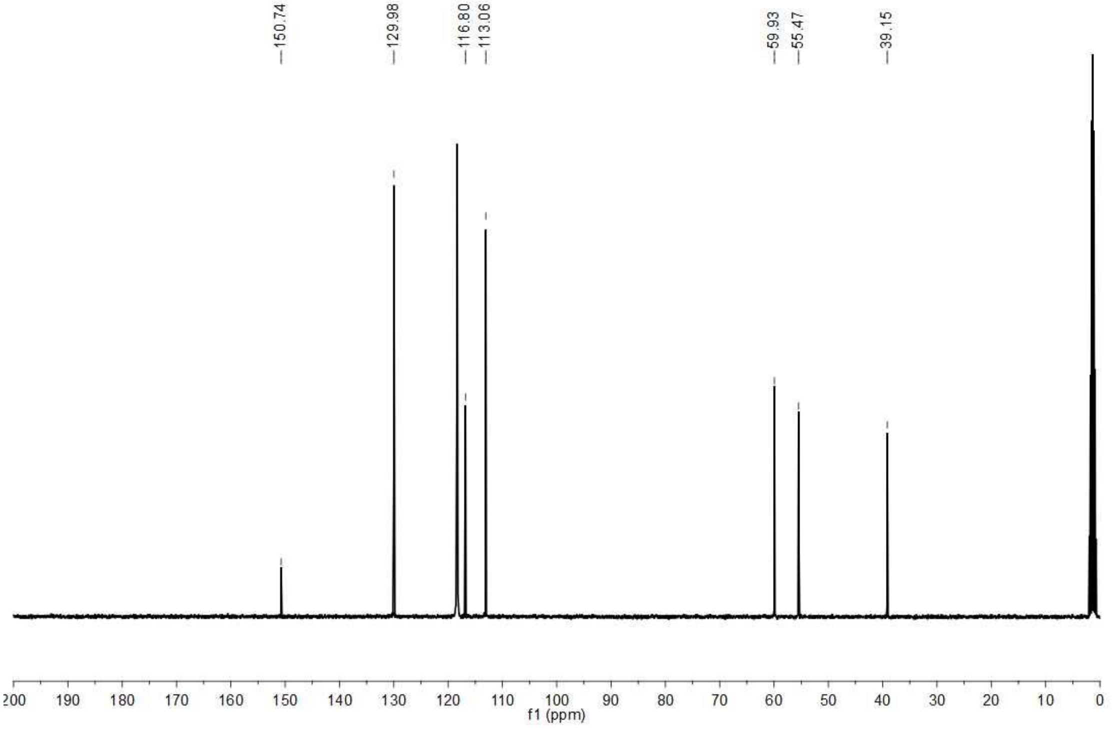

Figure S4. ^{13}C NMR spectrum of compound **2**.

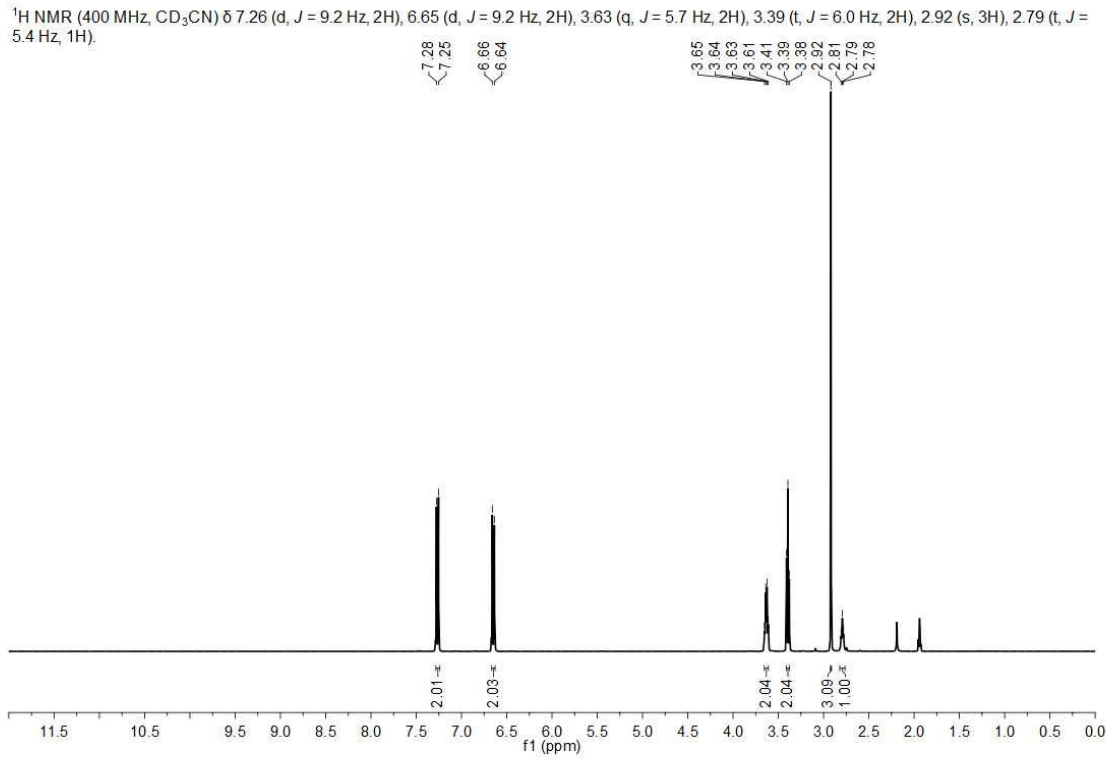

Figure S5. ^{1}H NMR spectrum of compound **3**.

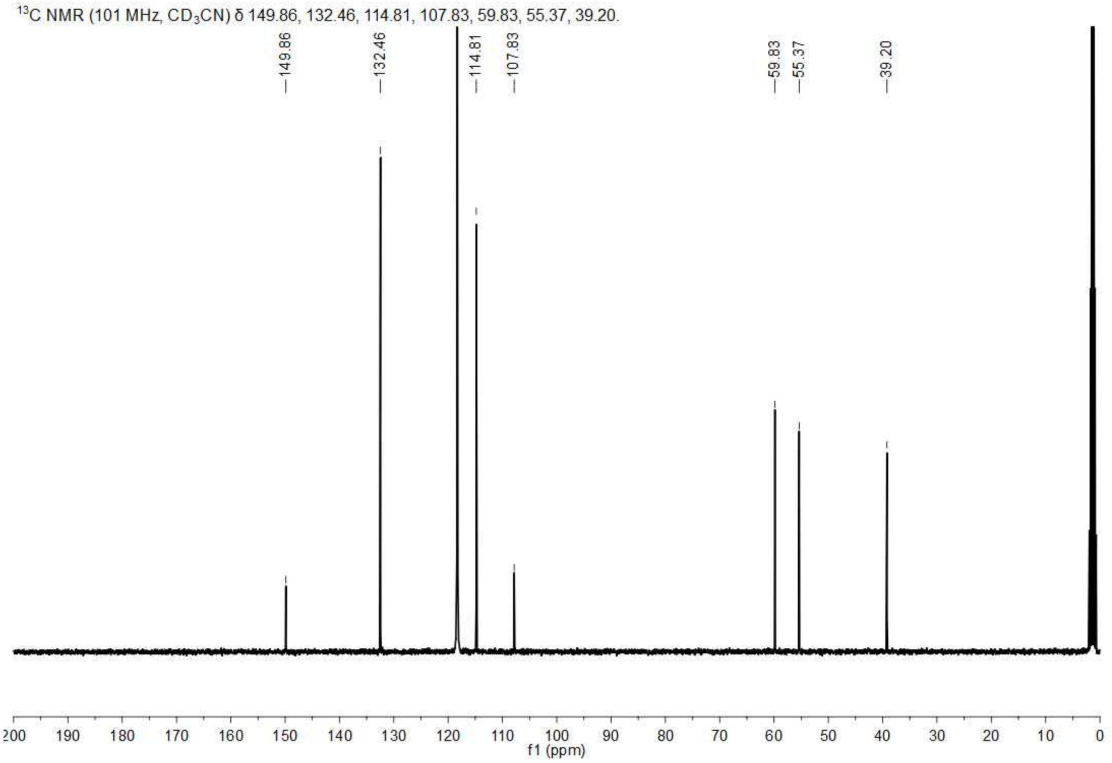

Figure S6. ^{13}C NMR spectrum of compound **3**.

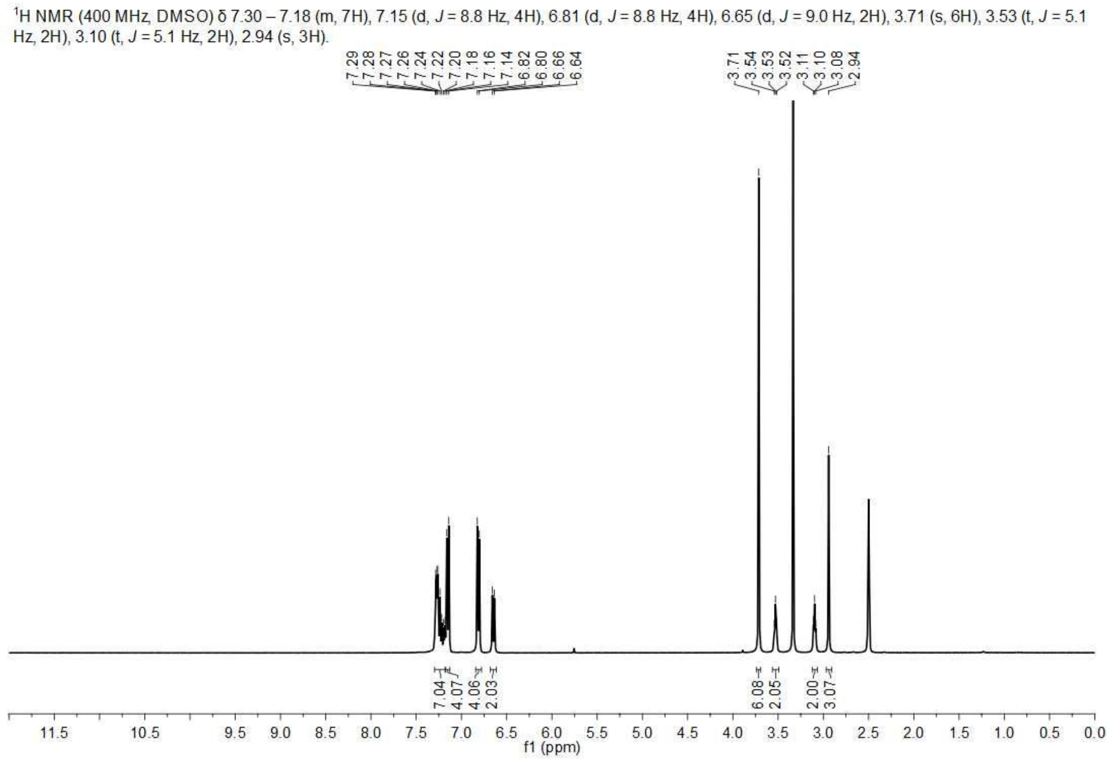

Figure S7. ^{1}H NMR spectrum of compound **4**.

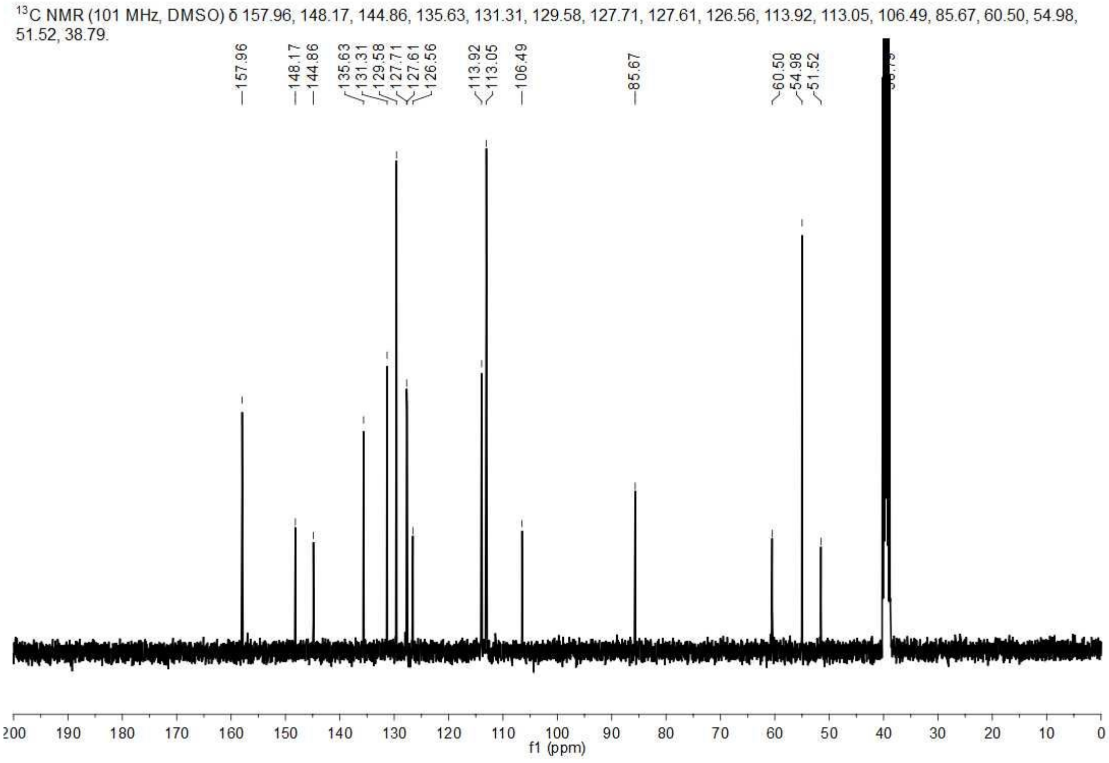

Figure S8. ^{13}C NMR spectrum of compound **4**.

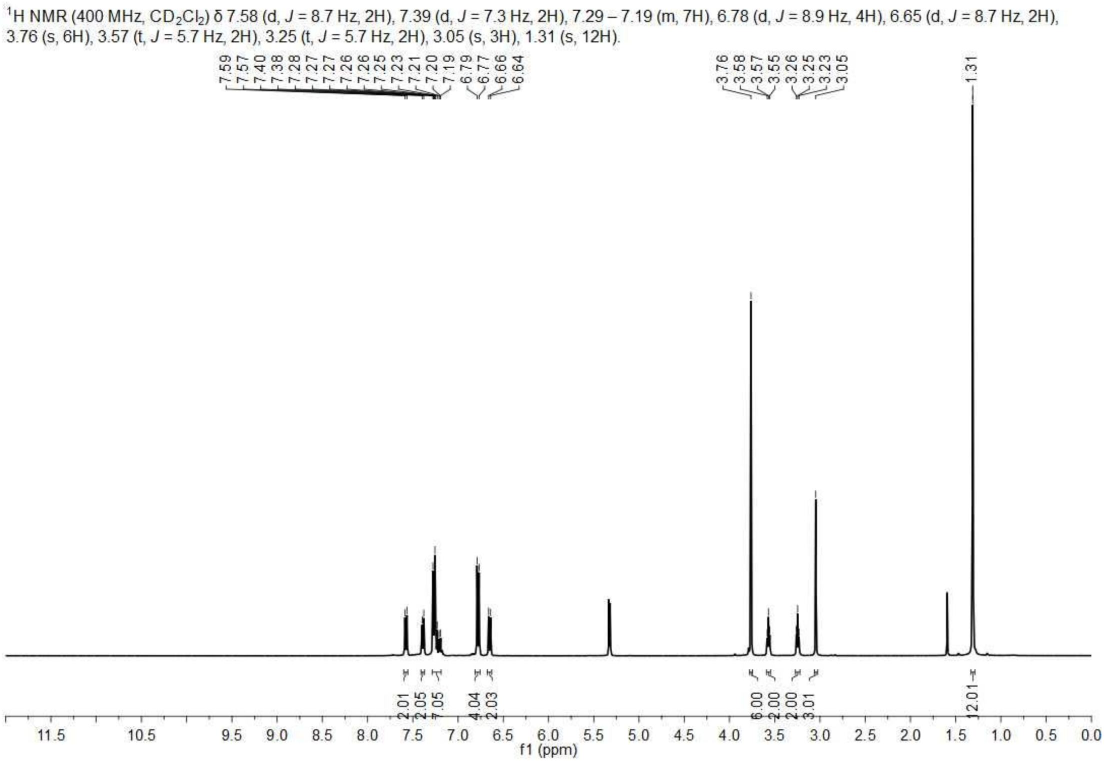

Figure S9. ^{1}H NMR spectrum of compound **5**.

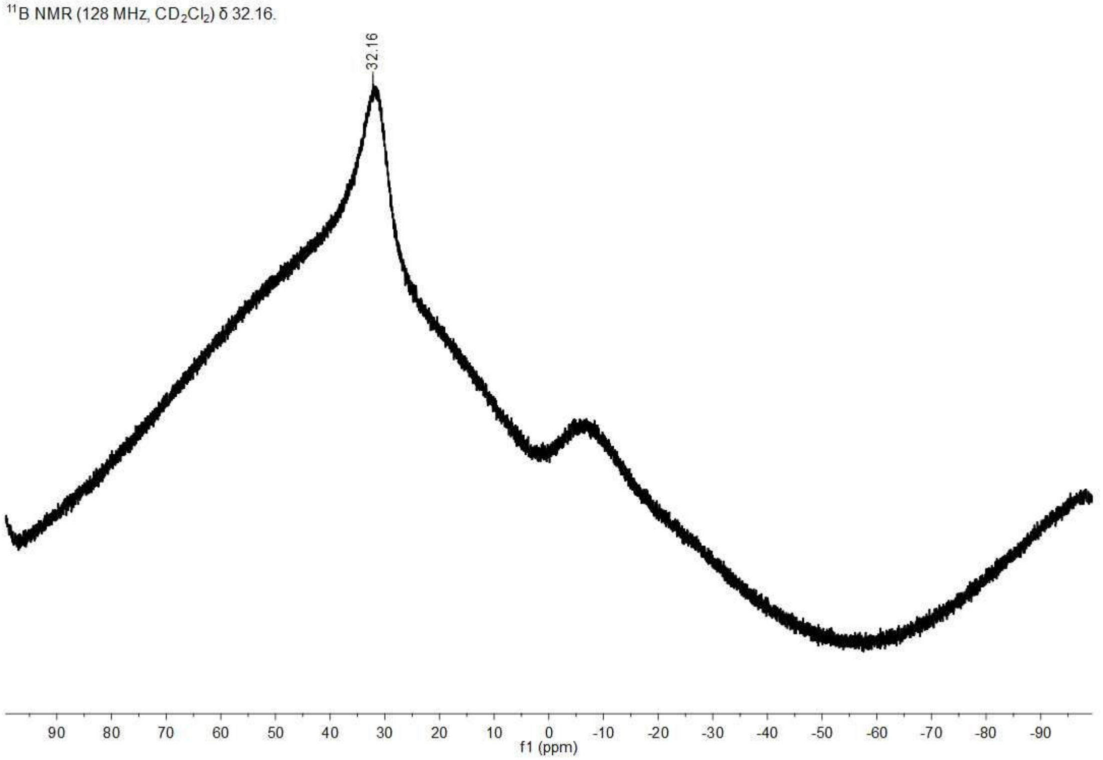

Figure S10. ^{11}B NMR spectrum of compound **5**.

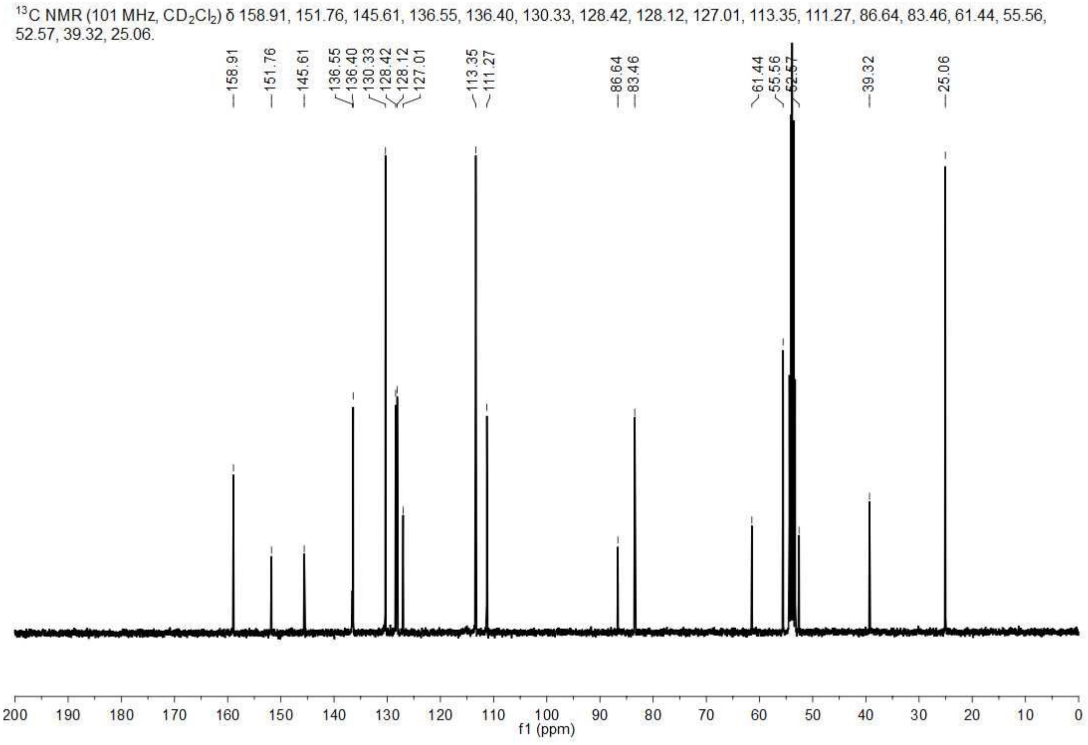

Figure S11. ^{13}C NMR spectrum of compound **5**.

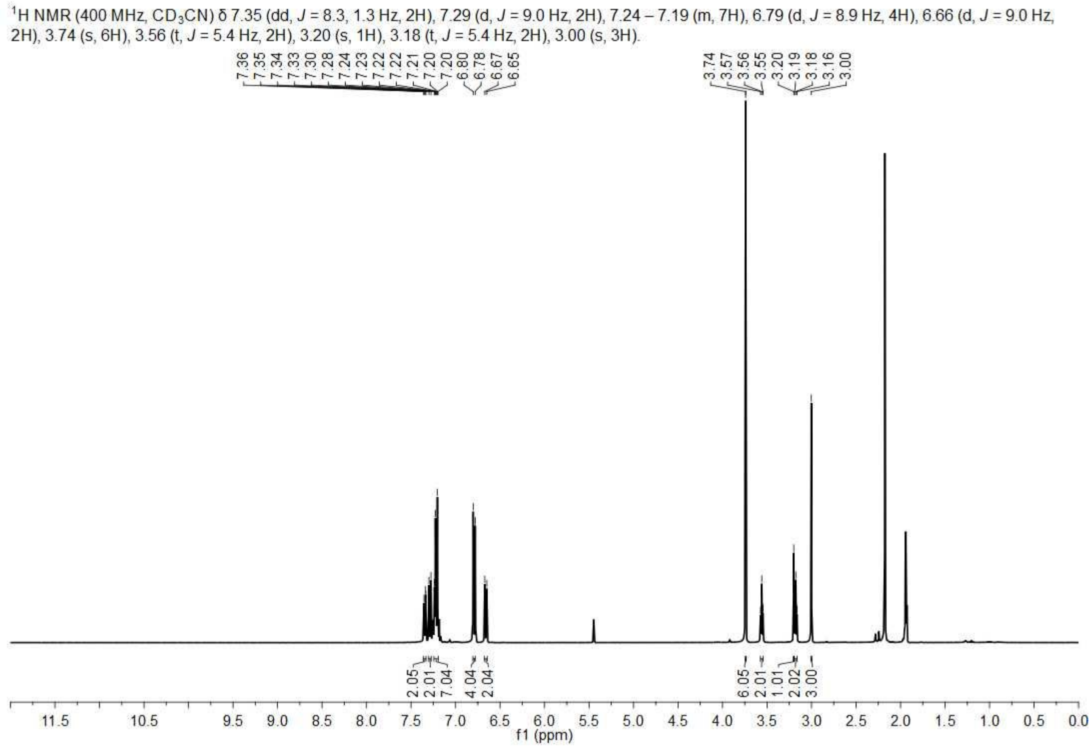

Figure S12. ^{1}H NMR spectrum of compound **6**.

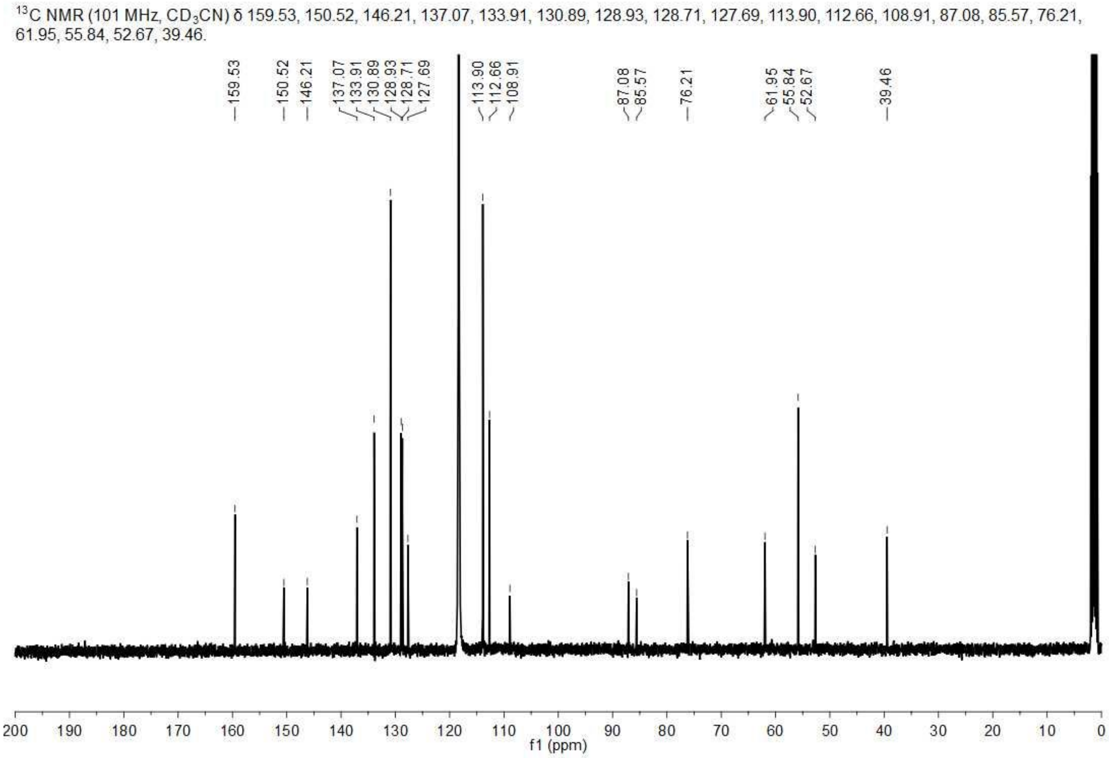

Figure S13. ^{13}C NMR spectrum of compound **6**.

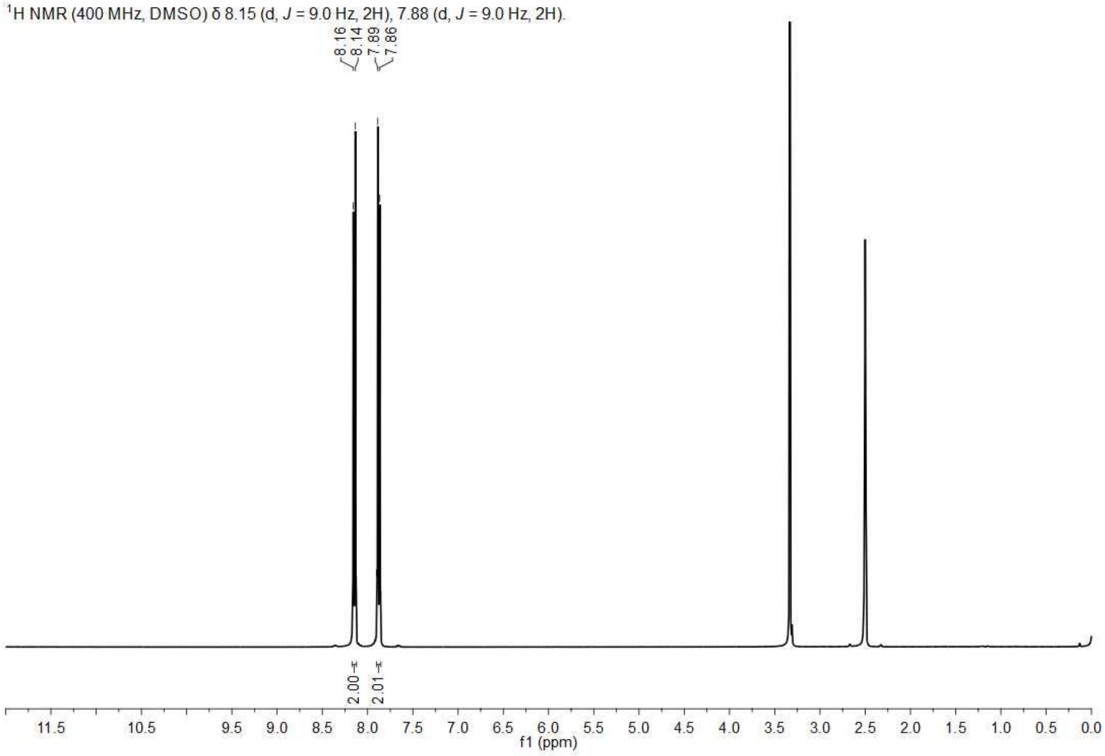

Figure S14. ^{1}H NMR spectrum of compound **7**.

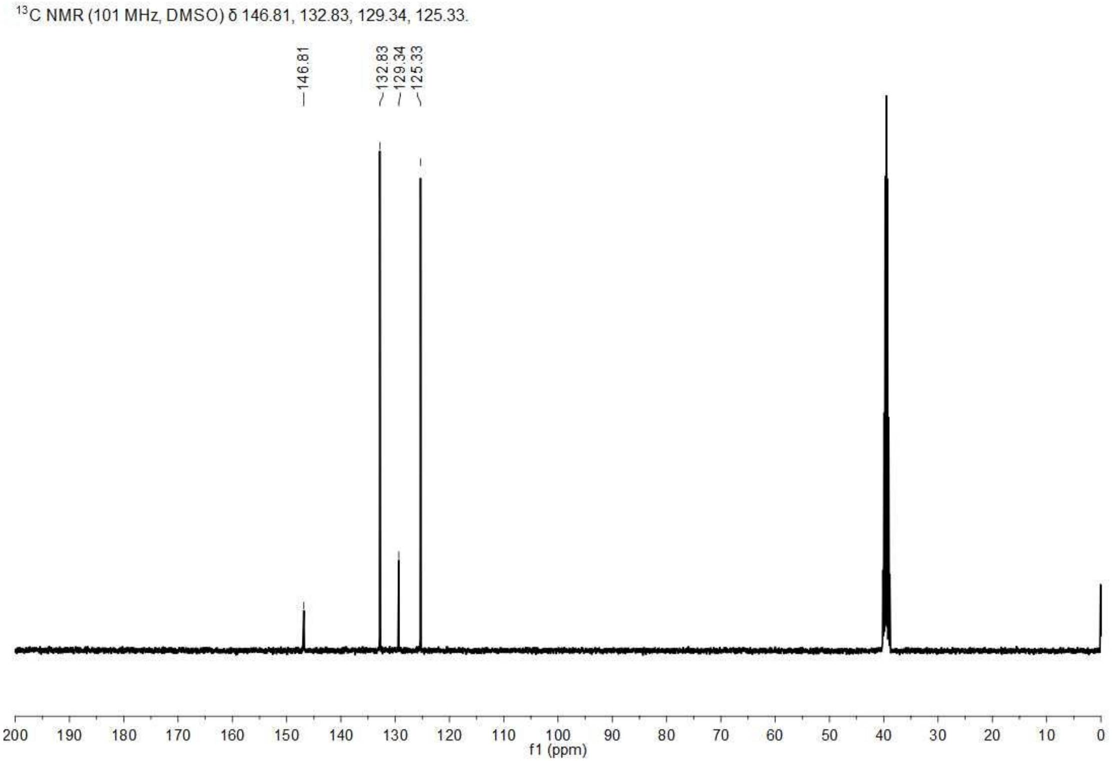

Figure S15. ^{13}C NMR spectrum of compound **7**.

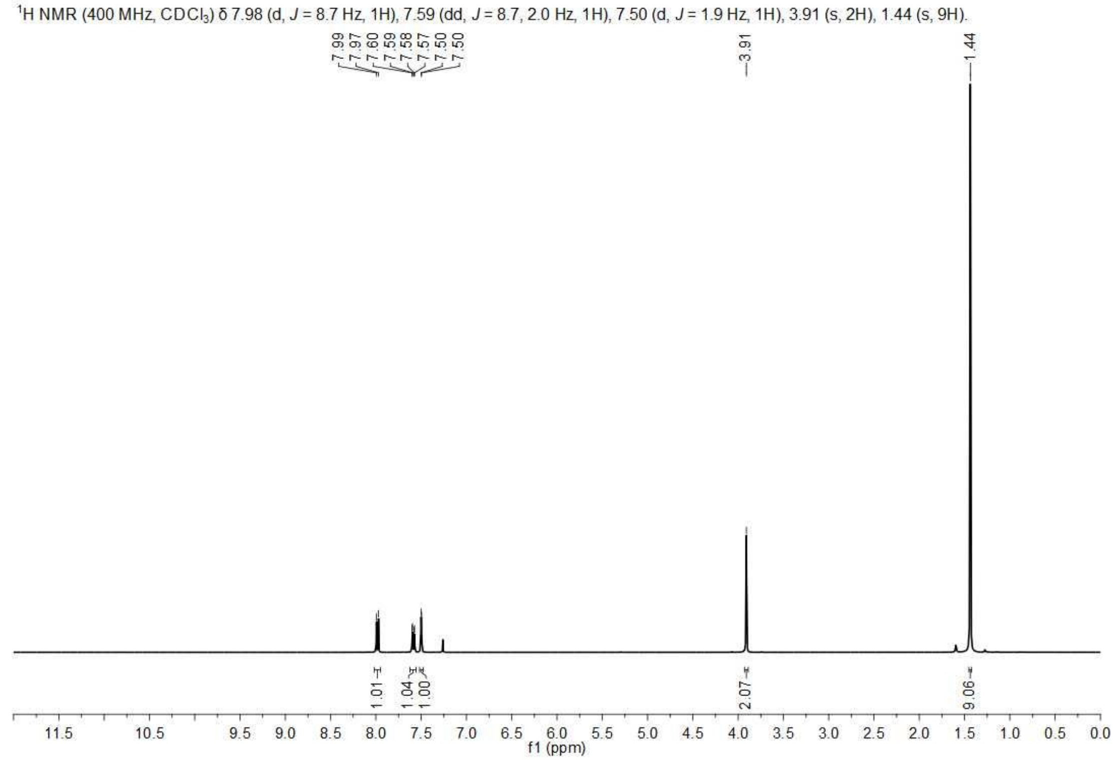

Figure S16. ^{1}H NMR spectrum of compound **8.**

^{13}C NMR (101 MHz, $CDCl_3$) δ 168.63, 147.81, 136.29, 132.49, 131.65, 128.34, 126.80, 82.35, 40.94, 28.07.

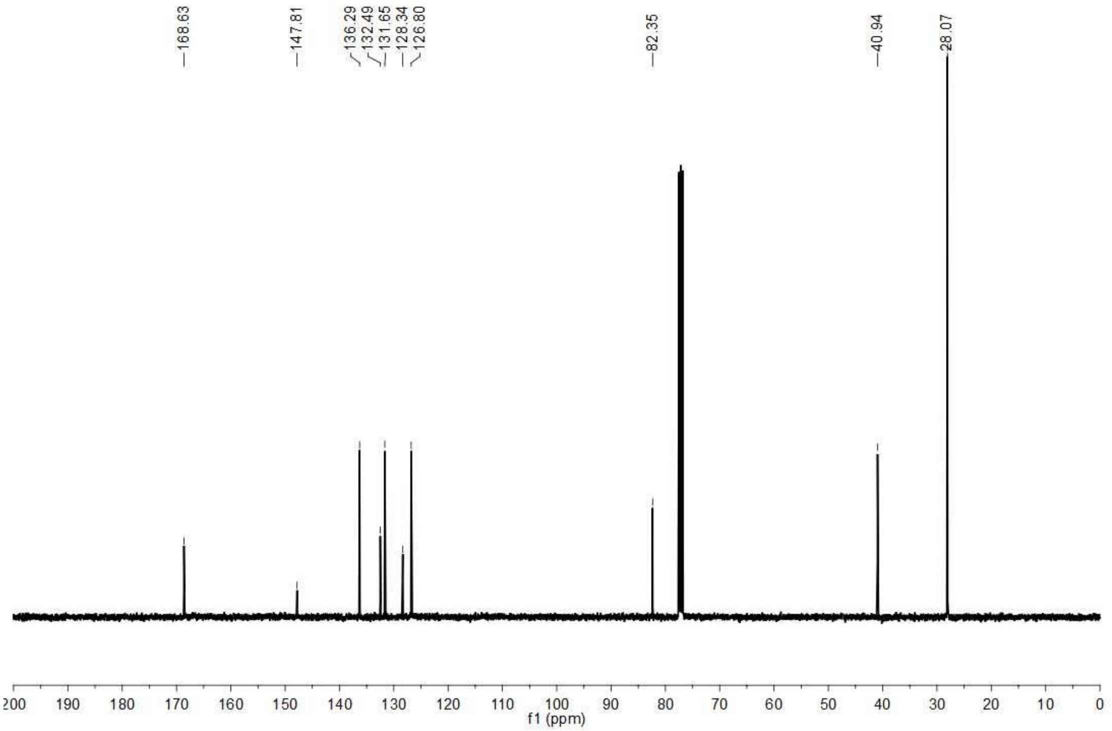

Figure S17. ^{13}C NMR spectrum of compound **8**.

^{1}H NMR (300 MHz, $CDCl_3$) δ 7.82 (d, J = 8.7 Hz, 1H), 7.62 (d, J = 2.1 Hz, 1H), 7.54 (dd, J = 8.7, 2.1 Hz, 1H), 4.21 (q, J = 7.2 Hz, 1H), 1.57 (d, J = 7.2 Hz, 3H), 1.40 (s, 9H).

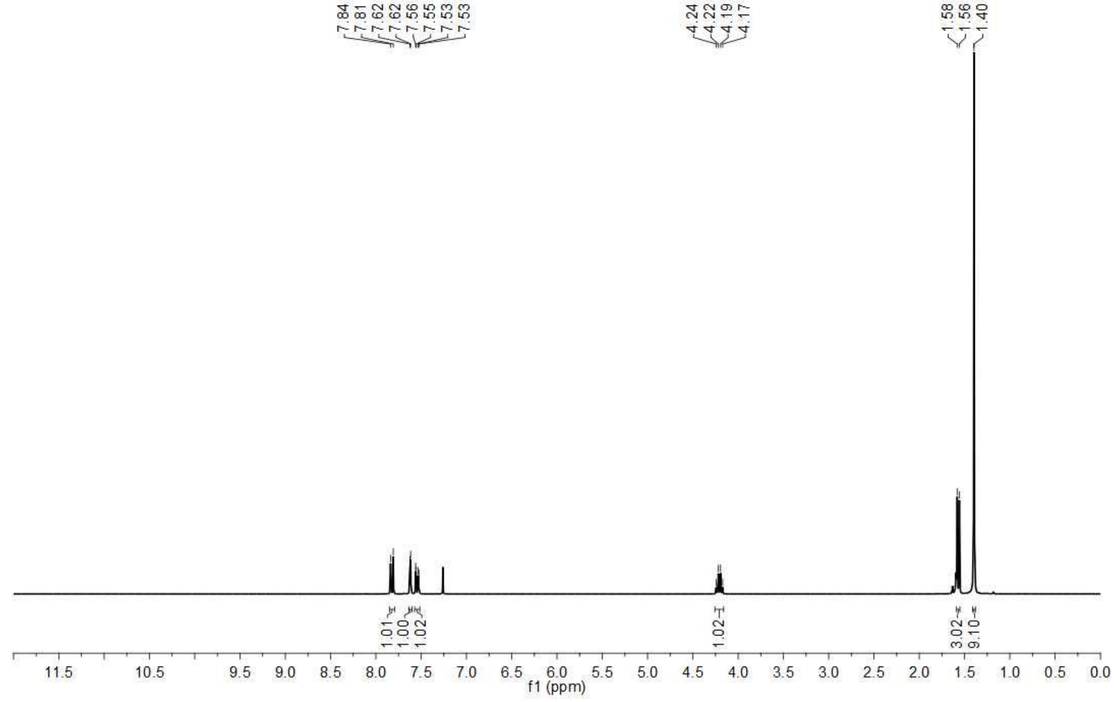

Figure S18. ^{1}H NMR spectrum of compound **9**.

^{13}C NMR (75 MHz, $CDCl_3$) δ 171.83, 147.99, 137.79, 132.95, 131.12, 128.14, 126.46, 81.99, 42.38, 27.95, 17.49.

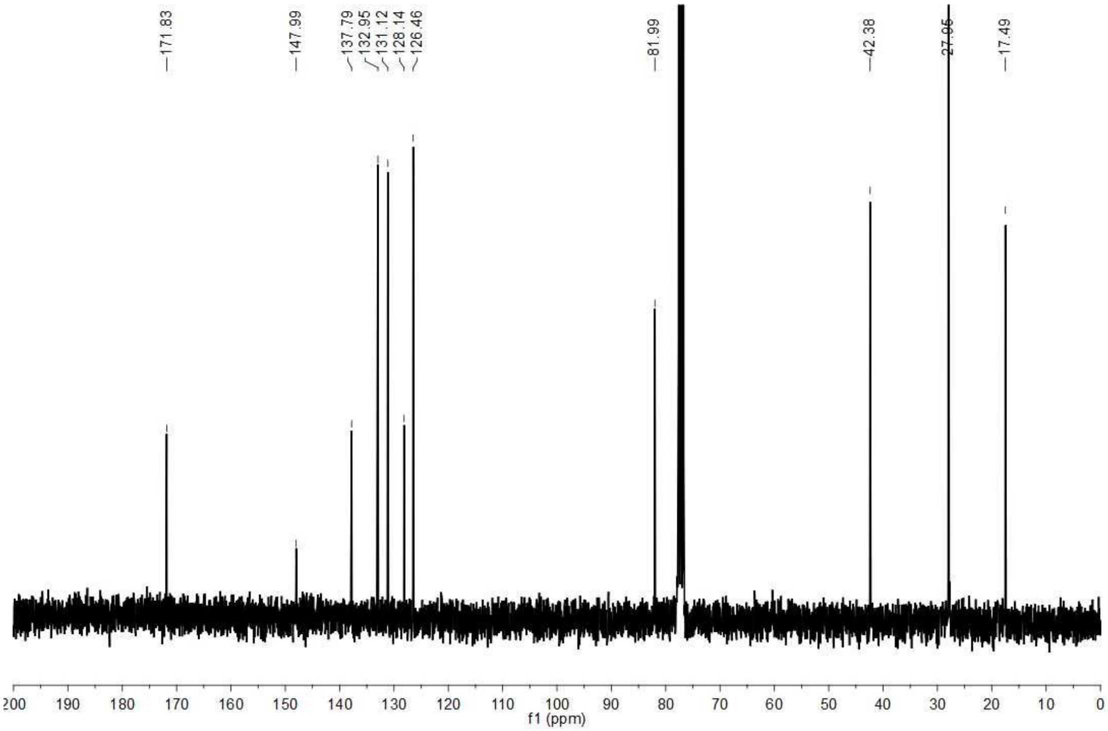

Figure S19. ^{13}C NMR spectrum of compound **9**.

^{1}H NMR (400 MHz, $CDCl_3$) δ 7.65 (d, J = 8.6 Hz, 1H), 7.63 (d, J = 2.0 Hz, 1H), 7.49 (dd, J = 8.6, 2.0 Hz, 1H), 3.81 (dd, J = 10.8, 5.8 Hz, 1H), 3.74 (dd, J = 10.8, 7.2 Hz, 1H), 3.61 – 3.50 (m, 1H), 1.32 (d, J = 6.9 Hz, 3H).

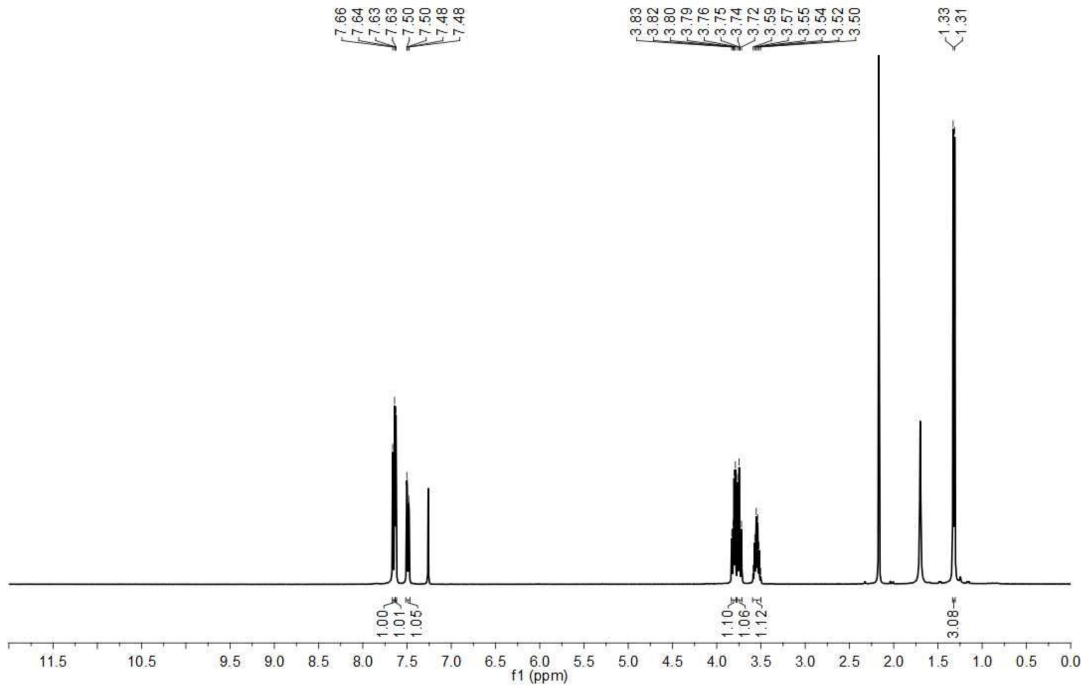

Figure S20. ^{1}H NMR spectrum of compound **10**.

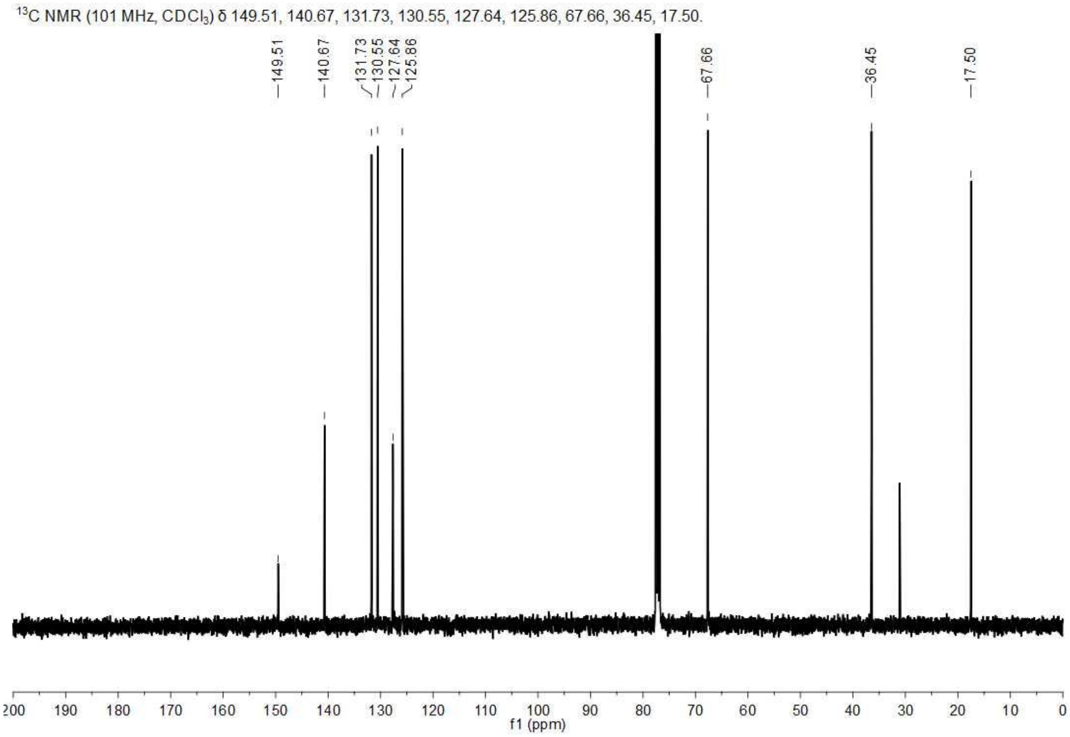

Figure S21. ^{13}C NMR spectrum of compound **10**.

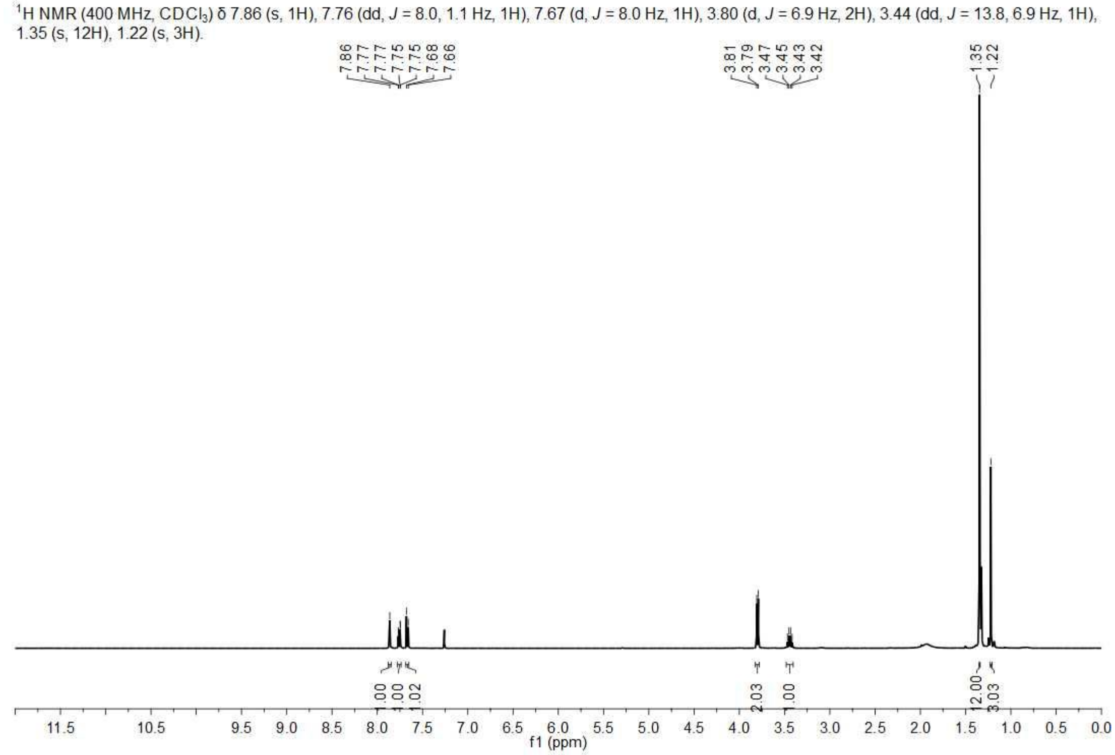

Figure S22. ^{1}H NMR spectrum of compound **11**.

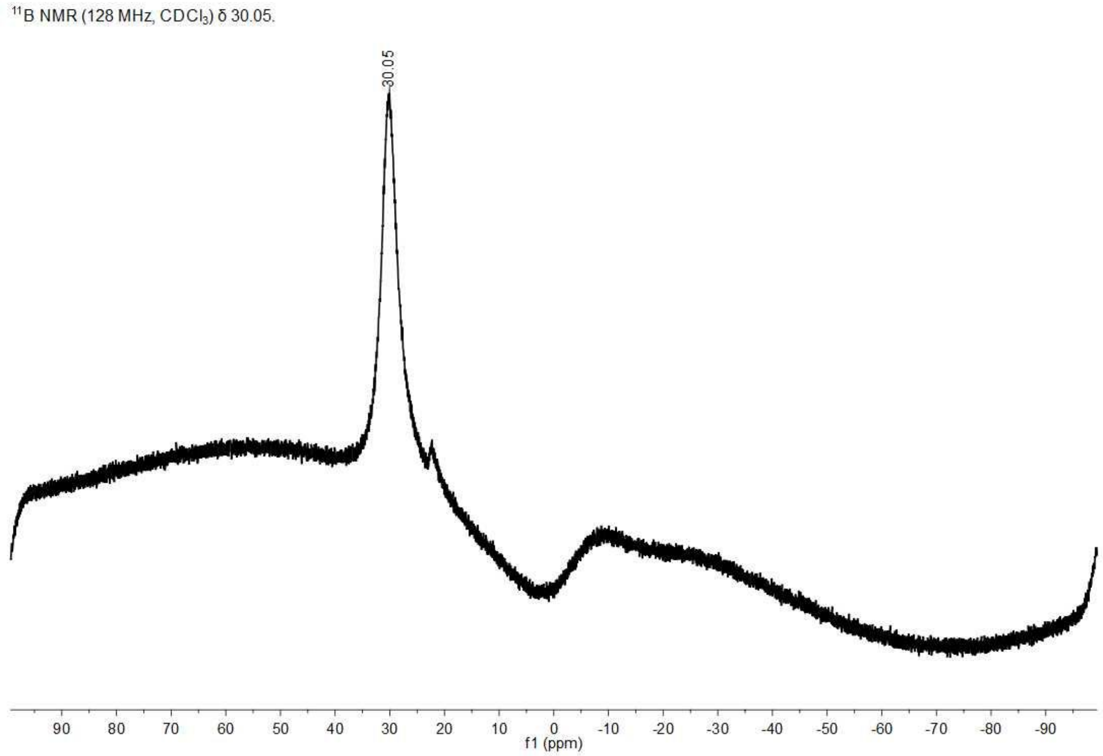

Figure S23. ^{11}B NMR spectrum of compound **11**.

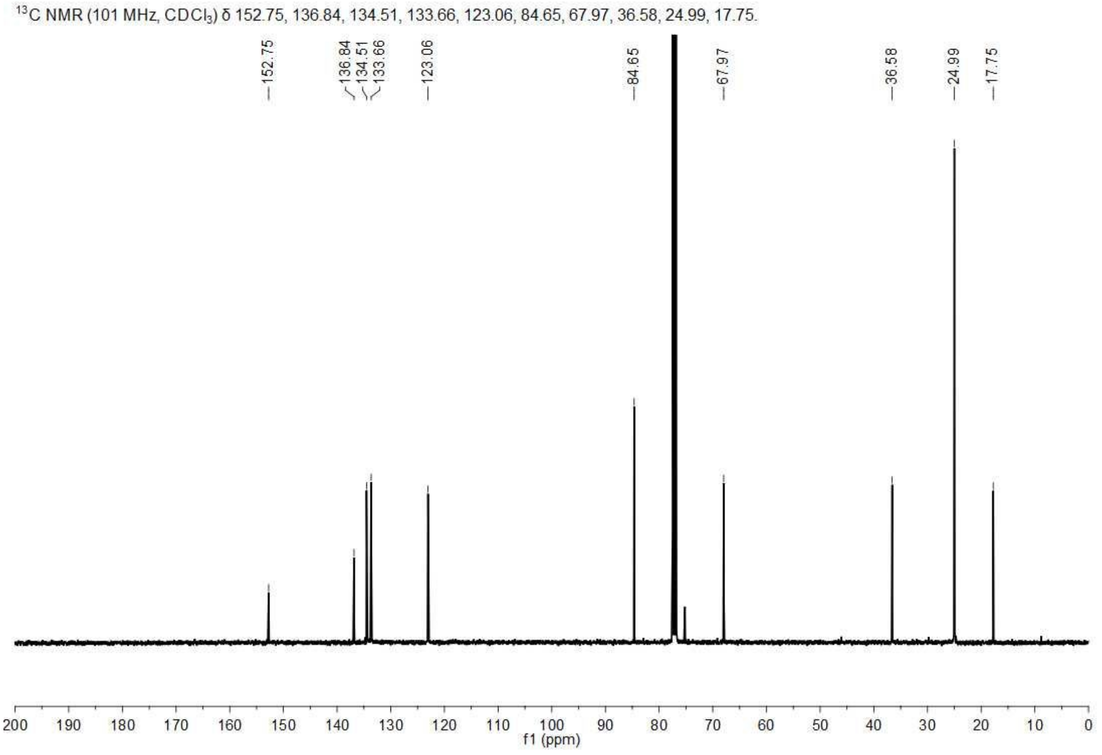

Figure S24. ^{13}C NMR spectrum of compound **11**.

^{1}H NMR (400 MHz, CD_2Cl_2) δ 7.84 (d, *J* = 8.5 Hz, 1H), 7.65 (dd, *J* = 5.0, 1.7 Hz, 1H), 7.53 (dd, *J* = 7.9, 2.6 Hz, 3H), 7.42 – 7.38 (m, 2H), 7.29 – 7.17 (m, 7H), 6.81 – 6.76 (m, 6H), 3.81 (d, *J* = 6.1 Hz, 2H), 3.74 (s, 6H), 3.68 – 3.64 (m, 1H), 3.60 (t, *J* = 5.7 Hz, 2H), 3.29 (t, *J* = 5.6 Hz, 2H), 3.08 (s, 3H), 1.37 (d, *J* = 6.9 Hz, 3H).

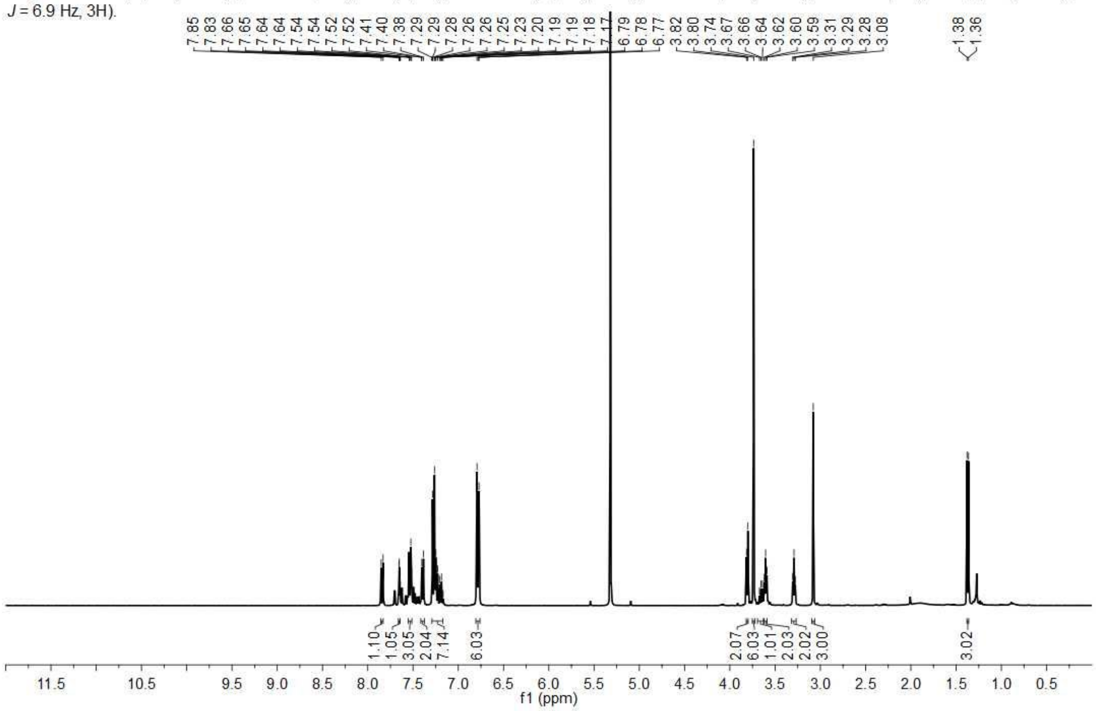

Figure S25. ^{1}H NMR spectrum of compound **12**.

^{13}C NMR (101 MHz, CD_2Cl_2) δ 158.89, 150.12, 148.42, 146.18, 145.53, 139.64, 136.48, 130.33, 129.41, 128.98, 128.40, 128.34, 128.11, 127.72, 127.31, 127.03, 126.08, 125.96, 125.48, 125.15, 124.42, 113.33, 112.58, 86.68, 68.18, 61.40, 55.53, 52.74, 39.40, 36.88, 17.78.

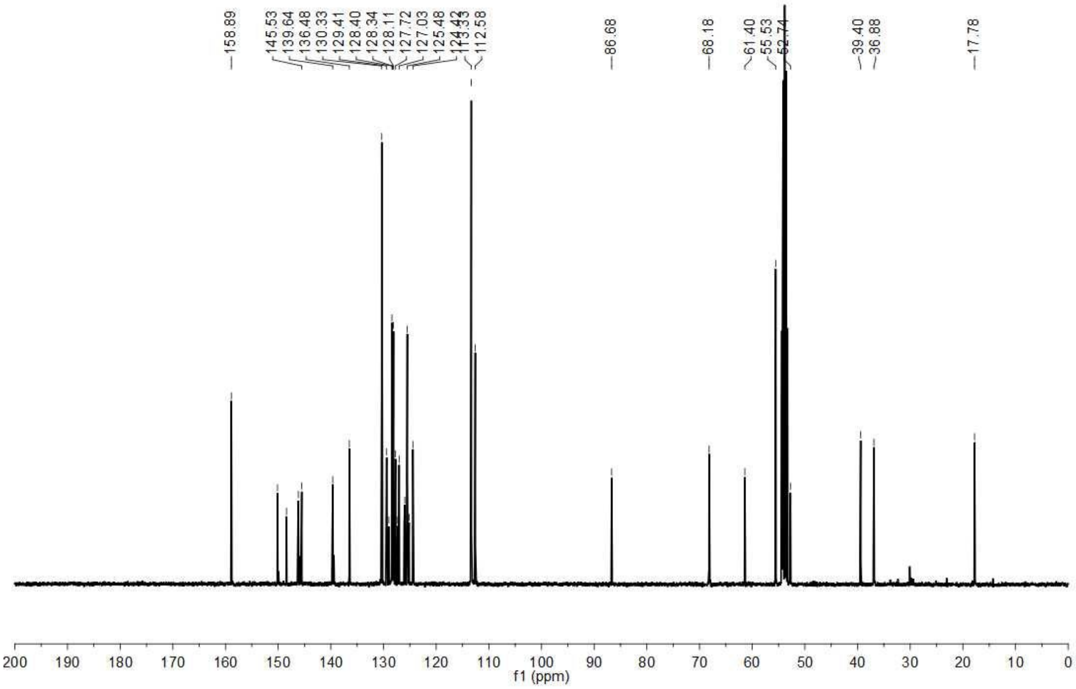

Figure S26. ^{13}C NMR spectrum of compound **12**.

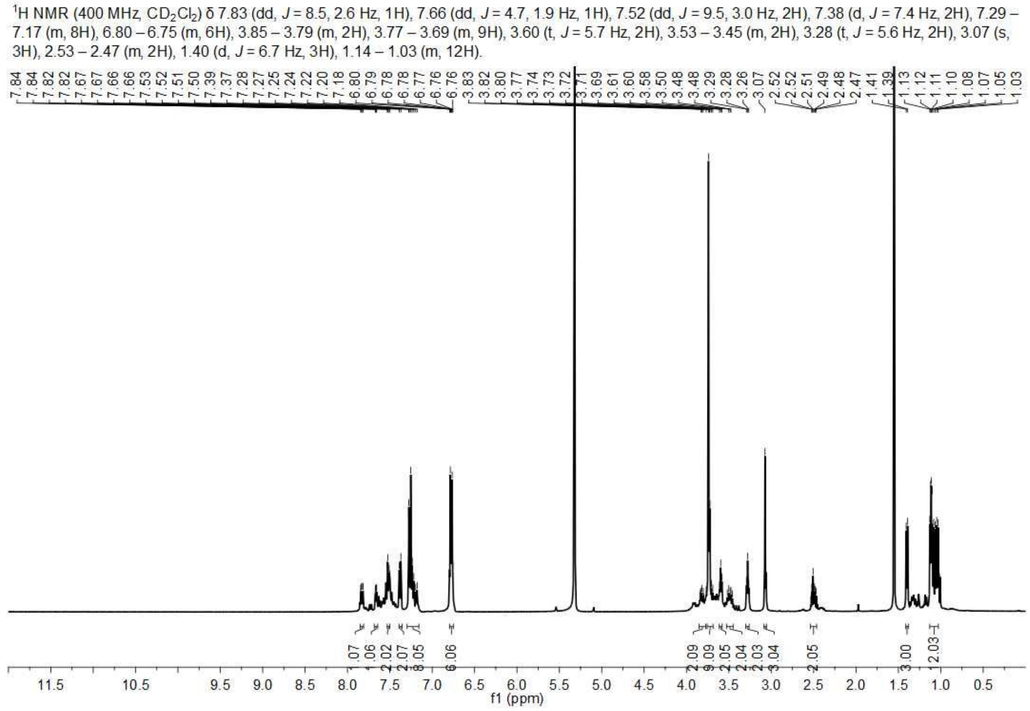

Figure S27. ^{1}H NMR spectrum of compound **13**.

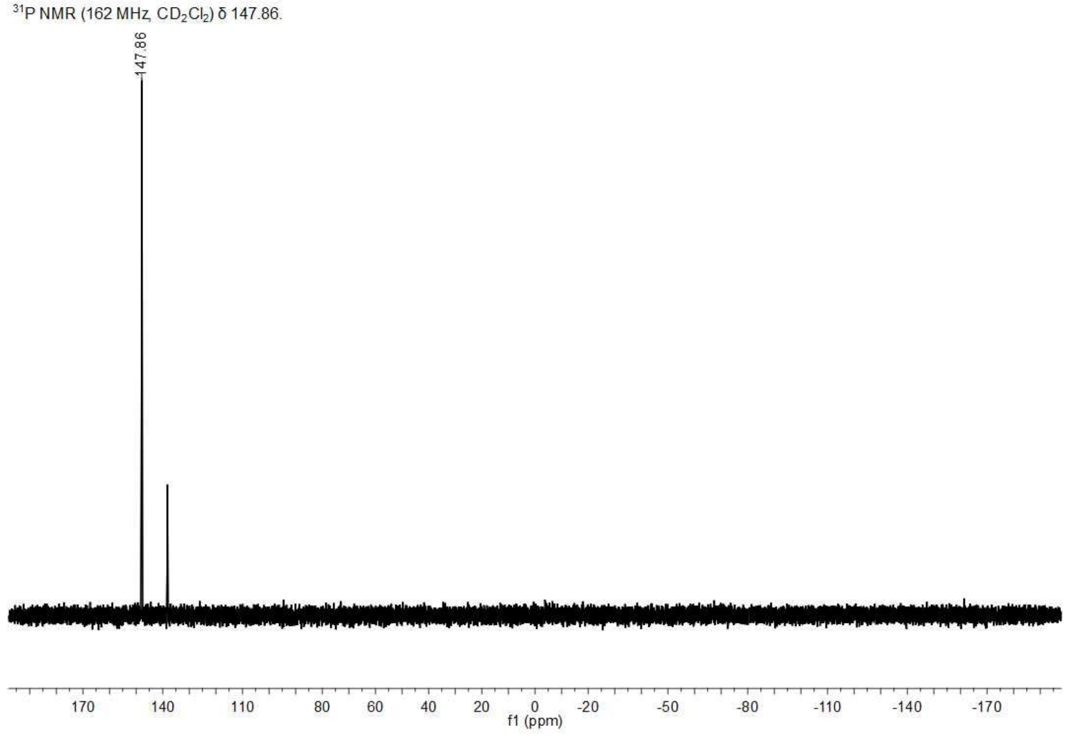

Figure S28. ^{31}P NMR spectrum of compound **13**.

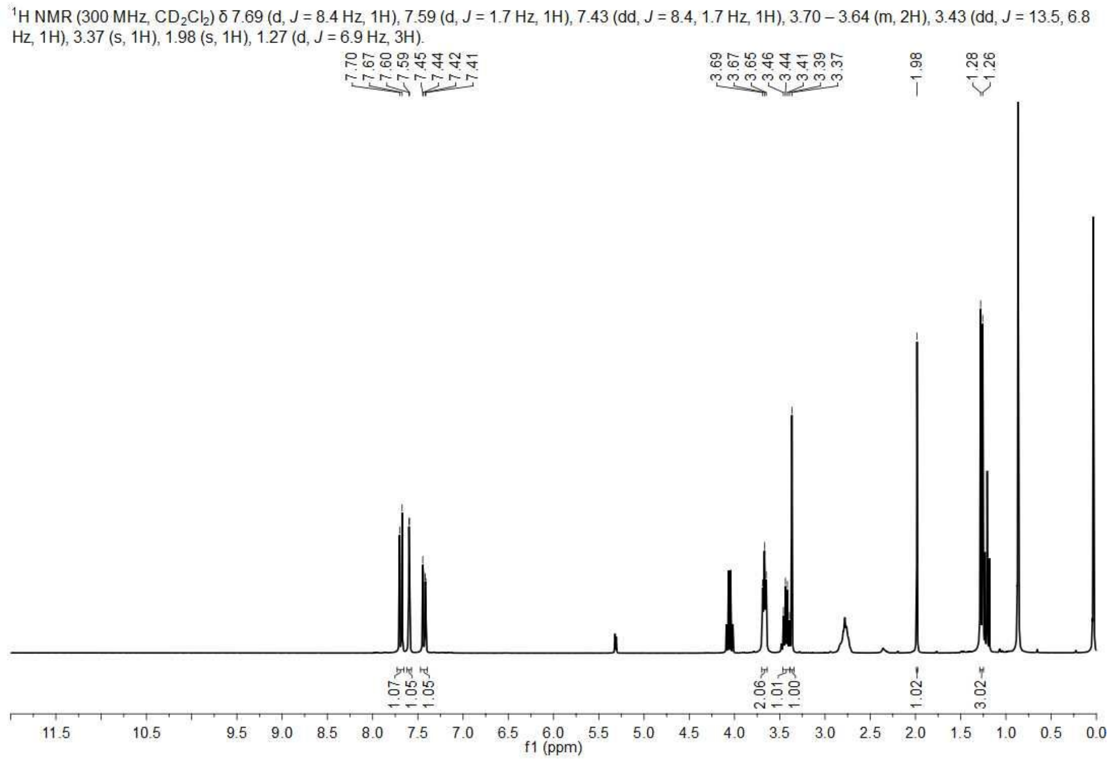

Figure S29. ^{1}H NMR spectrum of compound **14**.

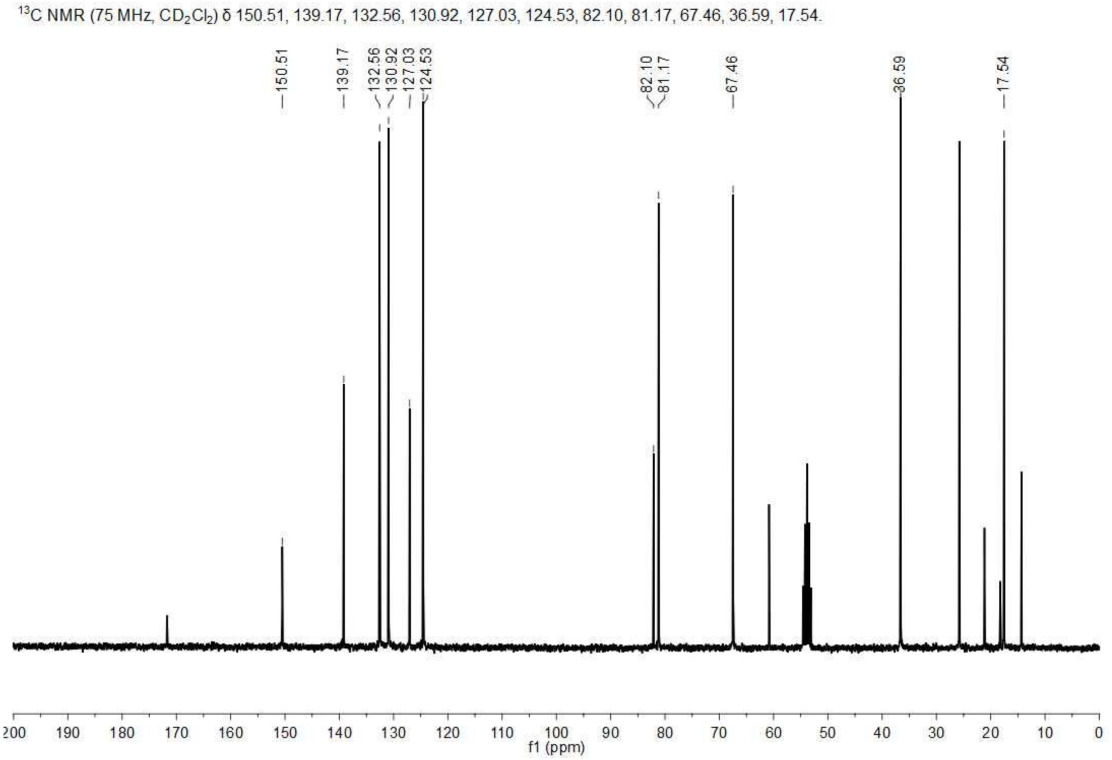

Figure S30. ^{13}C NMR spectrum of compound **14**.

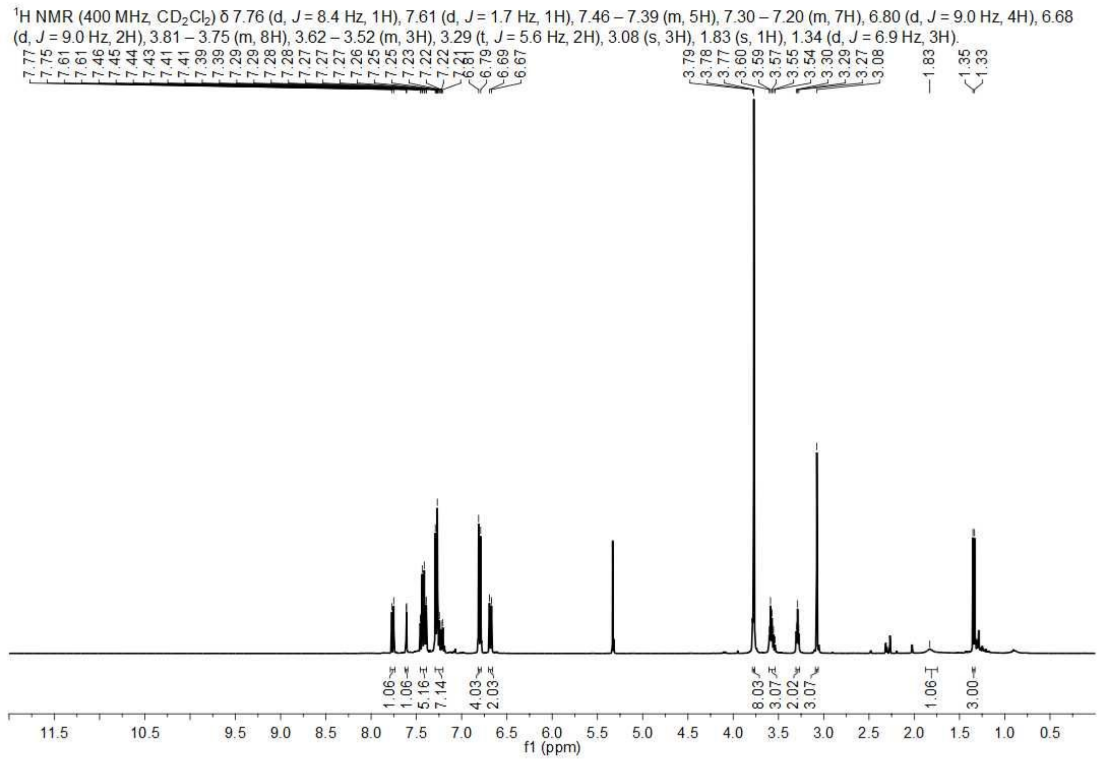

Figure S31. ^{1}H NMR spectrum of compound **15**.

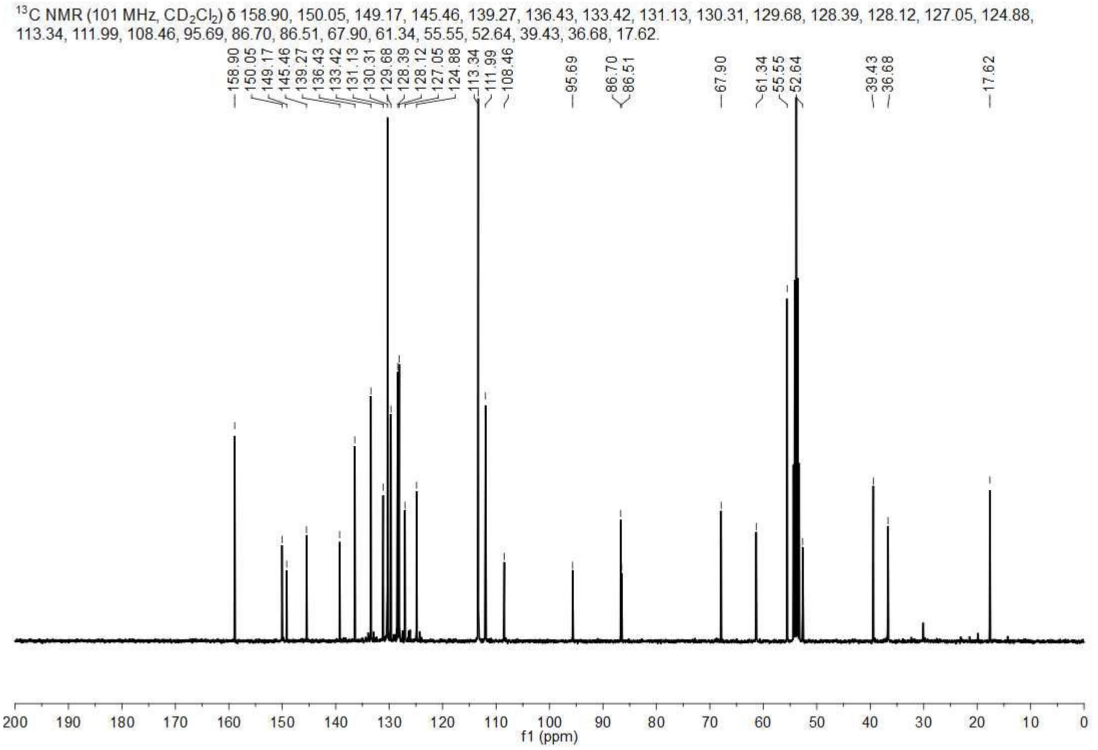

Figure S32. ^{13}C NMR spectrum of compound **15**.

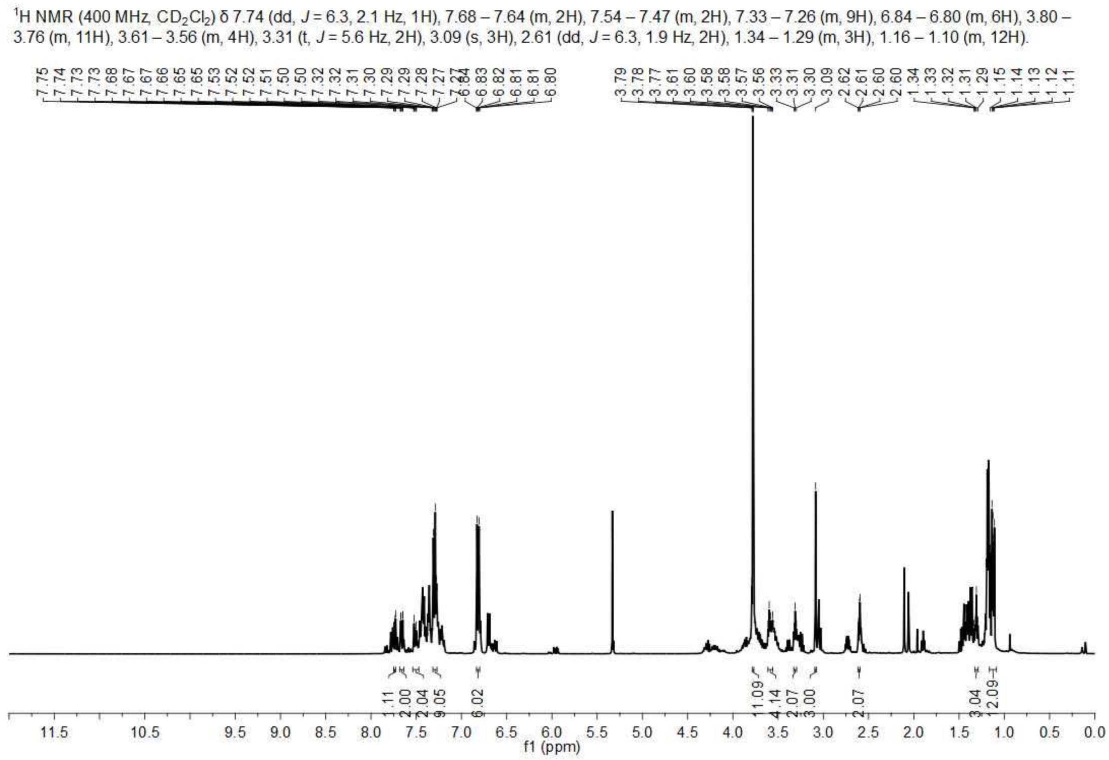

Figure S33. ^{1}H NMR spectrum of compound **16**.

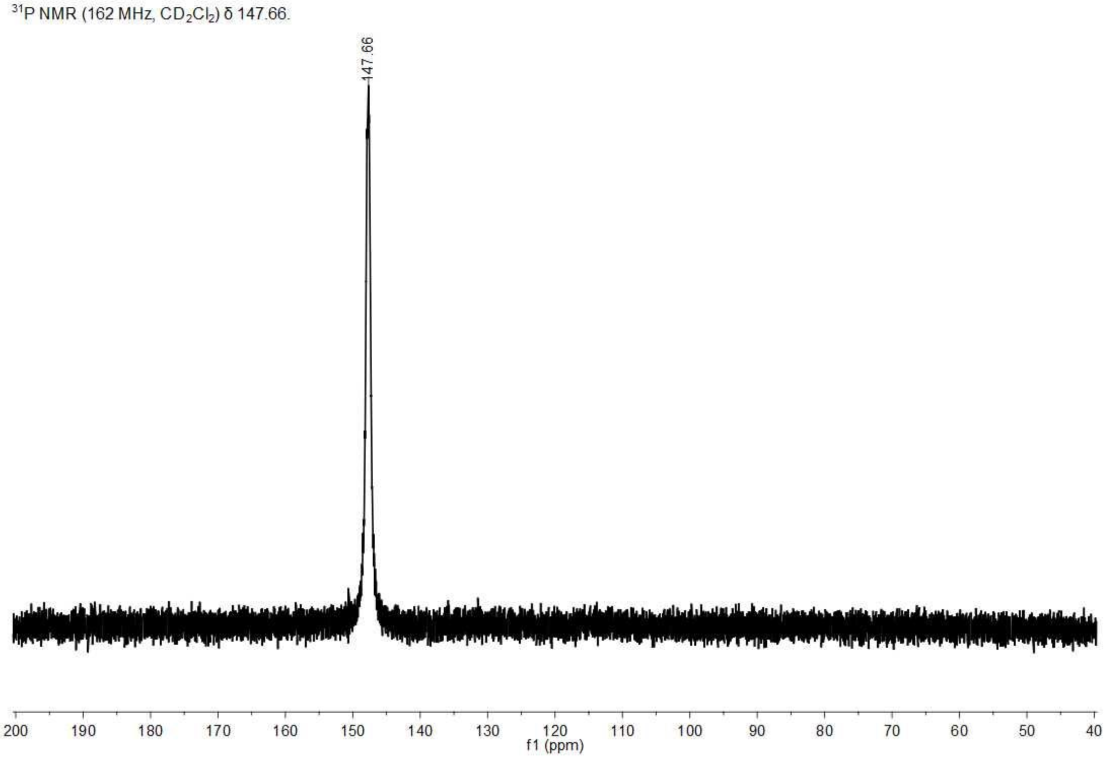

Figure S34. ^{31}P NMR spectrum of compound **16**.

Name	Sequence (5' → 3')
Cy3-d15	Cy3/CTGAGACTTTAATAA
d19-FAM	TTGAAATTCACCTGGTAGC/FAM
Cy3-d15-*s*-ANBP-d19-FAM	Cy3/CTGAGACTTTAATAA/*s*-ANBP/TTGAAATTCACCTGGTAGC/FAM
Cy3-d15-*t*-ANBP-d19-FAM	Cy3/CTGAGACTTTAATAA/*t*-ANBP/TTGAAATTCACCTGGTAGC/FAM

Table S1. Sequences of DNA oligonucleotides for photolytic study of single-strand samples.

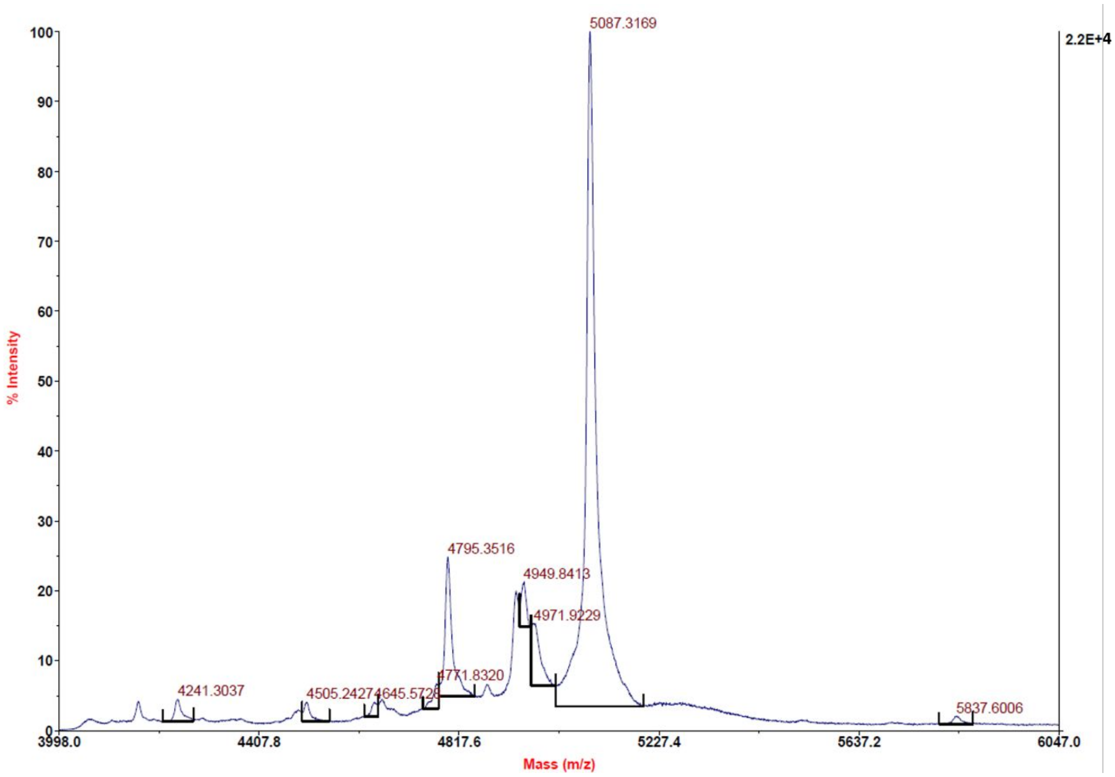

Figure S35. MALDI-TOF spectrum of Cy3-d15 with the peak detected at m/z 5087.3169 $[M]^+$ (calculated: 5082.68 g/mol).

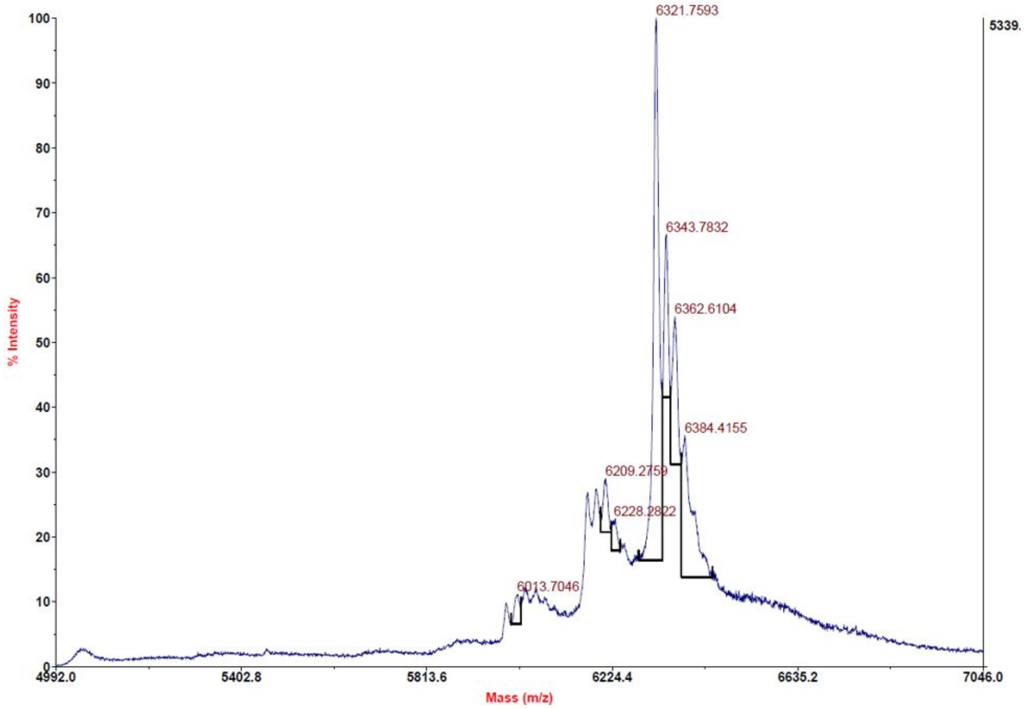

Figure S36. MALDI-TOF spectrum of d19-FAM with the peak detected at m/z 6321.7593 $[M]^+$ (calculated: 6371.26 g/mol).

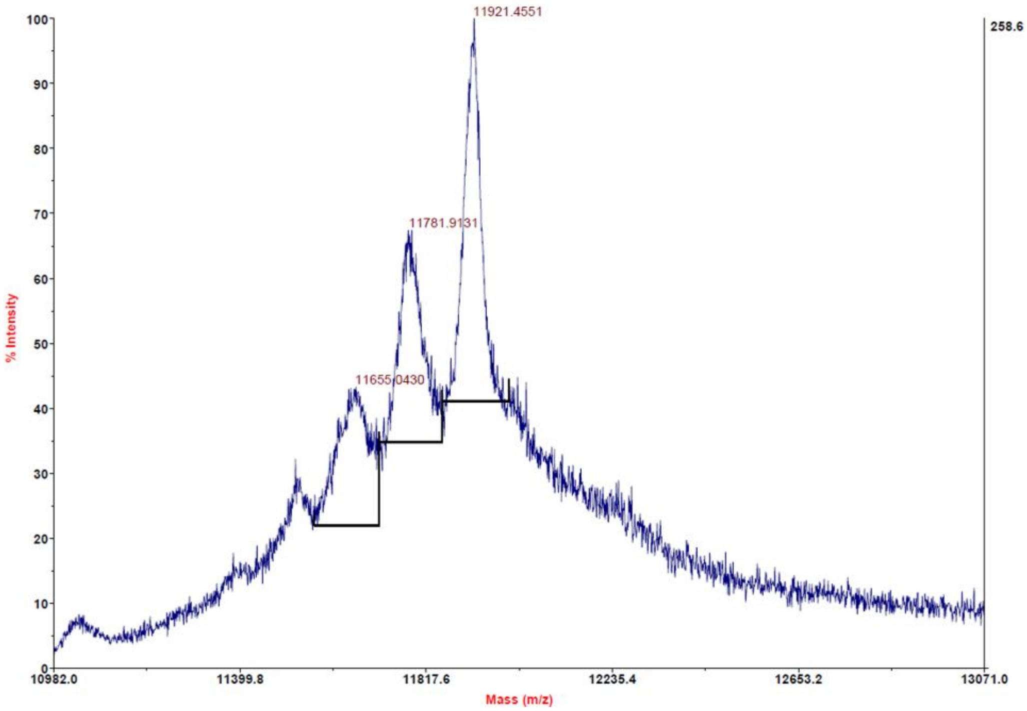

Figure S37. MALDI-TOF spectrum of Cy3-d15-s-ANBP-d19-FAM with the peak detected at m/z 11921.4551 $[M]^+$ (calculated: 11844.21 g/mol).

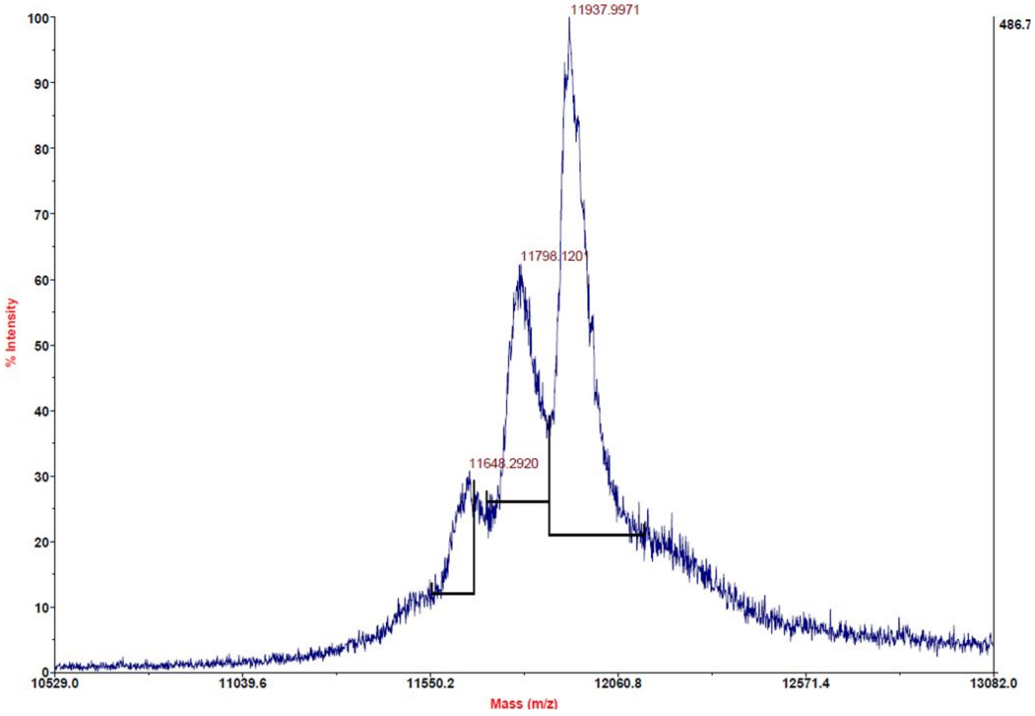

Figure S38. MALDI-TOF spectrum of Cy3-d15-t-ANBP-d19-FAM with the peak detected at m/z 11937.9971 $[M]^+$ (calculated: 11868.23 g/mol).

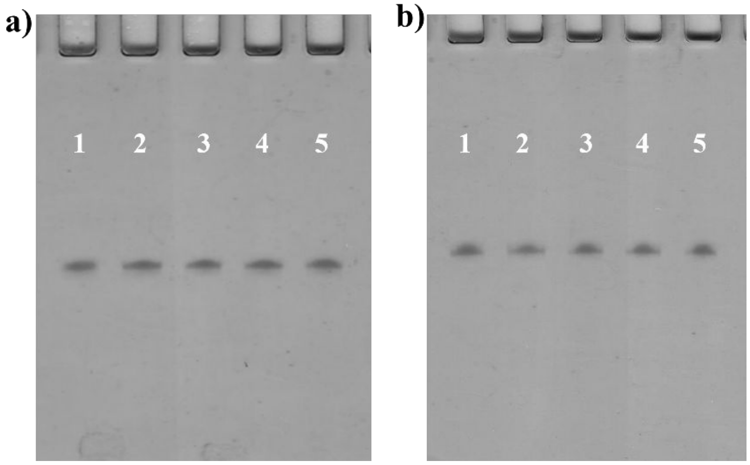

Figure S39. Stability analysis of a) Cy3-d15-s-ANBP-d19-FAM and b) Cy3-d15-t-ANBP-d19-FAM under water (lane 1), 500 mM potassium chloride solution (lane 2), 50 mM sodium acetate solution at pH 4 (lane 3), 50 mM sodium carbonate-sodium bicarbonate buffer at pH 10 (lane 4) and 37 ^oC for 24 h (lane 5) by 15 % denaturing PAGE.

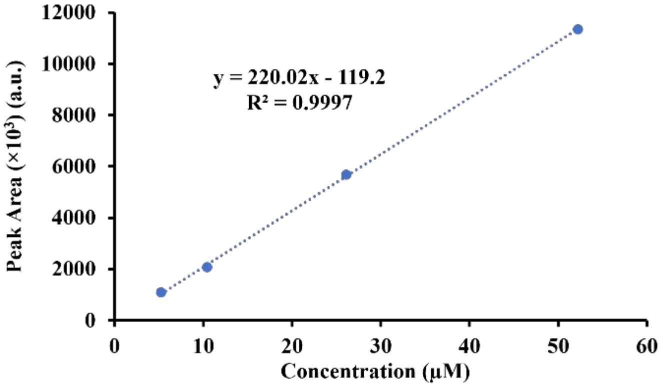

Figure S40. Calibration curve by d19-FAM for HPLC quantification of photocleaved d15-s-ANBP-d19-FAM and d15-t-ANBP-d19-FAM.

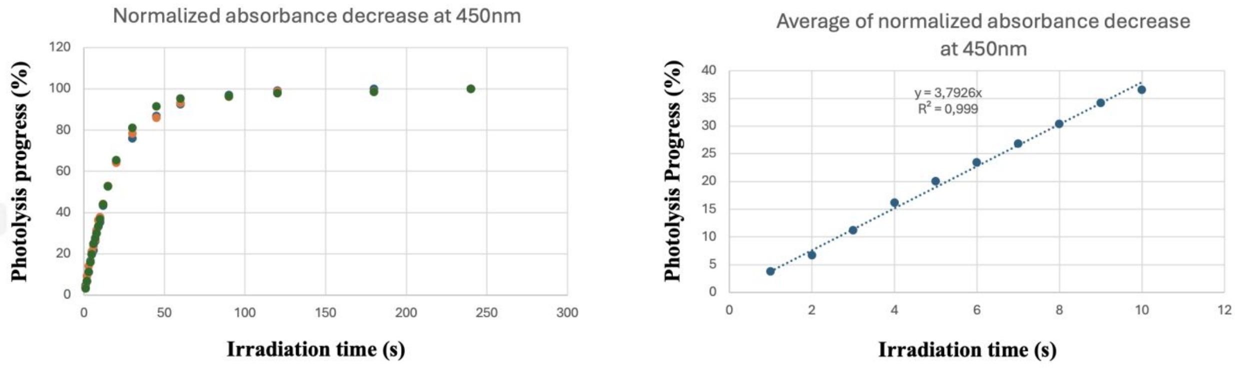

Figure S41: One-photon uncaging kinetics for DEACAS-*p*-methoxybenzoic (ε_{405nm}= 27500 $M^{-1}cm^{-1}$)

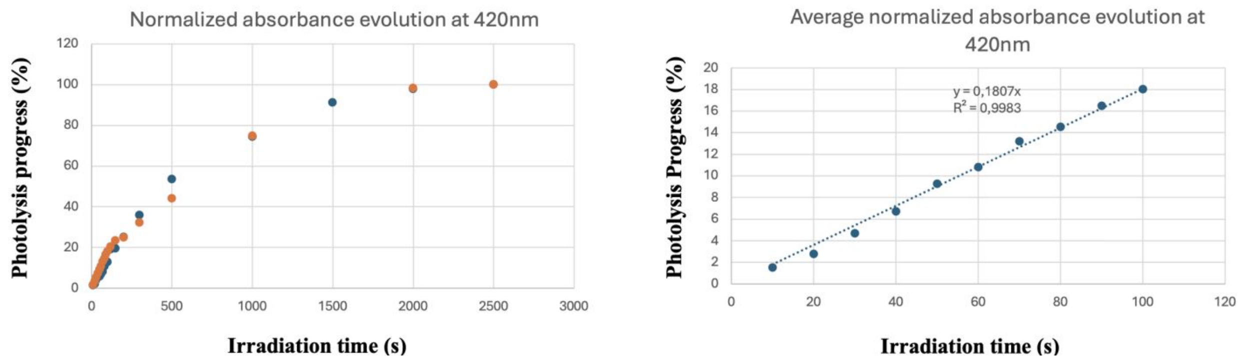

Figure S42: One-photon uncaging kinetics for s-ANBP-conjugated DNA (ε_{405nm}= 28450 $M^{-1}cm^{-1}$)

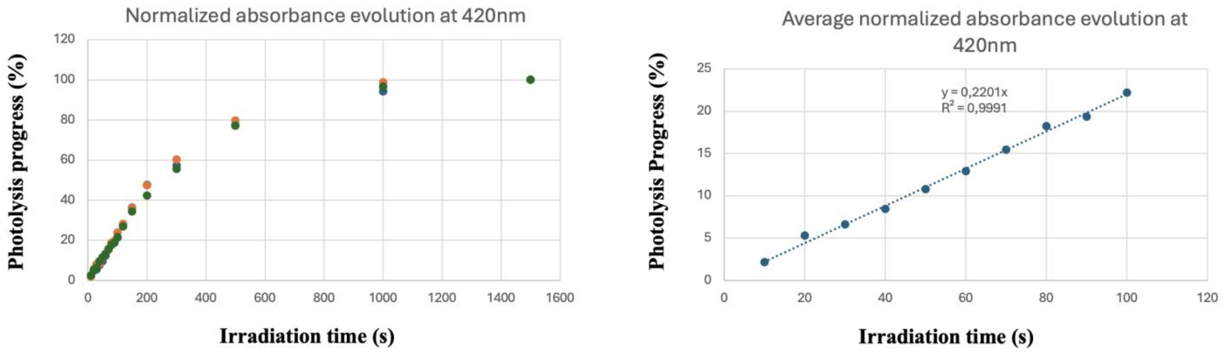

Figure S43: One-photon uncaging kinetics for t-ANBP-conjugated DNA (ε_{405nm}= 28750 $M^{-1}cm^{-1}$)

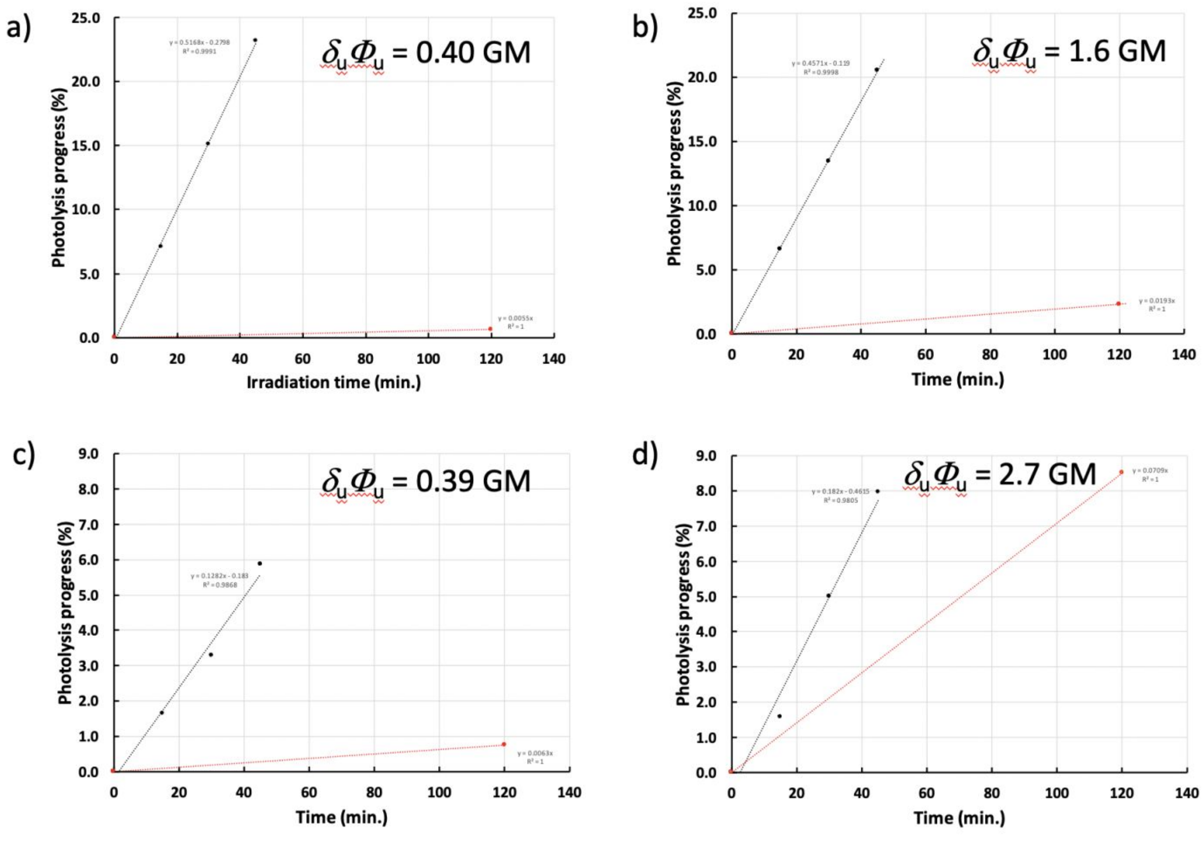

Figure S44. Two-photon photolysis progress of s-ANBP- (a and c) and t-ANBP-conjugated DNA (b and d) in red upon irradiation at 740 nm (a and b) and 800 nm (c and d) with fs pulsed laser. Black curves show the photolysis progress of the reference molecule DEACAS.[1]

Name	Sequence (5' → 3')
Insert 1 (s-I1)	AACGGAGGCT/s-ANBP/GGGATGCCTT
Insert 2 (s-I2)	AACGGA/s-ANBP/GGCTGGGA/s-ANBP/TGCCTT
Eliminate 1 (s-IE1)	AACGGAGGC/s-ANBP/GGATGCCTT
Eliminate 2 (s-IE2)	AACGG/s-ANBP/GCTGGG/s-ANBP/GCCTT
4625-FAM	AAGGCATCCCAGCCTCCGTT/FAM
s-IE2-BHQ	BHQ/AACGG/s-ANBP/GCTGGG/s-ANBP/GCCTT
t-IE2-BHQ	BHQ/AACGG/t-ANBP/GCTGGG/t-ANBP/GCCTT

Table S2. Sequences of DNA oligonucleotides for photolytic study of duplex samples. FAM represents 6-fluorescein. s-ANBP represents ANBP molecule with a single-bond. BHQ represents black hole quencher 1.

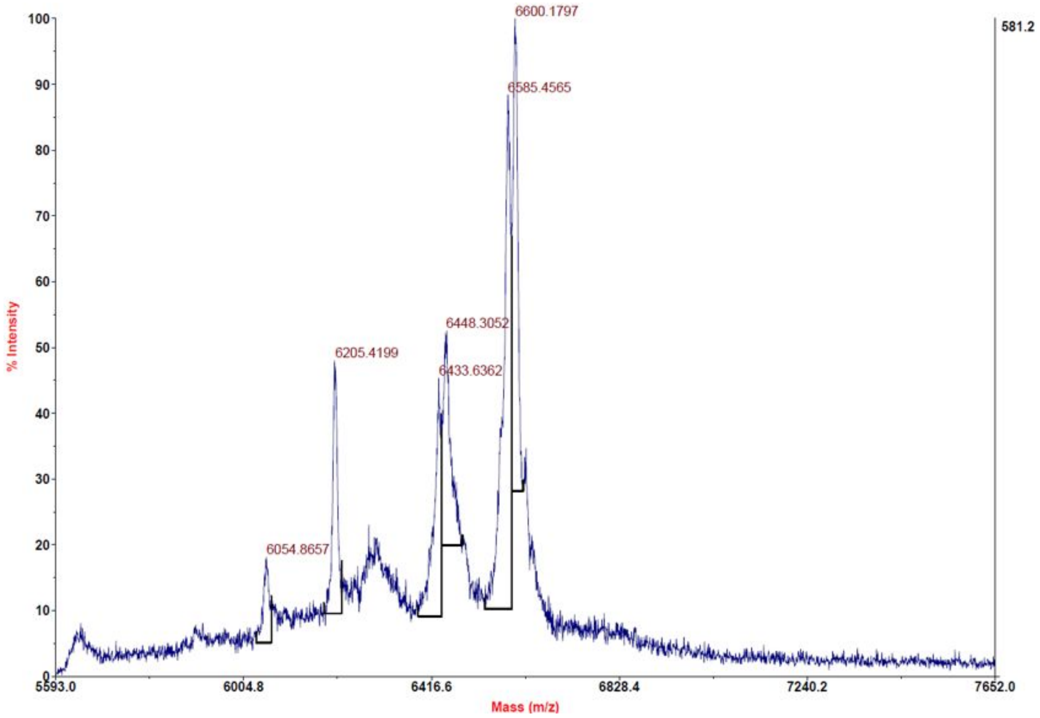

Figure S45. MALDI-TOF spectrum of s-I1 with the peak detected at m/z 6600.1797 $[M]^+$ (calculated: 6589.44 g/mol).

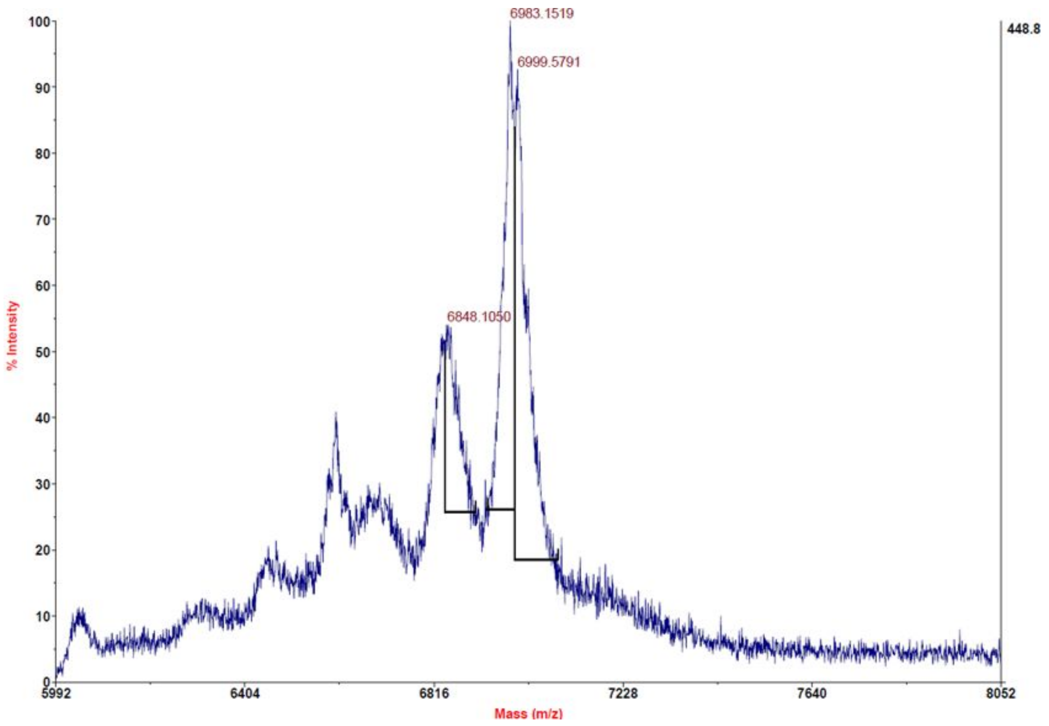

Figure S46. MALDI-TOF spectrum of s-I2 with the peak detected at m/z 6983.1519 $[M]^+$ (calculated: 6980.78 g/mol).

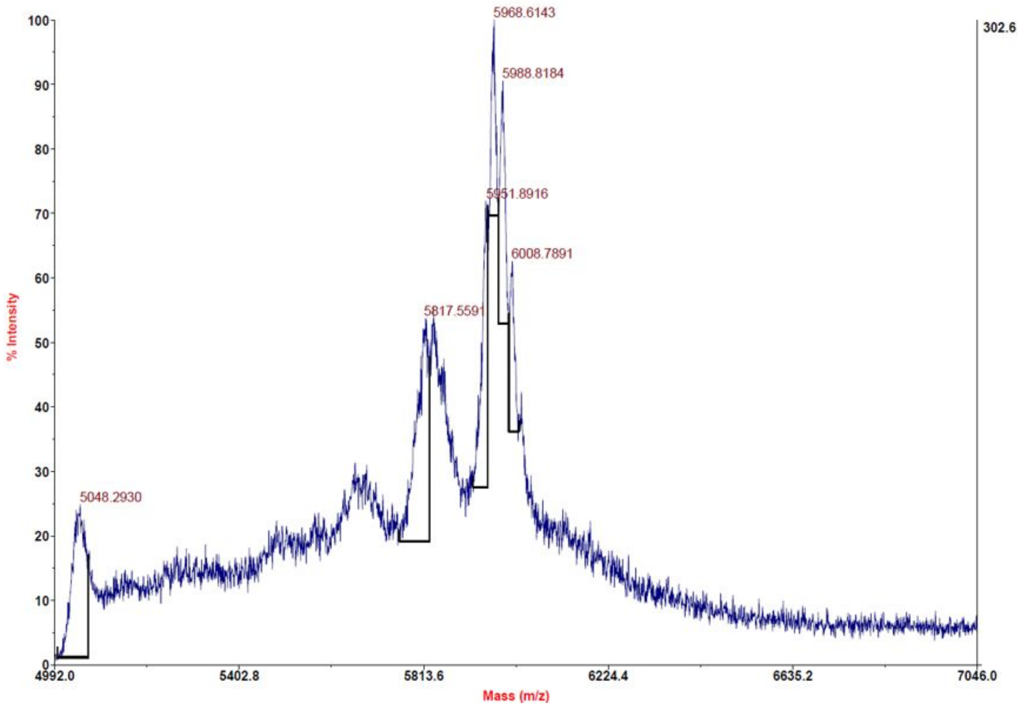

Figure S47. MALDI-TOF spectrum of s-IE1 with the peak detected at m/z 5968.6143 $[M]^+$ (calculated: 5956.04 g/mol).

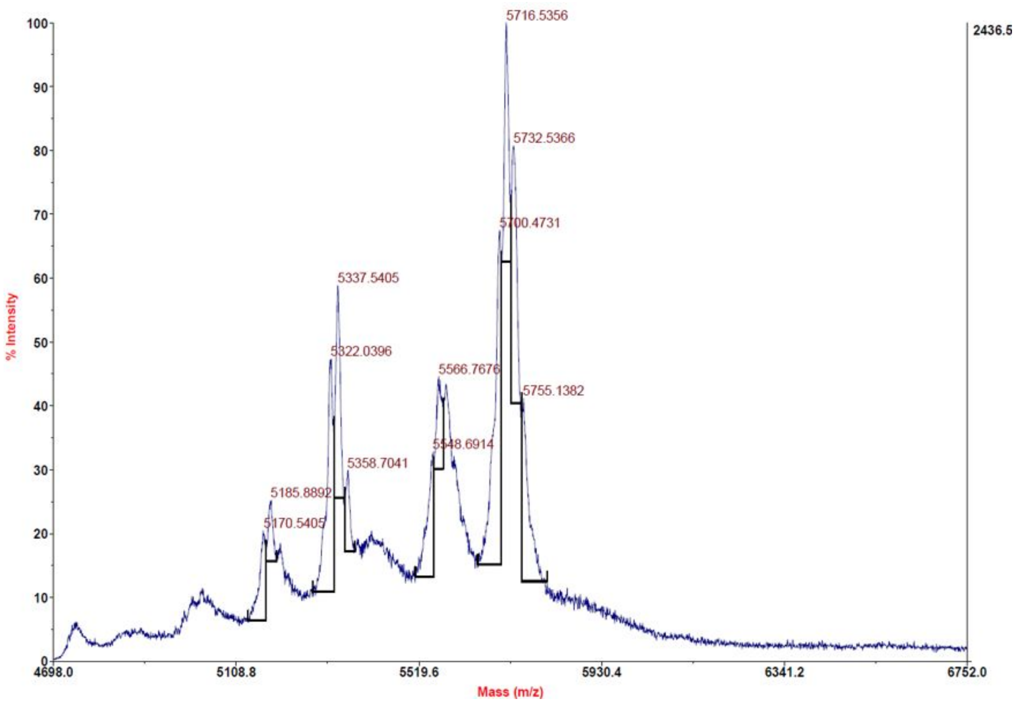

Figure S48. MALDI-TOF spectrum of s-IE2 with the peak detected at m/z 5716.5356 $[M]^+$ (calculated: 5720.88 g/mol).

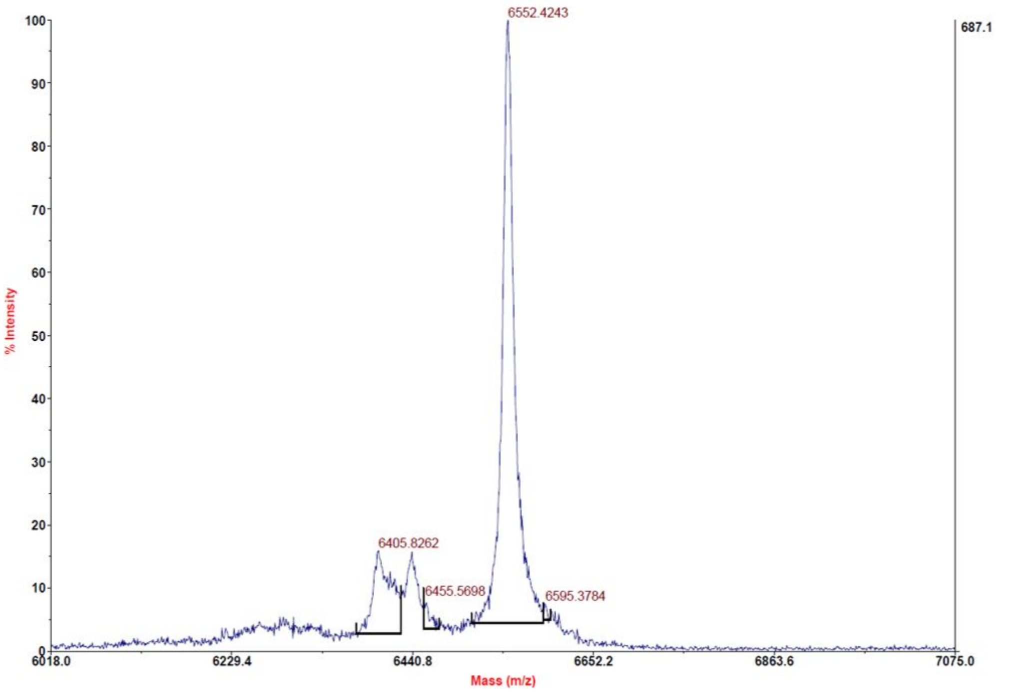

Figure S49. MALDI-TOF spectrum of ASO 4625-FAM with the peak detected at m/z 6552.4243 $[M]^+$ (calculated: 6607.46 g/mol).

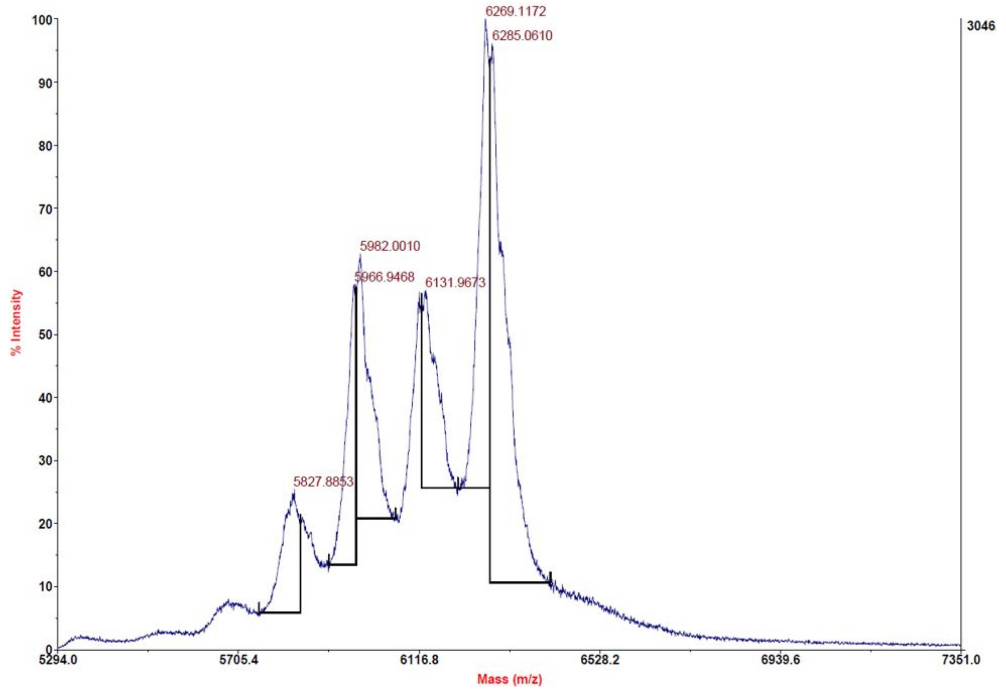

Figure S50. MALDI-TOF spectrum of s-IE2-BHQ with the peak detected at m/z 6269.1172 $[M]^+$ (calculated: 6275.37 g/mol).

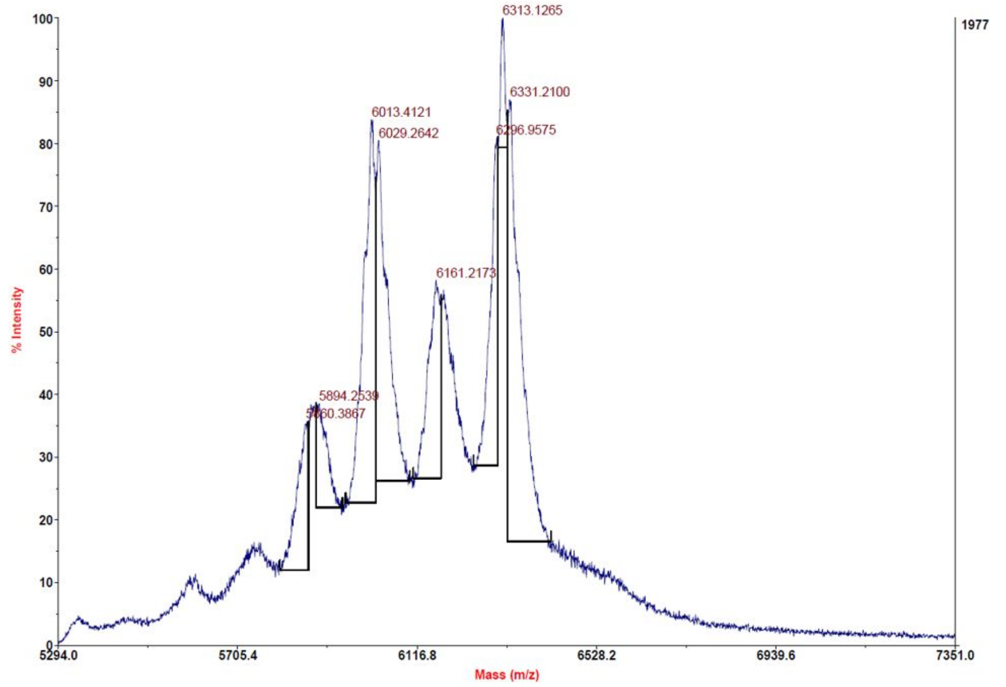

Figure S51. MALDI-TOF spectrum of t-IE2-BHQ with the peak detected at m/z 6313.1265 $[M]^+$ (calculated: 6323.41 g/mol).

Name	Sequence (5’ → 3’)
T1a-FAM	TCTAGGAGAC/**C9**/CCGCCGATTA/**C9**/CTTTCAACTT/FAM
T1b-FAM	AAGTTGAAAG/**C9**/CCGCCGATTA/**C9**/GTGATGTCAT/FAM
T1c-FAM	ATGACATCAC/**C9**/CCGCCGATTA/**C9**/GTCTCCTAGA/FAM
T2a-FAM	TCTAGGAGAC/**C9**/GAAACGACAA/**C9**/CTTTCAACTT/FAM
T2b-FAM	AAGTTGAAAG/**C9**/GAAACGACAA/**C9**/GTGATGTCAT/FAM
T2c-FAM	ATGACATCAC/**C9**/GAAACGACAA/**C9**/GTCTCCTAGA/FAM
LS-ANBP	TAATCGGCGG/**C9**/s-ANBP/AACGG/s-ANBP/GCTGGG/s-ANBP/GCCTT/s-ANBP/**C9**/TTGTCGTTTC
4625-BHQ	BHQ/AAGGCATCCCAGCCTCCGTT/BHQ

Table S3. Sequences of DNA oligonucleotides for construction of ANBP-Cage. **C9** represents spacer 9. FAM represents 6-fluorescein. s-ANBP represents ANBP molecule with a single-bond. BHQ represents black hole quencher 1.

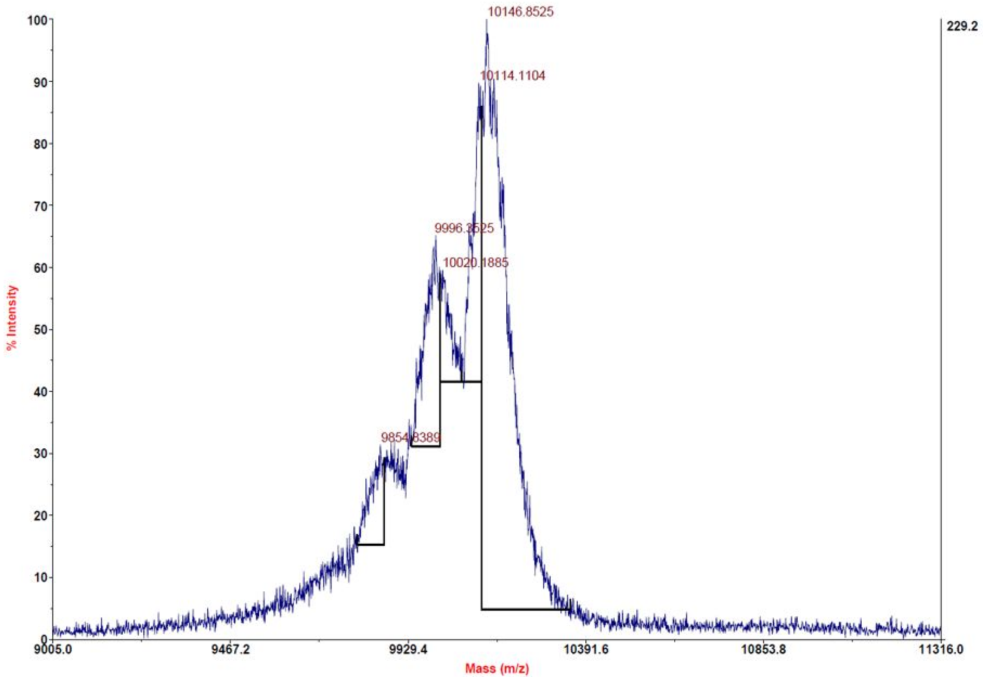

Figure S52. MALDI-TOF spectrum of T1a-FAM with the peak detected at m/z 10146.8525 $[M]^+$ (calculated: 10045.75 g/mol).

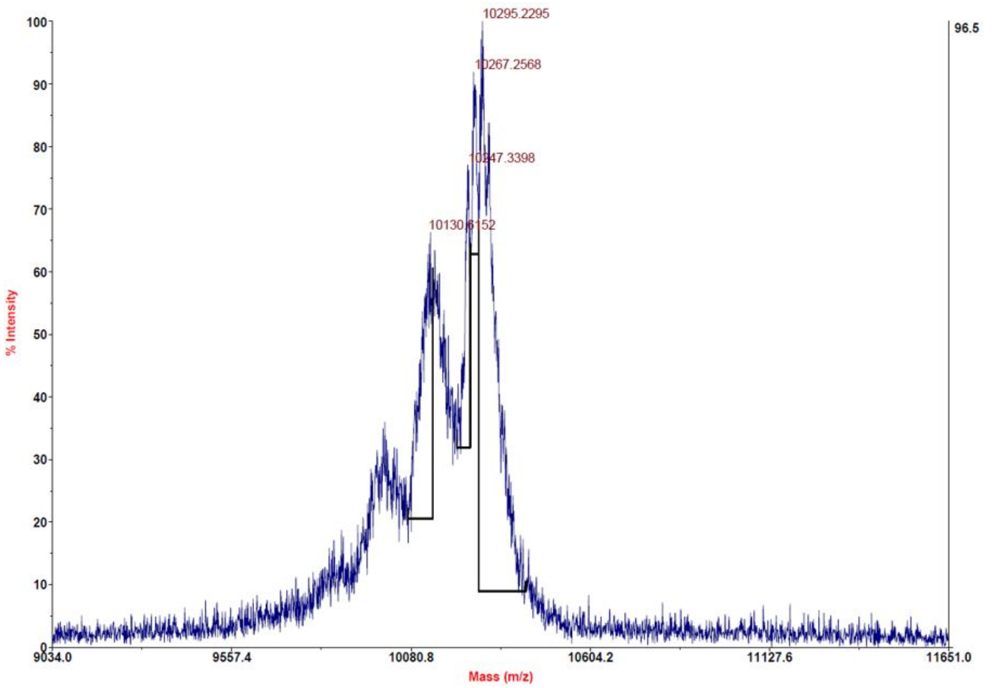

Figure S53. MALDI-TOF spectrum of T1b-FAM with the peak detected at m/z 10295.2295 $[M]^+$ (calculated: 10198.85 g/mol).

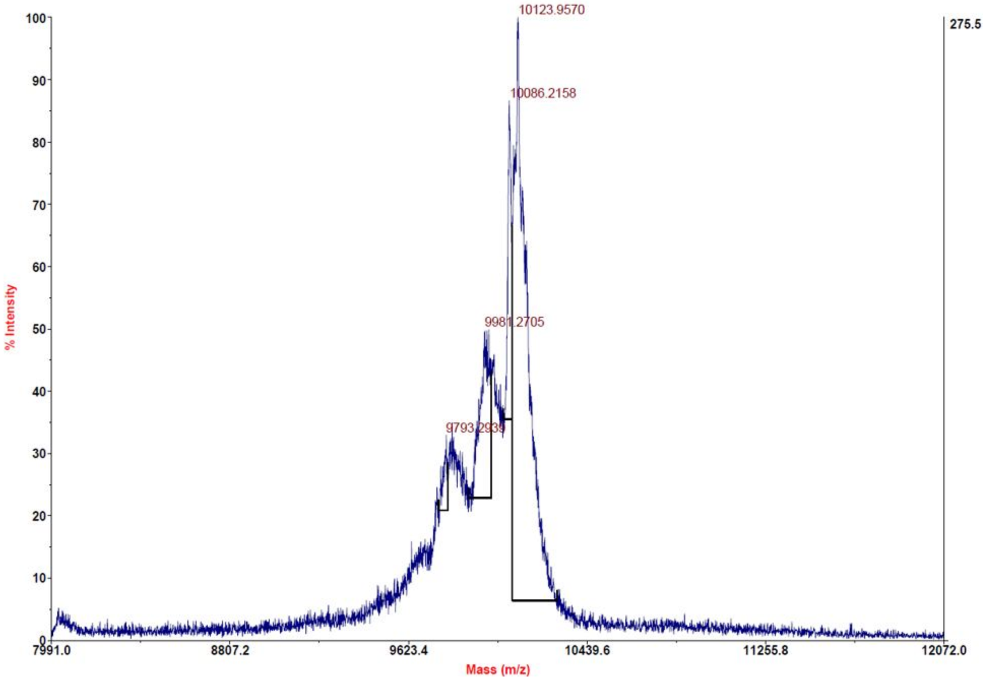

Figure S54. MALDI-TOF spectrum of T1c-FAM with the peak detected at m/z 10123.9570 $[M]^+$ (calculated: 10039.75 g/mol).

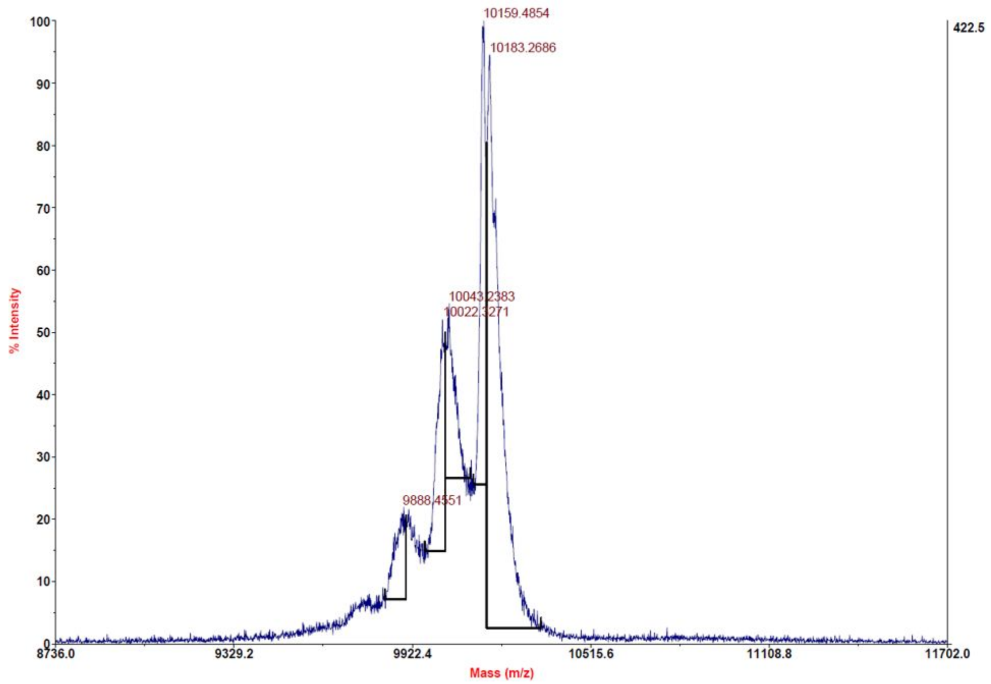

Figure S55. MALDI-TOF spectrum of T2a-FAM with the peak detected at m/z 10159.4854 $[M]^+$ (calculated: 10111.75 g/mol).

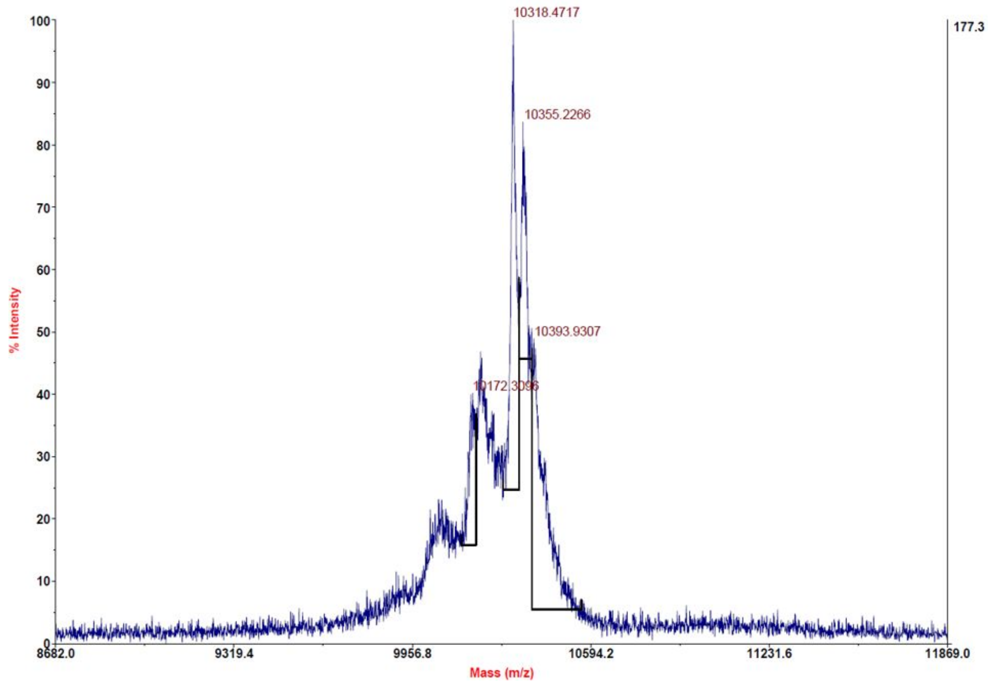

Figure S56. MALDI-TOF spectrum of T2b-FAM with the peak detected at m/z 10318.4717 $[M]^+$ (calculated: 10264.85 g/mol).

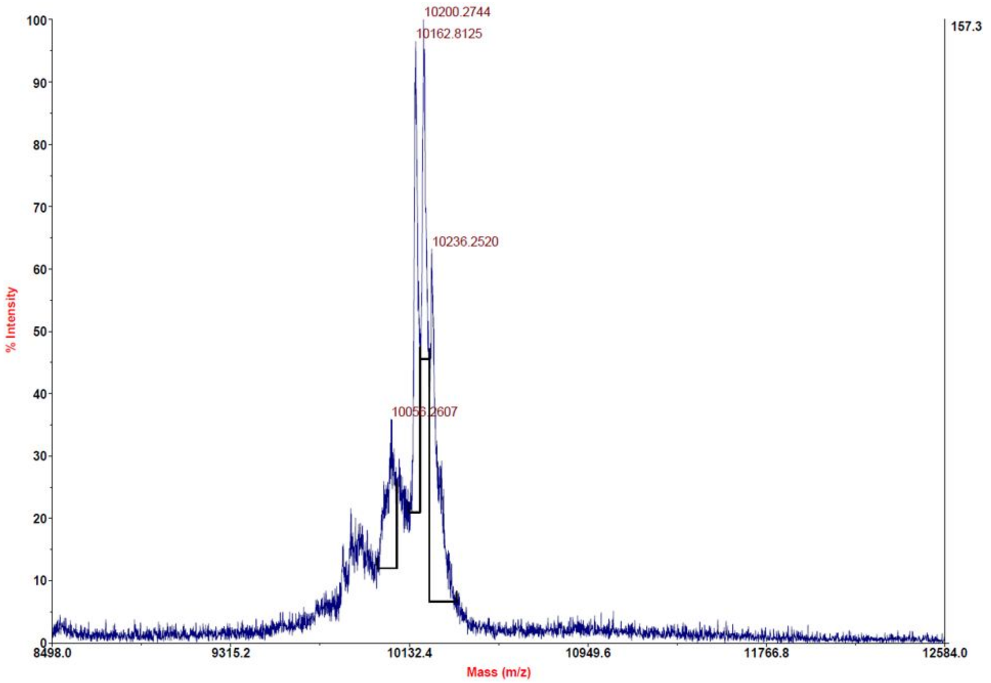

Figure S57. MALDI-TOF spectrum of T2c-FAM with the peak detected at m/z 10200.2744 $[M]^+$ (calculated: 10105.75 g/mol).

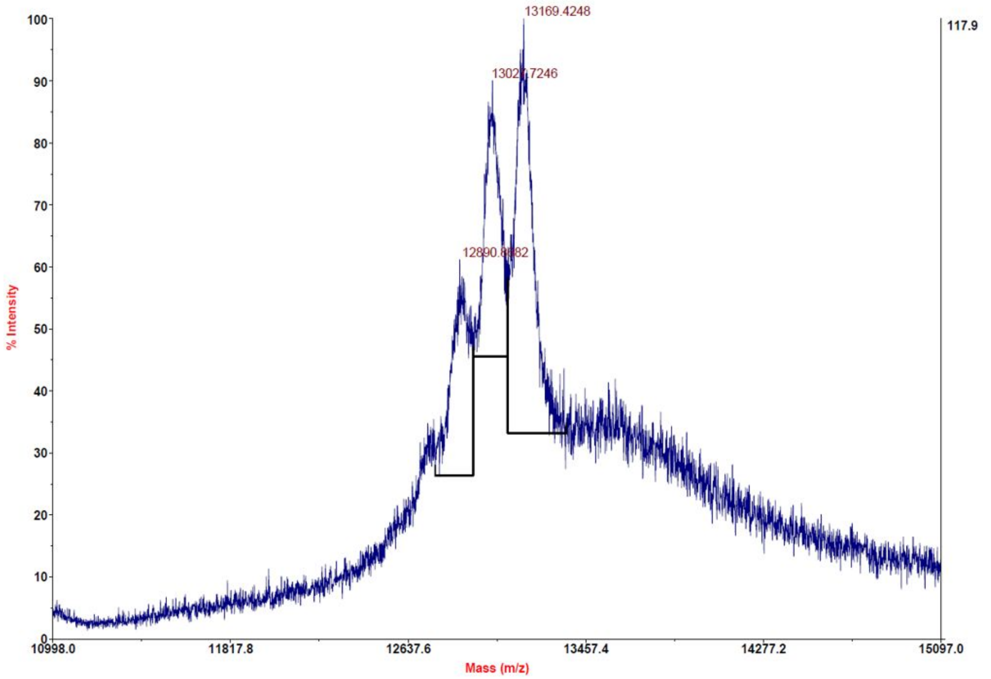

Figure S58. MALDI-TOF spectrum of LS-ANBP with the peak detected at m/z 13169.4248 $[M]^+$ (calculated: 13117.82 g/mol).

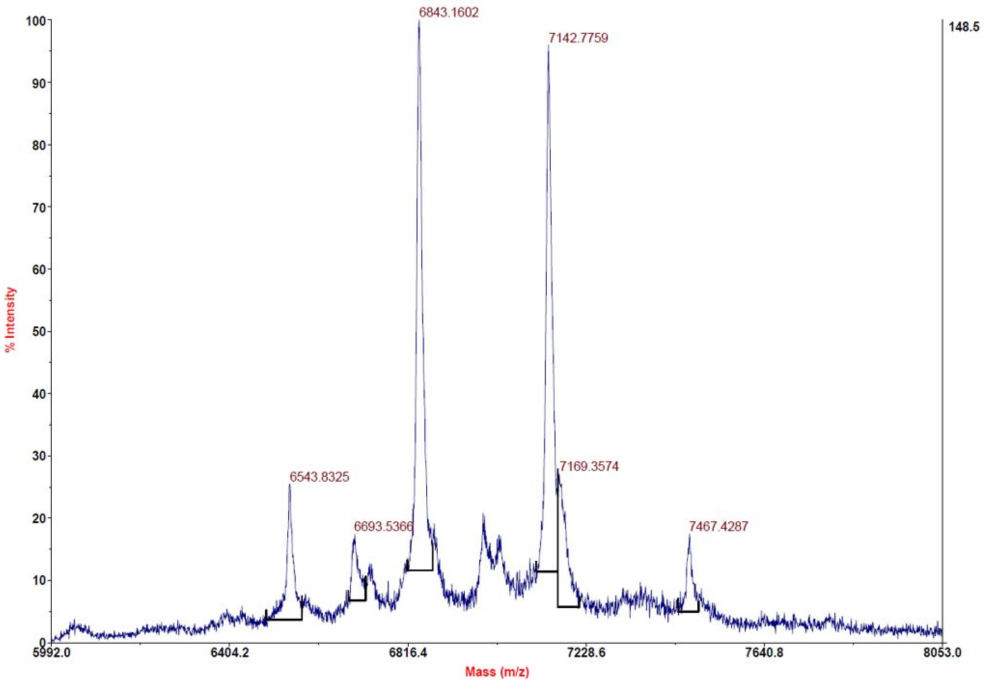

Figure S59. MALDI-TOF spectrum of ASO 4625-BHQ with the peak detected at m/z 7142.7759 $[M]^+$ (calculated: 7146.98 g/mol).

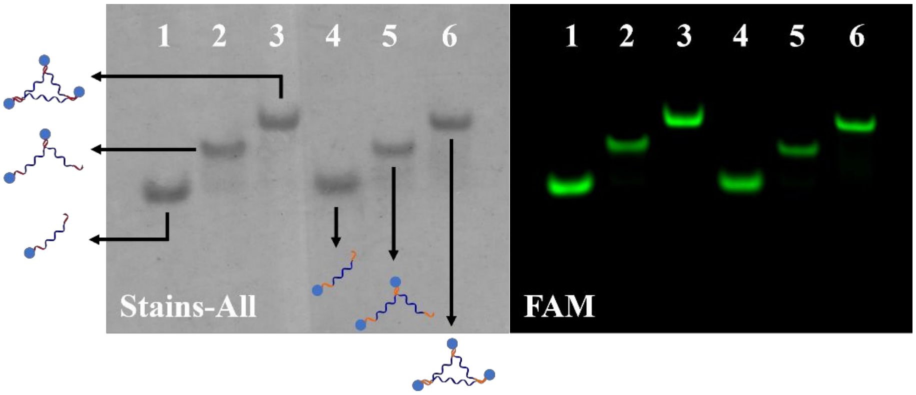

Figure 60. 8% native PAGE analysis of FAM-labeled T1 and FAM-labeled T2 assembly. Lane 1: T1a; lane 2: T1a + T1b; lane 3: T1; lane 4: T2a; lane 5: T2a + T2b; lane 6: T2. The excitation wavelength applied in the fluorescence channel was 488 nm.

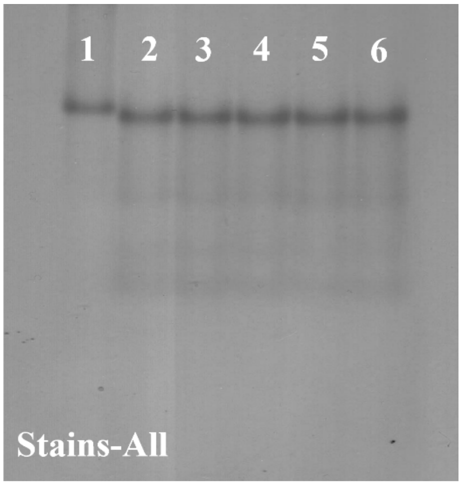

Figure S61. Enzymatic stability test of ANBP-Cage. 6.5% native PAGE analysis of ANBP-Cage incubated with DMEM supplemented with 10% FBS at 37 ^{o}C for 0.5 h (lane 2), 1 h (lane 3), 2 h (lane 4), 4 h (lane 5) and 6 h (lane 6). Lane 1 was ANBP-Cage without any treatment.

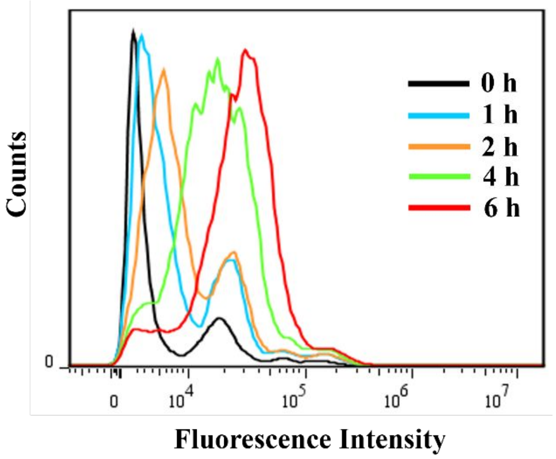

Figure S62. Flow cytometric analysis of MDA-MB-231 cells incubated with Cy3-labelled ANBP-Cage for time points of 0, 1, 2, 4 and 6 h.

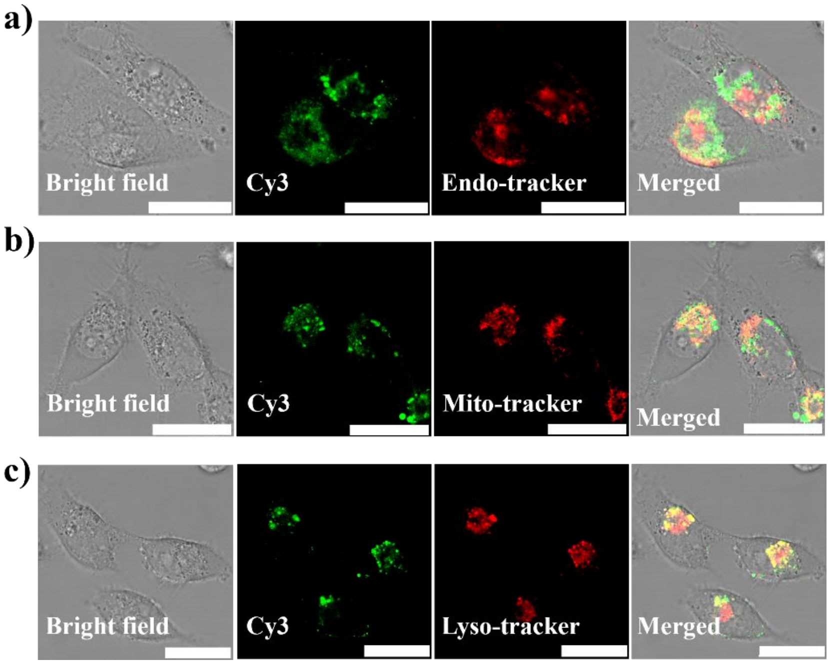

Figure S63. Confocal images of MDA-MB-231 cells incubated with Cy3-labelled ANBP-Cage and stained with a) endo-tracker, b) mito-tracker and c) lyso-tracker. The green colour indicated the fluorescence signal of Cy3 and red colour presented the fluorescence by organelle tracker. The scale bar is 25 μm.
